# Supplementary material for: Scoping review on interventions to improve adherence to reporting guidelines in health research
Source: BMJ Open. 2019 May 9;9(5):e026589. doi: 10.1136/bmjopen-2018-026589 (PMC6527996; doi:10.1136/bmjopen-2018-026589)
Supplement: Supplementary file 1 [file bmjopen-2018-026589supp001.pdf]

Acronym	Full name
CONSORT	Consolidated Standards of Reporting Trials
STROBE	Strengthening the Reporting of Observational Studies in Epidemiology
PRISMA	Preferred Reporting Items for Systematic Reviews and Meta-Analyses
SRQR	Standards for Reporting Qualitative Research
COREQ	Consolidated criteria for Reporting Qualitative research
STARD	Standard Protocol Items: Recommendations for Interventional Trials
TRIPOD	Transparent Reporting of a multivariable prediction model for Individual Prognosis or Diagnosis
SQUIRE	Standards for Quality Improvement Reporting Excellence
CHEERS	Consolidated Health Economic Evaluation Reporting Standards
SPIRIT	Standard Protocol Items: Recommendations for Interventional Trials
PRISMA-P	Preferred Reporting Items for Systematic Review and Meta-Analysis Protocols
CARE	Case Report
AGREE	Appraisal of Guidelines, Research and Evaluation
ARRIVE	Animal Research: Reporting In Vivo Experiments
RIGHT	Reporting Tool for Practice Guidelines in Health Care
QUOROM	Quality of Reporting of Meta-analyses
