# Supplementary material for: Scoping review on interventions to improve adherence to reporting guidelines in health research
Source: BMJ Open. 2019 May 9;9(5):e026589. doi: 10.1136/bmjopen-2018-026589 (PMC6527996; doi:10.1136/bmjopen-2018-026589)
Supplement: Supplementary file 2 [file bmjopen-2018-026589supp002.pdf]

Type of intervention	Intervention	Number of studies and study design	Details of the intervention	RGs implemented	Format of RG implementation	Measure of adherence to RGs	Effect on adherence to RGs*
Encouraging adherence	Implementation of the writing aid tool COBWEB (12)	1 RCT	Participants have to write the six domains of the methods section of the manuscript for the protocol they receive. They have access to COBWEB tool for a random three of the six domains.	CONSORT & CONSORT extension for non-pharmacological interventions	Bullet points and examples (6 items)	Mean score for completeness of reporting (scale 0–10, items weighted)	Difference of 2.1 (95% CI 1.5-2.7)
	Author use of a structured approach for reporting research (47)	1 Observational study (cross-sectional evaluation)	Results are posted in a standard tabular format without discussions or conclusions.	CONSORT	Checklist (4 items)	Percentage compliance of each RG item	Difference of 0.16, 0.10, 0.18 and 0.36 for each of the 4 items considered
	Journal endorsement (3 interventions, see “Details of the intervention”) (27–46,48–106,113)	80 observational studies (57 cross sectional evaluations of endorsing vs non-endorsing journals, 9 before and after evaluations of endorsing journals before and after endorsement, 14 both kind of evaluations)	A) Editorial statement endorsing certain RGs,<br>B) Recommendation or requirement to follow RGs in the "Instructions to authors", and<br>C) Requirement to submit a RG checklist together with the manuscript indicating page numbers corresponding to each item.	CONSORT (46 of 80)<br>CONSORT extensions (9 of 80)<br>QUOROM (3 of 80)<br>PRISMA (4 of 80)<br>PRISMA extensions (1 of 80)<br>STARD (11 of 80)<br>STROBE (4 of 80)<br>ARRIVE (1 of 80)<br>CONSORT, STROBE and PRISMA (11 of 80)	Checklist (all items)	For CONSORT: percentage of compliance for each item**<br><br>For other RGs: Mean summed score for completeness of reporting**	For CONSORT: 25 items improved (see details for each item on figure 2 on Turner et al. (6))<br><br>For CONSORT extension for harms: Difference of 0.04 (99% CI –1.50 to 1.58) (see Stevens et al. (3))

						For PRISMA: Difference of 0.53 (99% CI 0.02 to 1.03) (see Stevens et al. (3))<br><br>For STARD: Difference of 0.52 (99% CI –0.11 to 1.16) (see Stevens et al. (3))<br><br>For STRICTA: Difference of 1.42 (99% CI –0.04 to 2.88) (see Stevens et al. (3))<br><br>For STROBE: Difference of 1.55 (99% CI –3.19 to 6.29) (see Stevens et al. (3))
Suggestion for peer reviewers to use RGs (107)	1 RCT	Peer reviewers are sent a standard letter encouraging them to use different reporting guidelines. Reviewers are not asked to report whether they used the reporting guideline in reviewing the manuscript.	CONSORT, QUOROM, STARD	Checklist (all items)	Modified version of Manuscript Quality Assessment Instrument (scale 36-180)	Difference of 0.9 (95% CI -0.3 to +2.1)

Checking adherence and providing feedback	Completeness of reporting check by the editors (117)	1 Observational Study (Before and after evaluation)	Initial submissions are vetted by the editor-in-chief. If the submission is considered appropriate, manuscripts are assessed by the associate editor for CONSORT adherence. Authors are asked to make changes accordingly until associate editor deems appropriate that they move to the next step of the review process leading to an editorial decision.	CONSORT	Checklist (all items)	Percentage of compliance of each RG item	Before – compliance ranges from 0% to 100% (Median 40%)<br><br>After – perfect compliance in 33 out of 37 items
	Additional review against RGs (118)	1 RCT	A senior statistician does an additional review of all papers and provides authors suggestions on how to follow reporting guideline checklists.	STROBE, CONSORT, STARD	Checklist (all items)	Modified version of Manuscript Quality Assessment Instrument (scale 1 to 9)	Difference of 0.25 (95% CI<br><br>-0.05 to +0.54)
	Active implementation of RG by editors (2 interventions, see “Details of the intervention”) (13)	1 Observational study (Interrupted time series evaluation)	A) Email is sent to authors to revise the abstract according to the guidelines at the revision stage and B) Changes are made by the assistant editors of these journals towards the end of the editorial process.	CONSORT extension for abstracts	Checklist (9 of 17 items)	Monthly mean number of items reported (scale 0 to 9)	Difference of 1.5 items

	Implementation of the web-based tool WebCONSORT (119)	1 RCT	Journal editor includes a link to WebCONSORT in the revision letter to authors. Authors are directed to an automatically generated list of items and a flow diagram customised to their specific trial design.	CONSORT & some CONSORT extensions	Checklist (10 of 25 items)	Percentage of items reported for each article	Difference of 0.04 (95% CI −0.02 to +0.10)
Involvement of experts	Statistician involvement (78,128-130)	4 Observational studies (cross sectional evaluations)	Statisticians (or epidemiologists or other quantitative methodologists) are involved in the design, conduct or reporting of the study	CONSORT	Checklist (all items)	Mean score for completeness of reporting (scale 0-10, items not weighted)	In Diaz-Ordaz (78): No global effect provided (see effects for individual items in Table 2 of the paper)<br><br>In Pandis et al. (128): Difference of 0.93<br><br>In Péron et al. (129): No difference in medians<br><br>In Kloukos et al. (130): 0.27

*Difference between adherence to RGs in intervention and non-intervention group. We did not report the CI of the effect size when authors did not report it in the original papers.

**As the 80 individual studies that belong to this category used different measures of adherence to reporting guidelines, we report here the measures used in the two systematic reviews that summarized the pooled results of most of these studies (3,6).
