# Supplementary material for: Scoping review on interventions to improve adherence to reporting guidelines in health research
Source: BMJ Open. 2019 May 9;9(5):e026589. doi: 10.1136/bmjopen-2018-026589 (PMC6527996; doi:10.1136/bmjopen-2018-026589)
Supplement: Author's manuscript [file bmjopen-2018-026589.draft_revisions.pdf]

BMJ Open

BMJ Open is committed to open peer review. As part of this commitment we make the peer review history of every article we publish publicly available.

When an article is published we post the peer reviewers' comments and the authors' responses online. We also post the versions of the paper that were used during peer review. These are the versions that the peer review comments apply to.

The versions of the paper that follow are the versions that were submitted during the peer review process. They are not the versions of record or the final published versions. They should not be cited or distributed as the published version of this manuscript.

BMJ Open is an open access journal and the full, final, typeset and author-corrected version of record of the manuscript is available on our site with no access controls, subscription charges or pay-per-view fees (http://bmjopen.bmj.com).

If you have any questions on BMJ Open's open peer review process please email info.bmjopen@bmj.com

BMJ Open

A scoping review on interventions to improve adherence to reporting guidelines in health research

Journal:	*BMJ Open*
Manuscript ID	bmjopen-2018-026589
Article Type:	Research
Date Submitted by the Author:	12-Sep-2018

Complete List of Authors:	Blanco, David; Universitat Politècnica de Catalunya, Statistics and Operations Research<br>Altman, Doug; Centre for Statistics in Medicine<br>Moher, David; Ottawa Hospital Research Institute, Centre for Journalology<br>Boutron, Isabelle; Université Paris Descartes, Centre d\'épidémiologie clinique<br>Kirkham, Jamie; University of Liverpool, Biostatistics<br>Cobo, Erik; Universitat Politècnica de Catalunya, Statistics and Operations Research

Keywords:	Scoping review, Quality of reporting, Completeness of reporting, Reporting guidelines, Knowledge synthesis, Adherence

SCHOLARONE™
Manuscripts

A scoping review on interventions to improve adherence to reporting guidelines in health research

Authors

David Blanco[1,2], Doug Altman[3], David Moher[4], Isabelle Boutron[5], Jamie J Kirkham[6*] and Erik Cobo[1*].

*JJK and EC are joint senior authors.

[1]Statistics and Operations Research Department, Universitat Politècnica de Catalunya - Barcelona Tech, Barcelona, Spain.

[2]INSERM, U1153 Epidemiology and Biostatistics Sorbonne Paris Cité Research Center (CRESS), Methods of Therapeutic Evaluation of Chronic Diseases (METHODS) Team, Paris Descartes University, Sorbonne Paris Cité, Paris F-75014, France.

[3]Centre for Statistics in Medicine, University of Oxford, Oxford, UK.

[4]Centre for Journalology, Ottawa Hospital Research Institute, Ottawa, Canada.

[5]Centre d'Epidémiologie Clinique, Université Paris Descartes, Paris, France.

[6]Department of Biostatistics, University of Liverpool, Liverpool, UK.

Correspondence to David Blanco; david.blanco.tena@upc.edu

Keywords

Scoping review, knowledge synthesis, reporting guidelines, completeness of reporting, quality of reporting, adherence

Abstract

Objectives: The goal of this study is to identify, analyse and classify interventions to improve adherence to reporting guidelines in order to obtain a wide picture of how the

For peer review only - http://bmjopen.bmj.com/site/about/guidelines.xhtml

problem of enhancing the completeness of reporting of biomedical literature has been tackled so far.

Design: Scoping review.

Search strategy: We searched the MEDLINE, EMBASE, and Cochrane Library databases and conducted a grey literature search for (i) studies evaluating interventions to improve adherence to reporting guidelines in health research and (ii) other types of references describing interventions that have been performed or suggested but never evaluated. The characteristics and effect of the evaluated interventions were analysed. Moreover, we explored the rationale of the interventions identified and determined the existing gaps in research on the evaluation of interventions to improve adherence to reporting guidelines.

Results: 109 references containing 31 interventions (11 evaluated) were included. These were grouped into five categories: (1) training, (2) improved understanding, (3) encouraging adherence, (4) monitoring adherence and providing feedback, and (5) collaboration among authors and experts. Only 4 of the interventions found had been evaluated by randomised trials. Research gaps identified included the evaluation of interventions (i) on training and improved understanding of reporting guidelines, (ii) at early stages of research, and (iii) after the process of author revision of the manuscript.

Conclusions: This scoping review identifies a wide range of strategies to improve adherence to reporting guidelines that can be taken by different stakeholders. Future randomised trials should evaluate further interventions and address the research gaps identified. This review is part of a larger project whose next goals are (i) to capture editors' perceptions on the barriers and facilitators of some interventions identified in this review, (ii) to explore new interventions, and (iii) to evaluate one of these interventions in collaboration with BMJ Open.

Strengths and limitations

- We considered wide range of reporting guidelines as well as their extensions.

For peer review only

- Merging the evidence found in the published and grey literature allows us to provide a broad picture of how the problem of enhancing adherence to reporting guidelines has been tackled so far and could be faced in the future.
- The screening and data extraction were performed in duplicate.
- We could have missed evidence of possible interventions that may not be present in the published or grey literature but are instead used in practice and continue to be used.

Background

Approximately 85% of all biomedical research today is estimated to be wasted, due, in part, to incomplete or inaccurate reporting (1). The past two decades have given rise to a number of changes in an effort to help authors and the broader scientific community properly report research methods and findings, which would allow them to contribute to the broader goal of combating waste in biomedical research. The most prominent of these changes has been the inception of reporting guidelines for different study types, data, and clinical areas (2).

The vast majority of reporting guidelines have not yet been assessed as to whether they help improve the reporting of research (3), but some, such as the Consolidated Standards of Reporting Trials (CONSORT) for the reporting of randomised controlled trials (RCTs) (4), have been shown to enhance the completeness of reporting (5,6).

Dozens of systematic reviews have explored the extent of adherence to some reporting guidelines in certain areas of health research (7–10). Saaman et al. (11) went one step further and performed a systematic review of systematic reviews assessing adherence to reporting guidelines. As they considered a broad range of clinical areas and study designs, their results provided a global picture of adherence to reporting guidelines in health research. Although some studies reported acceptable overall levels of completeness of reporting and found that it had improved since the introduction of certain reporting guidelines such as CONSORT, the authors of most of the reviews (43 of 50, 86%) concluded that more improvement is needed or that adherence to reporting guidelines was inadequate, poor, medium or suboptimal. Therefore, it is

warranted to explore and develop strategies to improve the current levels of adherence to reporting guidelines.

In recent years, several initiatives aiming to improve adherence to reporting guidelines have been proposed, some of which have already been evaluated. For example, the effect of journal endorsement of reporting guidelines (3,5,6) and the implementation of writing aid tools for authors such as the CONSORT-based web tool (COBWEB) (12) have been assessed. While some of these strategies have not been shown to have a benefit (3), others report better but still suboptimal levels of reporting (5,6) or even clear benefits (12,13).

As mentioned, several reviews have analysed the quality of reporting in different clinical areas and for different study types (7–10). However, no scoping review has been performed that provides a global picture of different strategies aiming to improve adherence to reporting guidelines. Given the low levels of completeness of reporting in health research that have been observed (11), along with the imperative need to take further actions for mitigating this problem, we considered that performing such a scoping review was warranted.

In addition to analysing the implementation and effect of interventions that have already been evaluated, we aimed to gather other possible strategies that could be implemented and evaluated in the future.

For clarification, some relevant terms used throughout the scoping are defined in Box 1, which is based on Stevens et al. (3).

Box 1: relevant definitions in the context of this scoping review

For peer review only

Adherence: Action(s) taken by authors to ensure that a research report is compliant with the items recommended by the appropriate/relevant reporting guideline.

Endorsement: Action(s) taken by journals to indicate their support for the use of one or more reporting guideline(s) by authors submitting research reports for consideration.

Implementation: Action(s) taken by journals to ensure that authors adhere to an endorsed reporting guideline and that therefore published papers are completely reported.

Complete reporting: Pertains to the state of reporting of a study report and whether it is compliant with all the items recommended by the appropriate/relevant reporting guideline.

Methods

As presented in the published protocol (14), this scoping review follows the methodology manual published by the Joanna Briggs Institute for scoping reviews (15).

Objectives

The scoping review questions are:

1. What interventions to improve adherence to reporting guidelines in health research have been evaluated?
2. What further interventions to improve adherence to reporting guidelines have been performed or suggested but never evaluated?

We aim to analyse and classify the interventions found for both questions in order to obtain a wide picture of how the problem of adhering better to reporting guidelines has been tackled so far and can be tackled in the future.

Eligibility criteria

We included:

1. Studies evaluating interventions aiming to improve adherence to reporting guidelines in health research, irrespective of study design.

For peer review only

2. Commentaries, editorials, letters, studies, and online sources describing possible interventions to improve adherence to reporting guidelines that have been performed or suggested but never evaluated.

The reporting guidelines considered were those shown on 8 May 2017 on the EQUATOR (Enhancing the QUAlity and Transparency Of Health Research) Network website (16) as "Reporting Guidelines for main study types" (see Supplementary file 1). In addition, we also included QUOROM (Quality of Reporting of Meta-analyses), since it was the precursor of PRISMA.

We considered the following languages: English, Spanish, French, German and Catalan.

Exclusion criteria

We have excluded references that include interventions that do not specifically aim to improve the completeness of reporting, even though these interventions may actually influence completeness. For example, we have excluded clinical trial registration even though it may enhance completeness of reporting, because its main goals are to improve clinical trial transparency while also reducing publication and selective reporting biases.

Search strategy and study selection

On 8 May 2017, we searched PubMed, EMBASE, and Cochrane Library databases for articles published between 1 January 1996 and 31 March 2017, in accordance with our scheduled search (14). The detailed search terms for PubMed can be found in Supplementary file 2.

The retrieved studies were exported into Mendeley and duplicates were removed. One reviewer (DB) first screened the titles and abstracts for eligibility. Each of the other two reviewers (JJK and EC) was randomly assigned 50% of the references and screened the titles and abstracts independently of the first reviewer. The reviewers classified the references into one of the following groups:

For peer review only

A) Evaluated: Includes references describing interventions to improve adherence to reporting guidelines that have been empirically assessed.

B) Non-evaluated: Includes references describing interventions to improve adherence to reporting guidelines that have been performed or suggested but never evaluated.

C) Unclear: Includes references (i) containing vague statements such as "Authors, editors, and journals have to adhere better to reporting guidelines to improve the quality of reporting" or "greater efforts have to be made by authors to check that their research is compliant with [the relevant reporting guideline]", or (ii) not having the abstract available.

D) Excluded: Includes references (i) not describing interventions to improve adherence to any of the reporting guidelines considered and (ii) describing but not evaluating certain interventions that have already been classified as evaluated.

Disagreements were solved by discussion among the reviewers.

Second, one reviewer (DB) examined the full-text of all group A and B references to confirm the previous classification, then all group C references to reclassify them either as group A, B, or D. Re-classification was verified by the initial reviewer (JJK or EC). Finally, one reviewer (DB) ensured literature saturation by searching the reference lists of included studies, the lists of articles citing them according to PubMed, and the individual studies included in two relevant systematic reviews (3,6).

In addition, we performed a grey literature search, which included: the websites of networks and organizations promoting the use of reporting guidelines (i.e., EQUATOR Network and National Library of Medicine Research Reporting Guidelines and Initiatives); work groups of medical journal editors (i.e., International Committee of Medical Journal Editors (ICMJE) and World Association of Medical Editors (WAME)); biomedical journal publishers (i.e., BMJ Publishing Group and BioMed Central); funding agencies (i.e., National Institute of Health (NIH) and European Research Council); online platforms of post-publication peer review (i.e., PubPeer and ScienceOpen); and

For peer review only

the abstract books of the past editions of the International Congress on Peer Review and Biomedical Publication.

Data extraction

A data extraction form was developed to collect the information necessary for data synthesis. In order to better capture some further relevant aspects of the included references, the original data extraction form proposed in the protocol was updated. Two reviewers (DB, JJK) independently performed a pilot data extraction on a random sample of 5 articles and subsequently refined the form.

Extracted data included:

1. Publication characteristics: title, year of publication, author, author's affiliation country, and field of study.
2. Characteristics of the intervention:
    a. Classification as evaluated or non-evaluated.
    b. Research stage: education, grant writing, protocol writing, manuscript writing, submission, journal peer review, author revision, copy-editing, and post-publication.
    c. Rationale of the intervention, which refers to the deduced reasons why the intervention is evaluated or proposed.
    d. For evaluated interventions: details of the intervention, study design (e.g. RCT, before-after, etc.), reporting guidelines considered and format (checklist, bullet points and/or examples), period of intervention, number of journals and articles involved, effect size of the intervention on adherence to reporting guidelines and measure used to assess this effect.
3. Relevant conclusions.

Two reviewers (DB, JJK) independently performed data extraction for all studies except for the individual studies of the two systematic reviews evaluating journal endorsement of reporting guidelines (3,6), since none of these studies described

For peer review only

further interventions and their results had already been reported in these reviews. Discrepancies between reviewers were discussed and solved by consensus.

Data synthesis

Following data extraction, interventions to improve adherence to reporting guidelines were categorised as follows:

1. Training: mentoring of different stakeholders on the practical use of reporting guidelines.
2. Improved understanding: in-depth focus on the content and requirements of reporting guidelines.
3. Encouraging adherence: suggestions and tools to facilitate compliance.
4. Monitoring adherence and providing feedback: checking the level of compliance and indicating incorrect or missing items.
5. Collaboration among authors and experts: interaction and cooperation on methodology and reporting.

One reviewer (DB) performed the initial categorization, which was verified and refined by the other two reviewers (JJK and EC).

Furthermore, we determined the existing gaps in research on the evaluation of interventions to improve adherence to reporting guidelines. More specifically, we identified which categories of interventions and which research stages have not been addressed so far in studies evaluating interventions.

Patients and public involvement

No patients or public were involved in the study.

Results

The database search yielded 1399 citations after deduplication (see Figure 1). Screening of titles and abstracts resulted in a first classification, after which 435 papers were included for full text review. We also reviewed the full text of 24 additional references found through forward citation searching. Furthermore, a grey literature

For peer review only

search yielded 7 additional references. Finally, 109 references were included. 90 of them (86 observational and 4 randomised studies) described 11 evaluated interventions and the other 19 (12 research studies, 2 editorials, 2 blogs, 1 commentary, 1 essay, and 1 perspective) described 20 non-evaluated interventions.

Among all included references, we identified 31 interventions to improve adherence to reporting guidelines. Some of these interventions appeared in more than one reference and some of the references contained more than one intervention. From those 31 interventions, 4 were categorised as "Training" (1,17-21), 2 as "Improved understanding" (22,23), 15 as "Encouraging adherence" (11,12,20,24,25,27,29-114), 8 as "Monitoring adherence and providing feedback" (13,115-117,119,121,122,125), and 2 as "Collaboration among authors and experts" (25,84,126-128). Figure 2 displays all those interventions according to their categorization and the research stage where they can be performed. Evaluated interventions are highlighted in bold. Supplementary file 3 provides further details of the implementation of the evaluated interventions.

Research gaps identified (see Figure 3) included the evaluation of interventions (i) on training and improved understanding of reporting guidelines, and (ii) at early stages of research (education, grant writing or protocol writing), and (iii) after the process of author revision of the manuscript (copyediting or post-publication peer review).

Hereafter, we present a brief description of the interventions found for each category. For the sake of clarity, the rationale of those interventions is shown in Table 1.

Training

In a first step, health profession schools could incorporate reporting guidelines into curricula that address research methodology and publication standards (17–21). In line with this, students could develop protocols for coursework and research using reporting guidelines such as SPIRIT (randomised trials) and PRISMA-P (systematic reviews), and educators may encourage adherence to those guidelines and grade the protocols using them (20). For their part, funders may consider supporting author training on reporting guidelines (1). Finally, journals or publishers may consider

investing resources in training editors and reviewers on the content and use of reporting guidelines (1,21).

Improved understanding

Reporting guideline developers might consider translating them to new languages that have not been addressed yet (22). Also, further databases of examples of good reporting for different reporting guidelines that are accessible to authors can be developed, as has been done for CONSORT (23).

Encouraging adherence

First, international scientific associations may play an important role in disseminating and popularizing reporting guidelines to large audiences (24). For their part, funders might require authors to use reporting guidelines as a template for grant application proposals (20). Later on, research ethics boards may require that protocols submitted for ethical approval clearly state which reporting guidelines the study will be using based on the study design, and that reporting guideline checklists are part of the application for ethics approval (11). Funders could also encourage adherence to reporting guidelines by asking for reporting guideline checklists as part of the authors' report (20,25).

One initiative to support author to adhere to reporting guidelines at the writing stage of the manuscript has been COBWEB, a writing aid tool that aims to help authors adequately combine the different extensions of the CONSORT statement (12). This tool divided the CONSORT items into bullet points showing the key elements that need to be reported together with examples of adequate reporting. The impact of COBWEB was evaluated through a randomised trial that showed a large effect of this tool, with a mean improvement in CONSORT scores of 2.1 on a 0–10 scale for the intervention group as compared to the control group (12). A second option to support authors at manuscript writing might be to follow a more structured approach including new subheadings, boxes and tables with key information for different kinds of study designs. For example, ClinicalTrials.gov requires a structured approach to register a study or to report its results (26). This has been shown to be effective: some results

For peer review only

posted on clinicaltrials.gov, especially harms, are more complete than those in corresponding journal articles reporting the same trials (27). In line with this, the American Journal of Orthodontics and Dentofacial Orthopedics (AJO-DO) updated the traditional RCT format and made readily available a sample article including comments and instructions (28). Finally, another option to help authors avoid omissions when writing the manuscript could be that they mark up the text and show where each item of the relevant checklist is addressed (29).

At manuscript submission stage, different editorial actions have been taken to improve adherence to reporting guidelines. The most popular is what has traditionally been defined as journal endorsement of reporting guidelines, which is usually defined as one or more of the three following interventions: (a) journal editorial statement endorsing certain reporting guidelines; (b) requirement or recommendation in journal's 'Instructions to Authors' to follow certain reporting guidelines when preparing their manuscript; or (c) requirement for authors to submit the appropriate reporting guideline checklist together with their manuscript indicating page numbers corresponding to each item (6). Dozens of observational studies have explored the possible effect of journal endorsement of different reporting guidelines in different clinical areas (30–109). A recent systematic review focused on CONSORT evaluations showed relative but suboptimal improvements in the completeness of reporting in journals by following the aforementioned policies (6), while another systematic review considering 9 other guidelines showed no improvements (3).

Journals might also consider other strategies to enhance adherence to reporting guidelines at submission. A first option could be to develop shorter, core versions of reporting guidelines containing key items (110). Second, they might introduce publication officers in order to provide guidance to authors on preparing manuscripts for submission (111). Third, they may ask authors to populate the relevant checklist with text from their report and not accept a submission unless this is provided (112).

Finally, editors may suggest that peer reviewers use reporting guidelines (113). In addition, by asking peer reviewers questions about whether the author has followed reporting guidelines, this might be an indirect way to encourage them (114).

For peer review only

Monitoring adherence and providing feedback

Having recognised that journal endorsement without implementation does not guarantee adherence to reporting guidelines, some journals have opted for implementing reporting guidelines at peer review. First, an associate editor may assess manuscripts for adherence to the relevant reporting guideline and ask authors to make changes accordingly (115). This process may be repeated until the associate editor thinks that the manuscript can move to the next step of the review process, leading to an editorial decision. This intervention was evaluated at the AJO-DO and showed satisfactory results: 33 of 37 items reached perfect compliance (115). Second, peer reviewers could also assess the manuscripts against the appropriate checklist (116). While the observed effect of this intervention was slightly positive, it was smaller than hypothesized. In fact, investigators pointed out that authors tended to comply better with suggestions coming from standard reviews rather than from reviews against reporting guidelines, implying that it might be difficult to adhere to high methodological standards at late stages of research if these standards are not considered earlier in the research process. Third, journals could also ask trained editorial assistants to check manuscripts against reporting guidelines (117) or to implement automatic peer review tools such as Statreviewer (118), software that automatically checks adherence to reporting guidelines and evaluates the appropriate use and reporting of statistical tests (119). Currently, its performance is being assessed through a pilot trial in collaboration with four BioMed Central Journals (119). In any of those cases, emails could be sent to authors asking them to revise the manuscript according to guidelines (13). To do this, the EQUATOR Network has provided standard letters that can be used a) after checks by an editor or a single peer reviewer, b) after full peer review, or c) alongside acceptance (120). Furthermore, at the time of author revision of the manuscript, Hopewell et al. found no significant effect when incorporating WebCONSORT, a web-based tool that generates a unique list of items customised to the trial design, to the revision process of journals that endorsed CONSORT but had no active policy for implementing it (121). Finally, in a late stage of the publication process, copyediting of the manuscript could also ensure that all items are covered (122).

For peer review only

Once the paper is published, the scientific community could use online platforms of post-publication peer review such as PubPeer (123) or ScienceOpen (124) to evaluate the adherence to reporting guidelines of published articles and to provide feedback to authors (125).

Collaboration among authors and experts

Authors may benefit from collaboration with experts at different stages of research. On the one hand, the involvement of statisticians (or epidemiologists or other quantitative methodologists) in the design, conduct or reporting of the study might contribute to properly reporting key areas such as sample size calculation, randomization, blinding, and appropriate statistical analysis (126). While one study did not find a statistically significant positive relationship between CONSORT scores and statistician involvement (127), others did (84,126,128). On the other hand, it has been hypothesized that the involvement of medical writers during the manuscript writing stage of research could improve the completeness of reporting (25).

Discussion

In this scoping review, we identified 31 interventions to improve adherence to reporting guidelines. We have also determined the gaps in research on the evaluation of this type of interventions. By considering a wide range of reporting guidelines as well as their extensions and merging the evidence found in the published and grey literature, this review provides a broad picture of how the problem of enhancing adherence to reporting guidelines has been tackled so far and could be faced in the future.

This study reveals that it is primarily journals that have made most of the efforts to improve adherence to reporting guidelines in health research – although they can certainly do more. Typically, their strategies range from making available editorial statements that endorse certain reporting guidelines, recommending or requiring authors to follow reporting guidelines in the “Instructions to authors”, and requiring authors to submit a reporting guideline checklist together with the manuscript, with page numbers indicated for each item. However, these strategies have been shown

For peer review only

not to have the desired effect (3,6,129). Recent research has called for more active and enforced journal policies throughout the editorial process, such as requiring the use of structured approaches with new subheads adapted to different kinds of study designs (27), which was also found to be beneficial in a new study outside of our search period (130); providing guidance on manuscript preparation (111); making sure the peer review process involves editorial assistants who have specific training on reporting issues (117); and implementing automatic peer review tools (119). Journals will vary in their ability to make some of these strategies effective, depending on factors such as their resources, their guidelines to peer reviewers and the dedication of their editors – many editors and editorial staff work part-time and have limited amount of time.

Moreover, editors' education and performance should be improved. A recent study pointed out that more than a third (39%) of the manuscripts classified as randomised trials by the editorial staff were not actually randomised trials (121,131). Consequently, it seems difficult to improve author and peer reviewer adherence to reporting guidelines if journal gatekeepers are not properly trained in methodological and reporting issues.

Apart from journals, editors and peer reviewers, other key stakeholders such as medical schools, research funders, universities and other research institutions should also take responsibility regarding this issue. This scoping review provides some strategies to follow. However, as the problem is complex and the possible interventions are varied, enhancing the completeness of reporting most likely depends not so much on any isolated action but on a set of strategies by several different stakeholders. These could be enacted at different stages of research, from education to article post-publication.

For interventions aiming to improve adherence to reporting guidelines, we should require the same level of evidence that we require for interventions to improve health. For this reason, it is striking that we found only 4 published randomised trials that evaluated interventions to improve adherence to reporting guidelines (12,113,116,121). Among these trials, statistically significant effect of the intervention was only observed for the use of the writing aid tool for authors COBWEB (12). While

For peer review only

performing an additional review against reporting guidelines showed slightly positive but not significant effect (116), suggesting the use of reporting guidelines to peer reviewers (113) or implementing at the process of author revision of the manuscript the web-based tool WebCONSORT showed no benefit (121). The rest of the evaluations of interventions found (86 of 90) were observational studies, whose results are subject to the influence of confounding factors (6). For example, evaluations of the effect of journal endorsement may be influenced by whether different journals are actively checking that authors adhere to the requirements or recommendations they provide to authors at submission (6). For all these reasons, future randomised trials should be performed to evaluate further interventions to improve adherence to reporting guidelines. Moreover, these trials might consider addressing some of the research gaps identified in this review, such as improving adherence to reporting guidelines at the grant application or protocol writing stages.

A few of the interventions found in this review were shown to enhance adherence to reporting guidelines. However, it is noteworthy there is no evidence that some successful interventions (12,27,115) have been implemented more widely later. For this reason, more resources and efforts are needed to further implement these interventions in other settings, evaluate the effect, and share the results with the scientific community. In any case, it is important to keep in mind that contemporary publication culture may harm the potential improvements in reporting quality. This could result from the fact that most scientists feel that the primary evaluation tool of their research is the quantity of their scientific output rather than its quality (132); and such attitudes may undermine the potential effect of any intervention to improve adherence to reporting guidelines.

Our scoping review has some limitations. First, we did not formally assess the methodological quality of the studies that evaluated interventions. Second, restricting to certain databases or not having standard search terms for the databases searched may have excluded relevant publications. Third, it is possible that we could have missed evidence of possible interventions that may have never been reflected in the published or grey literature but are instead used in practice and continue to be used.

For peer review only

For example, journals might be applying specific editorial strategies that are not publicly available on their websites or in the published literature.

Conclusion

This review is part of a larger project whose next goals are (i) to capture editors' perceptions on the barriers and facilitators of some promising interventions identified in this review, (ii) to explore new possible interventions, and (iii) to evaluate one of these interventions in collaboration with BMJ Open.

Improving adherence to reporting guidelines is one of the key issues in order to enhance complete and accurate reporting and therefore reduce waste in research. For example, a decrease in waste of research from 85% to 70% would double the output of valuable research and innovation.

Different stakeholders – such as research funders, ethics boards, and journals – should identify, implement and evaluate further interventions to improve adherence to reporting guidelines.

List of abbreviations

AJO-DO: American Journal of Orthodontics and Dentofacial Orthopedics; CONSORT: CONsolidated Standards Of Reporting Trials; COBWEB: CONSORT-based web tool; EQUATOR: Enhancing the QUAlity and Transparency Of Health Research; RCT: Randomised Controlled Trial; RG: Reporting Guideline; SPIRIT: Standard Protocol Items: Recommendations for Interventional Trials; PRISMA: Preferred Reporting Items for Systematic Reviews and Meta-Analyses

Declarations

Funding: This scoping review belongs to the ESR 14 research project from the Methods in Research on Research (MiRoR) project (http://miror-ejd.eu/), which has received funding from the European Union's Horizon 2020 research and innovation programme under the Marie Sklodowska-Curie grant agreement No 676207. DM is supported through a University Research Chair (University of Ottawa).

For peer review only - http://bmjopen.bmj.com/site/about/guidelines.xhtml

Competing interests: DA and DM are Directors of the UK and Canadian EQUATOR Centres, respectively. IB is deputy director of French EQUATOR Centre.

Author contributions: All authors contributed to conceptualizing and designing the study. DB, EC, and JJK independently performed screening. DB and JJK independently performed data extraction. DB performed initial data synthesis and EC, IB, DM, DGA, and JJK refined it. DB drafted the manuscript. EC, IB, DM, DGA, and JJK made major revisions. All authors read and approved the final version of the manuscript.

Availability of data and materials: The datasets used and/or analysed during the current study are available from the corresponding author on reasonable request.

Acknowledgements: The authors thank the MiRoR Project (http://miror-ejd.eu/) and Marie Sklodowska-Curie Actions for their support. The authors also thank Matt Elmore for editorial help.

References

1. Glasziou P, Altman DG, Bossuyt P, Boutron I, Clarke M, Julious S, et al. Reducing waste from incomplete or unusable reports of biomedical research. Lancet [Internet]. 2014 Jan 18 [cited 2017 Sep 29];383(9913):267–76. Available from: http://www.ncbi.nlm.nih.gov/pubmed/24411647

2. EQUATOR Network. Library for health research reporting. Available from: http://www.equator-network.org/resource-centre/library-of-health-research-reporting

3. Stevens A, Shamseer L, Weinstein E, Yazdi F, Turner L, Thielman J, et al. Relation of completeness of reporting of health research to journals' endorsement of reporting guidelines: systematic review. BMJ [Internet]. 2014 Jun 25 [cited 2017 Jun 7];348(jun25 2):g3804–g3804. Available from: http://www.bmj.com/cgi/doi/10.1136/bmj.g3804

4. Schulz KF, Altman DG, Moher D, CONSORT Group. CONSORT 2010 statement: updated guidelines for reporting parallel group randomised trials. BMJ [Internet]. 2010 Mar 23 [cited 2018 Mar 3];340:c332. Available from:

For peer review only

http://www.ncbi.nlm.nih.gov/pubmed/20332509

5. Plint AC, Moher D, Morrison A, Schulz K, Altman DG, Hill C, et al. Does the CONSORT checklist improve the quality of reports of randomised controlled trials? A systematic review. Med J Aust [Internet]. 2006 Sep 4 [cited 2017 Feb 24];185(5):263–7. Available from: http://www.ncbi.nlm.nih.gov/pubmed/16948622

6. Turner L, Shamseer L, Altman DG, Schulz KF, Moher D. Does use of the CONSORT Statement impact the completeness of reporting of randomised controlled trials published in medical journals? A Cochrane review. Syst Rev [Internet]. 2012 Nov 29 [cited 2017 Feb 24];1(1):60. Available from: http://systematicreviewsjournal.biomedcentral.com/articles/10.1186/2046-4053-1-60

7. Bereza BG, Machado M, Einarson TR. Assessing the reporting and scientific quality of meta-analyses of randomized controlled trials of treatments for anxiety disorders. Ann Pharmacother [Internet]. 2008 Oct 26 [cited 2017 Feb 23];42(10):1402–9. Available from: http://www.embase.com/search/results?subaction=viewrecord&from=export&id=L352440213

8. Fung AE, Palanki R, Bakri SJ, Depperschmidt E, Gibson A. Applying the CONSORT and STROBE statements to evaluate the reporting quality of neovascular age-related macular degeneration studies. Ophthalmology [Internet]. 2009 Feb [cited 2017 Jun 26];116(2):286–96. Available from: http://linkinghub.elsevier.com/retrieve/pii/S0161642008009020

9. Rios LP, Odueyungbo A, Moitri MO, Rahman MO, Thabane L. Quality of reporting of randomized controlled trials in general endocrinology literature. J Clin Endocrinol Metab [Internet]. 2008 Oct [cited 2017 Feb 24];93(10):3810–6. Available from: http://press.endocrine.org/doi/10.1210/jc.2008-0817

10. Shea B, Bouter LM, Grimshaw JM, Francis D, Ortiz Z, Wells GA, et al. Scope for

For peer review only

improvement in the quality of reporting of systematic reviews. From the Cochrane Musculoskeletal Group. J Rheumatol [Internet]. 2006 Jan [cited 2017 Feb 24];33(1):9–15. Available from: http://www.ncbi.nlm.nih.gov/pubmed/16267878

11. Samaan Z, Mbuagbaw L, Kosa D, Borg Debono V, Dillenburg R, Zhang S, et al. A systematic scoping review of adherence to reporting guidelines in health care literature. J Multidiscip Healthc [Internet]. 2013 May [cited 2017 Jun 26];6:169–88. Available from: http://www.dovepress.com/a-systematic-scoping-review-of-adherence-to-reporting-guidelines-in-he-peer-reviewed-article-JMDH

12. Barnes C, Boutron I, Giraudeau B, Porcher R, Altman DG, Ravaud P. Impact of an online writing aid tool for writing a randomized trial report: the COBWEB (Consort-based WEB tool) randomized controlled trial. BMC Med [Internet]. 2015 Sep 15 [cited 2017 Feb 24];13(1):221. Available from: http://bmcmedicine.biomedcentral.com/articles/10.1186/s12916-015-0460-y

13. Hopewell S, Ravaud P, Baron G, Boutron I. Effect of editors' implementation of CONSORT guidelines on the reporting of abstracts in high impact medical journals: interrupted time series analysis. 2012 Jun 22 [cited 2017 Jan 16];344(jun22 1). Available from: http://www.ncbi.nlm.nih.gov/pubmed/22730543

14. Blanco D, Kirkham JJ, Altman DG, Moher D, Boutron I, Cobo E. Interventions to improve adherence to reporting guidelines in health research: a scoping review protocol. BMJ Open [Internet]. 2017;7(11):e017551. Available from: http://bmjopen.bmj.com/lookup/doi/10.1136/bmjopen-2017-017551

15. Reviewers' Manual. 2015; Available from: www.joannabriggs.org

16. EQUATOR Network. Available from: http://www.equator-network.org/

17. Ma B, Qi G, Lin X, Wang T, Chen Z, Yang K. Epidemiology, quality, and reporting characteristics of systematic reviews of acupuncture interventions published in Chinese journals. J Altern Complement Med [Internet]. 2012 Sep [cited 2017 Jul

For peer review only

17];18(9):813–7. Available from: http://online.liebertpub.com/doi/abs/10.1089/acm.2011.0274

18. Larson EL, Cortazal M. Publication guidelines need widespread adoption. J Clin Epidemiol [Internet]. 2012 Mar [cited 2017 Jul 21];65(3):239–46. Available from: http://linkinghub.elsevier.com/retrieve/pii/S0895435611002290

19. Sarkis-Onofre R, Cenci MS, Moher D, Pereira-Cenci T. Research Reporting Guidelines in Dentistry: A Survey of Editors. Braz Dent J [Internet]. 2017 [cited 2017 Nov 9];28(1):3–8. Available from: http://dx.doi.org/10.1590/0103-6440201601426

20. Shamseer L, Moher D, Clarke M, Ghersi D, Liberati A, Petticrew M, et al. Preferred reporting items for systematic review and meta-analysis protocols (PRISMA-P) 2015: elaboration and explanation. BMJ [Internet]. 2015 Jan 2 [cited 2017 Feb 24];349(jan02 1):g7647–g7647. Available from: http://www.bmj.com/cgi/doi/10.1136/bmj.g7647

21. Simera I, Moher D, Hirst A, Hoey J, Schulz KF, Altman DG. Transparent and accurate reporting increases reliability, utility, and impact of your research: reporting guidelines and the EQUATOR Network. BMC Med [Internet]. 2010;8(1):24. Available from: http://bmcmedicine.biomedcentral.com/articles/10.1186/1741-7015-8-24

22. Kim KH, Kang JW, Lee MS, Lee J-D. Assessment of the quality of reporting in randomised controlled trials of acupuncture in the Korean literature using the CONSORT statement and STRICTA guidelines. BMJ Open [Internet]. 2014 Jul 29 [cited 2017 Jul 17];4(7):e005068. Available from: http://bmjopen.bmj.com/cgi/doi/10.1136/bmjopen-2014-005068

23. Moher D, Hopewell S, Schulz KF, Altman DG. Resources for authors of reports of randomized trials: harnessing the wisdom of authors, editors, and readers. Trials [Internet]. 2011;12(1):98. Available from: http://trialsjournal.biomedcentral.com/articles/10.1186/1745-6215-12-98

For peer review only

24. Verbeek J. Moose Consort Strobe and Miame Stard Remark or how can we improve the quality of reporting studies. Scand J Work Environ Health [Internet]. 2008 Jun [cited 2017 Feb 24];34(3):165–7. Available from: http://www.ncbi.nlm.nih.gov/pubmed/18728905

25. Implementing Reporting Guidelines: Why and How, for Journal Editors [Internet]. World Association of Medical Editors. Available from: https://wame.blog/2017/09/17/implementing-reporting-guidelines-why-and-how-for-journal-editors/

26. ClinicalTrials.gov. National Library of Medicine (US). Available from: https://clinicaltrials.gov/

27. Riveros C, Dechartres A, Perrodeau E, Haneef R, Boutron I, Ravaud P. Timing and Completeness of Trial Results Posted at ClinicalTrials.gov and Published in Journals. [cited 2018 Mar 4]; Available from: http://journals.plos.org/plosmedicine/article/file?id=10.1371/journal.pmed.1001566&type=printable

28. Pandis N, Turpin DL. Enhancing CONSORT compliance for improved reporting of randomized controlled trials. Am J Orthod Dentofac Orthop [Internet]. 2014 Jan [cited 2017 Jul 21];145(1):1. Available from: http://www.embase.com/search/results?subaction=viewrecord&from=export&id=L372038916

29. Rupinski M, Zagorowicz E, Regula J, Fijuth J, Kraszewska E, Polkowski M, et al. Randomized Comparison of Three Palliative Regimens Including Brachytherapy, Photodynamic Therapy and APC in Patients With Malignant Dysphagia (CONSORT 1a) (Revised II). Am J Gastroenterol [Internet]. 2011 Sep 14 [cited 2018 Mar 4];106(9):1612–20. Available from: http://www.nature.com/articles/ajg2011178

30. Kidwell CS, Liebeskind DS, Starkman S, Saver JL. Trends in acute ischemic stroke trials through the 20th century. Stroke [Internet]. 2001 Jun [cited 2017 Nov

9];32(6):1349–59. Available from: http://www.ncbi.nlm.nih.gov/pubmed/11387498

31. Sánchez-Thorin JC, Cortés MC, Montenegro M, Villate N. The quality of reporting of randomized clinical trials published in Ophthalmology. Ophthalmology [Internet]. 2001 Feb [cited 2017 Feb 24];108(2):410–5. Available from: http://www.embase.com/search/results?subaction=viewrecord&from=export&id=L32115976

32. Moher D, Jones A, Lepage L, CONSORT Group (Consolidated Standards for Reporting of Trials). Use of the CONSORT statement and quality of reports of randomized trials: a comparative before-and-after evaluation. JAMA [Internet]. 2001 Apr 18 [cited 2017 Jun 7];285(15):1992–5. Available from: http://www.ncbi.nlm.nih.gov/pubmed/11308436

33. Devereaux PJ, Manns BJ, Ghali WA, Quan H, Guyatt GH. The reporting of methodological factors in randomized controlled trials and the association with a journal policy to promote adherence to the Consolidated Standards of Reporting Trials (CONSORT) checklist. Control Clin Trials [Internet]. 2002 Aug [cited 2017 Feb 24];23(4):380–8. Available from: http://www.embase.com/search/results?subaction=viewrecord&from=export&id=L34874890

34. Montori VM, Bhandari M, Devereaux PJ, Manns BJ, Ghali WA, Guyatt GH. In the dark: the reporting of blinding status in randomized controlled trials. J Clin Epidemiol [Internet]. 2002 Aug [cited 2017 Nov 9];55(8):787–90. Available from: http://www.ncbi.nlm.nih.gov/pubmed/12384193

35. Hill CL, LaValley MP, Felson DT. Secular changes in the quality of published randomized clinical trials in rheumatology. Arthritis Rheum [Internet]. 2002 Mar [cited 2017 Nov 9];46(3):779–84. Available from: http://doi.wiley.com/10.1002/art.512

36. Faunce TA, Buckley NA. Of consents and CONSORTs: reporting ethics, law, and

For peer review only

human rights in RCTs involving monitored overdose of healthy volunteers pre and post the "CONSORT" guidelines. J Toxicol Clin Toxicol [Internet]. 2003 [cited 2017 Nov 9];41(2):93–9. Available from: http://www.ncbi.nlm.nih.gov/pubmed/12733843

37. Halpern SH, Darani R, Douglas MJ, Wight W, Yee J. Compliance with the CONSORT checklist in obstetric anaesthesia randomised controlled trials. Int J Obstet Anesth [Internet]. 2004 Oct [cited 2017 Feb 24];13(4):207–14. Available from: http://www.ncbi.nlm.nih.gov/pubmed/15477048

38. Hewitt C, Hahn S, Torgerson DJ, Watson J, Bland JM. Adequacy and reporting of allocation concealment: review of recent trials published in four general medical journals. BMJ [Internet]. 2005 May 7 [cited 2017 Nov 9];330(7499):1057–8. Available from: http://www.bmj.com/cgi/doi/10.1136/bmj.38413.576713.AE

39. Greenfield MLVH, Rosenberg AL, O'Reilly M, Shanks AM, Sliwinski MJ, Nauss MD. The Quality of Randomized Controlled Trials in Major Anesthesiology Journals. Anesth Analg [Internet]. 2005 Jun [cited 2017 Nov 9];100(6):1759–64. Available from: http://www.ncbi.nlm.nih.gov/pubmed/15920210

40. Llorca J, Martínez-Sanz F, Prieto-Salceda D, Fariñas-Alvarez C, Chinchón MV, Quinones D, et al. Quality of controlled clinical trials on glaucoma and intraocular high pressure. J Glaucoma [Internet]. 2005 Jun [cited 2017 Nov 9];14(3):190–5. Available from: http://www.ncbi.nlm.nih.gov/pubmed/15870599

41. Haahr MT, Hróbjartsson A. Who is blinded in randomized clinical trials? A study of 200 trials and a survey of authors. Clin Trials [Internet]. 2006;3(4):360–5. Available from: http://www.ncbi.nlm.nih.gov/pubmed/17060210

42. Kober T, Trelle S, Engert A. Reporting of randomized controlled trials in Hodgkin lymphoma in biomedical journals. J Natl Cancer Inst [Internet]. 2006 May 3 [cited 2017 Feb 24];98(9):620–5. Available from: http://www.ncbi.nlm.nih.gov/pubmed/16670387

For peer review only

43. Biondi-Zoccai GGL, Lotrionte M, Abbate A, Testa L, Remigi E, Burzotta F, et al. Compliance with QUOROM and quality of reporting of overlapping meta-analyses on the role of acetylcysteine in the prevention of contrast associated nephropathy: case study. BMJ [Internet]. 2006 Jan 28 [cited 2017 Nov 9];332(7535):202–9. Available from: http://www.bmj.com/cgi/doi/10.1136/bmj.38693.516782.7C

44. Balasubramanian SP, Wiener M, Alshameeri Z, Tiruvoipati R, Elbourne D, Reed MW, et al. Standards of reporting of randomized controlled trials in general surgery: Can we do better? Ann Surg [Internet]. 2006 Nov [cited 2017 Feb 24];244(5):663–7. Available from: http://www.ncbi.nlm.nih.gov/pubmed/17060756

45. Dias S, McNamee R, Vail A. Evidence of improving quality of reporting of randomized controlled trials in subfertility. Hum Reprod [Internet]. 2006 Oct [cited 2017 Feb 24];21(10):2617–27. Available from: http://www.ncbi.nlm.nih.gov/pubmed/16793995

46. Smidt N, Rutjes AWS, van der Windt DAWM, Ostelo RWJG, Bossuyt PM, Reitsma JB, et al. The quality of diagnostic accuracy studies since the STARD statement: Has it improved? Neurology [Internet]. 2006 Sep 12 [cited 2018 Mar 12];67(5):792–7. Available from: http://www.ncbi.nlm.nih.gov/pubmed/16966539

47. Coppus SFPJ, van der Veen F, Bossuyt PMM, Mol BWJ. Quality of reporting of test accuracy studies in reproductive medicine: impact of the Standards for Reporting of Diagnostic Accuracy (STARD) initiative. Fertil Steril [Internet]. 2006 Nov [cited 2017 Jun 26];86(5):1321–9. Available from: http://www.embase.com/search/results?subaction=viewrecord&from=export&id=L44602037

48. Tiruvoipati R, Balasubramanian SP, Atturu G, Peek GJ, Elbourne D. Improving the quality of reporting randomized controlled trials in cardiothoracic surgery: the way forward. J Thorac Cardiovasc Surg [Internet]. 2006 Aug [cited 2017 Jul

For peer review only

21];132(2):233–40. Available from: http://www.ncbi.nlm.nih.gov/pubmed/16872940

49. Mahoney J, Ellison J. Assessing the quality of glucose monitor studies: a critical evaluation of published reports. Clin Chem [Internet]. 2007 Jun 19 [cited 2017 Nov 9];53(6):1122–8. Available from: http://www.clinchem.org/cgi/doi/10.1373/clinchem.2006.083493

50. Poolman RW, Abouali JAK, Conter HJ, Bhandari M. Overlapping systematic reviews of anterior cruciate ligament reconstruction comparing hamstring autograft with bone-patellar tendon-bone autograft: why are they different? J Bone Joint Surg Am [Internet]. 2007 Jul 1 [cited 2017 Nov 9];89(7):1542–52. Available from: http://jbjs.org/cgi/doi/10.2106/JBJS.F.01292

51. Spring B, Pagoto S, Knatterud G, Kozak A, Hedeker D. Examination of the analytic quality of behavioral health randomized clinical trials. J Clin Psychol [Internet]. 2007 Jan [cited 2017 Nov 9];63(1):53–71. Available from: http://www.ncbi.nlm.nih.gov/pubmed/17115429

52. Agha R, Cooper D, Muir G. The reporting quality of randomised controlled trials in surgery: A systematic review. Int J Surg [Internet]. 2007 Dec [cited 2017 Nov 9];5(6):413–22. Available from: http://www.ncbi.nlm.nih.gov/pubmed/18029237

53. Kane RL, Wang J, Garrard J. Reporting in randomized clinical trials improved after adoption of the CONSORT statement. J Clin Epidemiol [Internet]. 2007 Mar [cited 2017 Nov 9];60(3):241–9. Available from: http://linkinghub.elsevier.com/retrieve/pii/S0895435606002575

54. Paranjothy B, Shunmugam M, Azuara-Blanco A. The quality of reporting of diagnostic accuracy studies in glaucoma using scanning laser polarimetry. J Glaucoma [Internet]. 2007 Dec [cited 2017 Feb 24];16(8):670–5. Available from: http://www.ncbi.nlm.nih.gov/pubmed/18091453

55. Johnson ZK, Siddiqui MAR, Azuara-Blanco A. The Quality of Reporting of

For peer review only

Diagnostic Accuracy Studies of Optical Coherence Tomography in Glaucoma. Ophthalmology [Internet]. 2007 Sep [cited 2017 Feb 24];114(9):1607–12. Available from: http://www.ncbi.nlm.nih.gov/pubmed/17434589

56. Hind D, Booth A. Do health technology assessments comply with QUOROM diagram guidance? An empirical study. BMC Med Res Methodol [Internet]. 2007 Nov 20 [cited 2017 Jul 17];7:49. Available from: http://www.ncbi.nlm.nih.gov/pubmed/18021461

57. Lee PE, Fischer HD, Rochon PA, Gill SS, Herrmann N, Bell CM, et al. Published randomized controlled trials of drug therapy for dementia often lack complete data on harm. J Clin Epidemiol [Internet]. 2008 Nov [cited 2017 Nov 22];61(11):1152–60. Available from: http://linkinghub.elsevier.com/retrieve/pii/S0895435607004829

58. Pat K, Dooms C, Vansteenkiste J. Systematic review of symptom control and quality of life in studies on chemotherapy for advanced non-small cell lung cancer: How CONSORTed are the data? Lung Cancer [Internet]. 2008 Oct [cited 2017 Mar 10];62(1):126–38. Available from: http://linkinghub.elsevier.com/retrieve/pii/S0169500208001062

59. Folkes A, Urquhart R, Grunfeld E. Are leading medical journals following their own policies on CONSORT reporting? Contemp Clin Trials [Internet]. 2008 Nov [cited 2017 Jun 26];29(6):843–6. Available from: http://linkinghub.elsevier.com/retrieve/pii/S1551714408000906

60. Sinha S, Sinha S, Ashby E, Jayaram R, Grocott MPW. Quality of reporting in randomized trials published in high-quality surgical journals. J Am Coll Surg [Internet]. 2009 Nov [cited 2017 Jul 17];209(5):565–571.e1. Available from: http://linkinghub.elsevier.com/retrieve/pii/S107275150901151X

61. Freeman K, Szczepura A, Osipenko L. Non-invasive fetal RHD genotyping tests: a systematic review of the quality of reporting of diagnostic accuracy in published studies. Eur J Obstet Gynecol Reprod Biol [Internet]. 2009 Feb [cited 2017 Jun

For peer review only

26];142(2):91–8. Available from: http://linkinghub.elsevier.com/retrieve/pii/S0301211508003916

62. Uetani K, Nakayama T, Ikai H, Yonemoto N, Moher D. Quality of reports on randomized controlled trials conducted in Japan: Evaluation of adherence to the CONSORT statement. Intern Med [Internet]. 2009 [cited 2017 Feb 24];48(5):307–13. Available from: http://www.ncbi.nlm.nih.gov/pubmed/19252352

63. Ethgen M, Boutron L, Steg PG, Roy C, Ravaud P. Quality of reporting internal and external validity data from randomized controlled trials evaluating stents for percutaneous coronary intervention. BMC Med Res Methodol [Internet]. 2009 Apr 9 [cited 2017 Feb 24];9(1):24. Available from: http://www.embase.com/search/results?subaction=viewrecord&from=export&id=L354667428

64. Krzych LJ, Liszka L. No improvement in studies reporting the diagnostic accuracy of B-type natriuretic peptide. Med Sci Monit [Internet]. 2009 May [cited 2017 Nov 9];15(5):SR5-14. Available from: http://www.ncbi.nlm.nih.gov/pubmed/19396053

65. Pagoto SL, Kozak AT, John P, Bodenlos JS, Hedeker D, Spring B, et al. Intention-to-treat analyses in behavioral medicine randomized clinical trials. Int J Behav Med [Internet]. 2009 Dec 25 [cited 2017 Feb 24];16(4):316–22. Available from: http://link.springer.com/10.1007/s12529-009-9039-3

66. Han C, Kwak K, Marks DM, Pae C-U, Wu L-T, Bhatia KS, et al. The impact of the CONSORT statement on reporting of randomized clinical trials in psychiatry. Contemp Clin Trials [Internet]. 2009 Mar [cited 2017 Jun 26];30(2):116–22. Available from: http://linkinghub.elsevier.com/retrieve/pii/S1551714408001511

67. Alvarez F, Meyer N, Gourraud PA, Paul C. CONSORT adoption and quality of reporting of randomized controlled trials: a systematic analysis in two dermatology journals. Br J Dermatol [Internet]. 2009 Nov [cited 2017 Feb

For peer review only

23];161(5):1159–65. Available from: http://www.embase.com/search/results?subaction=viewrecord&from=export&id=L355782314

68. Wei X, Tiejun L, Cheng W. Current situation on the reporting quality of randomized controlled trials in 5 leading Chinese medical journals. J Med Coll PLA [Internet]. 2009 Apr [cited 2018 Mar 12];24(2):105–11. Available from: http://linkinghub.elsevier.com/retrieve/pii/S1000194809600253

69. Ladd BO, McCrady BS, Manuel JK, Campbell W. Improving the quality of reporting alcohol outcome studies: Effects of the CONSORT statement. Addict Behav [Internet]. 2010 Jul [cited 2017 Feb 24];35(7):660–6. Available from: http://linkinghub.elsevier.com/retrieve/pii/S0306460310000560

70. Yu L-M, Chan A-W, Hopewell S, Deeks JJ, Altman DG. Reporting on covariate adjustment in randomised controlled trials before and after revision of the 2001 CONSORT statement: a literature review. Trials [Internet]. 2010 May 18 [cited 2017 Nov 9];11(1):59. Available from: http://trialsjournal.biomedcentral.com/articles/10.1186/1745-6215-11-59

71. Areia M, Soares M, Dinis-Ribeiro M. Quality reporting of endoscopic diagnostic studies in gastrointestinal journals: where do we stand on the use of the STARD and CONSORT statements? Endoscopy [Internet]. 2010 Feb 5 [cited 2017 Nov 9];42(2):138–47. Available from: http://www.thieme-connect.de/DOI/DOI?10.1055/s-0029-1243846

72. Delaney M, Meyer E, Cserti-Gazdewich C, Haspel RL, Lin Y, Morris A, et al. A systematic assessment of the quality of reporting for platelet transfusion studies. Transfusion [Internet]. 2010 Oct [cited 2017 Jun 26];50(10):2135–44. Available from: http://doi.wiley.com/10.1111/j.1537-2995.2010.02691.x

73. Flint HE, Harrison JE. How well do reports of clinical trials in the orthodontic literature comply with the CONSORT statement? J Orthod [Internet]. 2010 Dec 16 [cited 2017 Feb 24];37(4):250–61. Available from:

For peer review only

http://www.tandfonline.com/doi/full/10.1179/14653121043191

74. Hopewell S, Dutton S, Yu L-M, Chan A-W, Altman DG. The quality of reports of randomised trials in 2000 and 2006: comparative study of articles indexed in PubMed. BMJ [Internet]. 2010 Mar 23 [cited 2017 Jun 26];340(mar23 1):c723. Available from: http://www.embase.com/search/results?subaction=viewrecord&from=export&id=L358557565

75. Ernst E, Hung SK CY. NCCAM-funded RCTs of herbal medicines: An independent, critical assessment. Perfusion [Internet]. 2011;24:89–102. Available from: https://www.researchgate.net/publication/288438592_NCCAM-funded_RCTs_of_herbal_medicines_An_independent_critical_assessment

76. Selman TJ, Morris RK, Zamora J, Khan KS. The quality of reporting of primary test accuracy studies in obstetrics and gynaecology: application of the STARD criteria. BMC Womens Health [Internet]. 2011 Mar 23 [cited 2017 Jul 17];11(1):8. Available from: http://bmcwomenshealth.biomedcentral.com/articles/10.1186/1472-6874-11-8

77. Parsons NR, Hiskens R, Price CL, Achten J, Costa ML. A systematic survey of the quality of research reporting in general orthopaedic journals. J Bone Jt Surg - Ser B [Internet]. 2011 Sep 1 [cited 2017 Feb 24];93 B(9):1154–9. Available from: http://www.bjj.boneandjoint.org.uk/cgi/doi/10.1302/0301-620X.93B9.27193

78. Kiehna EN, Starke RM, Pouratian N, Dumont AS. Standards for reporting randomized controlled trials in neurosurgery. J Neurosurg [Internet]. 2011 Feb [cited 2017 Feb 24];114(2):280–5. Available from: http://thejns.org/doi/10.3171/2010.8.JNS091770

79. Strech D, Soltmann B, Weikert B, Bauer M, Pfennig A. Quality of reporting of randomized controlled trials of pharmacologic treatment of bipolar disorders: A systematic review. J Clin Psychiatry [Internet]. 2011 Sep 15 [cited 2017 Feb 24];72(9):1214–21. Available from:

For peer review only

http://article.psychiatrist.com/?ContentType=START&ID=10007267

80. Turner L-A, Singh K, Garritty C, Tsertsvadze A, Manheimer E, Wieland LS, et al. An evaluation of the completeness of safety reporting in reports of complementary and alternative medicine trials. BMC Complement Altern Med [Internet]. 2011 Aug 22 [cited 2017 Jun 26];11(1):67. Available from: http://bmccomplementalternmed.biomedcentral.com/articles/10.1186/1472-6882-11-67

81. Haidich A-B, Birtsou C, Dardavessis T, Tirodimos I, Arvanitidou M. The quality of safety reporting in trials is still suboptimal: survey of major general medical journals. J Clin Epidemiol [Internet]. 2011 Feb [cited 2017 Feb 24];64(2):124–35. Available from: http://linkinghub.elsevier.com/retrieve/pii/S0895435610001083

82. Gray R, Sullivan M, Altman DG, Gordon-Weeks AN. Adherence of trials of operative intervention to the CONSORT statement extension for nonpharmacological treatments: A comparative before and after study. Ann R Coll Surg Engl [Internet]. 2012 Sep 1 [cited 2017 Feb 24];94(6):388–94. Available from: http://openurl.ingenta.com/content/xref?genre=article&issn=0035-8843&volume=94&issue=6&spage=388

83. Cornelius VR, Sauzet O, Williams JE, Ayis S, Farquhar-Smith P, Ross JR, et al. Adverse event reporting in randomised controlled trials of neuropathic pain: considerations for future practice. Pain [Internet]. 2013 Feb [cited 2017 Nov 22];154(2):213–20. Available from: http://content.wkhealth.com/linkback/openurl?sid=WKPTLP:landingpage&an=00006396-201302000-00009

84. Diaz-Ordaz K, Froud R, Sheehan B, Eldridge S. A systematic review of cluster randomised trials in residential facilities for older people suggests how to improve quality. BMC Med Res Methodol [Internet]. 2013 Oct 22 [cited 2017 Jul 17];13(1):127. Available from: http://bmcmedresmethodol.biomedcentral.com/articles/10.1186/1471-2288-13-127

For peer review only

85. Geha NN, Moseley AM, Elkins MR, Chiavegato LD, Shiwa SR, Costa LOP. The quality and reporting of randomized trials in cardiothoracic physical therapy could be substantially improved. Respir Care [Internet]. 2013 Nov;58(11):1899–906. Available from: http://rc.rcjournal.com/cgi/doi/10.4187/respcare.02379

86. Liu LQ, Morris PJ, Pengel LHM. Compliance to the CONSORT statement of randomized controlled trials in solid organ transplantation: A 3-year overview. Transpl Int [Internet]. 2013 Mar [cited 2017 Feb 24];26(3):300–6. Available from: http://www.embase.com/search/results?subaction=viewrecord&from=export&id=L52379666

87. Panic N, Leoncini E, de Belvis G, Ricciardi W, Boccia S. Evaluation of the endorsement of the preferred reporting items for systematic reviews and meta-analysis (PRISMA) statement on the quality of published systematic review and meta-analyses. Derrick GE, editor. PLoS One [Internet]. 2013 Dec 26 [cited 2017 Jan 15];8(12):e83138. Available from: http://www.embase.com/search/results?subaction=viewrecord&from=export&id=L372005920

88. Fleming PS, Seehra J, Polychronopoulou A, Fedorowicz Z, Pandis N. A PRISMA assessment of the reporting quality of systematic reviews in orthodontics. Angle Orthod [Internet]. 2013 Jan [cited 2017 Nov 9];83(1):158–63. Available from: http://www.ncbi.nlm.nih.gov/pubmed/22720835

89. Péron J, Maillet D, Gan HK, Chen EX, You B. Adherence to CONSORT adverse event reporting guidelines in randomized clinical trials evaluating systemic cancer therapy: A systematic review. J Clin Oncol [Internet]. 2013 Nov 1 [cited 2017 Feb 24];31(31):3957–63. Available from: http://ascopubs.org/doi/10.1200/JCO.2013.49.3981

90. Tunis AS, McInnes MDF, Hanna R, Esmail K. Association of Study Quality with Completeness of Reporting: Have Completeness of Reporting and Quality of Systematic Reviews and Meta-Analyses in Major Radiology Journals Changed

For peer review only

Since Publication of the PRISMA Statement? Radiology [Internet]. 2013 Nov [cited 2018 Mar 12];269(2):413–26. Available from: http://pubs.rsna.org/doi/10.1148/radiol.13130273

91. Maclean EN, Stone IS, Ceelen F, Garcia-Albeniz X, Sommer WH, Petersen SE. Quality of reporting in cardiac MRI, CT and SPECT diagnostic accuracy studies: Analysis of the impact of STARD criteria. J Cardiovasc Magn Reson [Internet]. 2013 Jun 1 [cited 2017 Feb 24];15(6):61. Available from: http://www.embase.com/search/results?subaction=viewrecord&from=export&id=L70993941

92. Baker D, Lidster K, Sottomayor A, Amor S. Two Years Later: Journals Are Not Yet Enforcing the ARRIVE Guidelines on Reporting Standards for Pre-Clinical Animal Studies. Eisen JA, editor. PLoS Biol [Internet]. 2014 Jan 7 [cited 2017 Sep 29];12(1):e1001756. Available from: http://www.ncbi.nlm.nih.gov/pubmed/24409096

93. Choi J, Jun JH, Kang BK, Kim KH, Lee MS. Endorsement for improving the quality of reports on randomized controlled trials of traditional medicine journals in Korea: a systematic review. Trials [Internet]. 2014 Nov 5 [cited 2017 Jan 15];15(1):429. Available from: http://trialsjournal.biomedcentral.com/articles/10.1186/1745-6215-15-429

94. Walther S, Schueler S, Tackmann R, Schuetz GM, Schlattmann P, Dewey M, et al. Compliance with STARD checklist among studies of coronary CT angiography: systematic review. Radiology [Internet]. 2014 Apr [cited 2017 Feb 23];271(1):74–86. Available from: http://pubs.rsna.org/doi/10.1148/radiol.13121720

95. Ghimire S, Kyung E, Lee H, Kim E. Oncology trial abstracts showed suboptimal improvement in reporting: a comparative before-and-after evaluation using CONSORT for Abstract guidelines. J Clin Epidemiol [Internet]. 2014 Jun [cited 2017 Feb 24];67(6):658–66. Available from: http://linkinghub.elsevier.com/retrieve/pii/S0895435613004435

For peer review only

96. Song T-J, Leng H-F, Zhong LL, Wu T-X, Bian Z-X. CONSORT in China: past development and future direction. Trials [Internet]. 2015 Jun 1 [cited 2017 Jun 26];16(1):243. Available from: http://trialsjournal.biomedcentral.com/articles/10.1186/s13063-015-0769-z

97. Stevely A, Dimairo M, Todd S, Julious SA, Nicholl J, Hind D, et al. An Investigation of the Shortcomings of the CONSORT 2010 Statement for the Reporting of Group Sequential Randomised Controlled Trials: A Methodological Systematic Review. Shamji M, editor. PLoS One [Internet]. 2015 Nov 3 [cited 2017 Feb 24];10(11):e0141104. Available from: http://dx.plos.org/10.1371/journal.pone.0141104

98. Adie S, Ma D, Harris IA, Naylor JM, Craig JC. Quality of conduct and reporting of meta-analyses of surgical interventions. Ann Surg [Internet]. 2015 Apr [cited 2017 Jun 26];261(4):685–94. Available from: http://content.wkhealth.com/linkback/openurl?sid=WKPTLP:landingpage%7B&%7Dan=00000658-201504000-00012

99. Jull A, Aye PS. Endorsement of the CONSORT guidelines, trial registration, and the quality of reporting randomised controlled trials in leading nursing journals: A cross-sectional analysis. Int J Nurs Stud [Internet]. 2015 Jun [cited 2017 Feb 10];52(6):1071–9. Available from: http://linkinghub.elsevier.com/retrieve/pii/S0020748914003022

100. Bearn DR, Alharbi F. Reporting of clinical trials in the orthodontic literature from 2008 to 2012: observational study of published reports in four major journals. J Orthod [Internet]. 2015 Sep 15 [cited 2017 Feb 24];42(3):186–91. Available from: http://www.tandfonline.com/doi/full/10.1179/1465313315Y.0000000011

101. Agha RA, Fowler AJ, Limb C, Whitehurst K, Coe R, Sagoo H, et al. Impact of the mandatory implementation of reporting guidelines on reporting quality in a surgical journal: A before and after study. Int J Surg [Internet]. 2016 Jun [cited 2017 Feb 20];30:169–72. Available from: http://linkinghub.elsevier.com/retrieve/pii/S1743919116300620

For peer review only

102. Pouwels KB, Widyakusuma NN, Groenwold RHH, Hak E. Quality of reporting of confounding remained suboptimal after the STROBE guideline. J Clin Epidemiol [Internet]. 2016 Jan [cited 2017 Feb 24];69:217–24. Available from: http://linkinghub.elsevier.com/retrieve/pii/S0895435615003856

103. Rao A, Brück K, Methven S, Evans R, Stel VS, Jager KJ, et al. Quality of reporting and study design of CKD cohort studies assessing mortality in the elderly before and after STROBE: A systematic review. Zhou X, editor. PLoS One [Internet]. 2016 May 11 [cited 2017 Jun 26];11(5):e0155078. Available from: http://dx.plos.org/10.1371/journal.pone.0155078

104. Grob ATM, van der Vaart LR, Withagen MIJ, van der Vaart CH. The quality of reporting of diagnostic accuracy studies in pelvic floor transperineal three-dimensional ultrasound: a systematic review. Ultrasound Obstet Gynecol [Internet]. 2016 Dec 21 [cited 2017 Mar 10]; Available from: http://doi.wiley.com/10.1002/uog.17390

105. Rikos D, Dardiotis E, Tsivgoulis G, Zintzaras E, Hadjigeorgiou GM. Reporting quality of randomized-controlled trials in multiple sclerosis from 2000 to 2015, based on CONSORT statement. Mult Scler Relat Disord [Internet]. 2016 Sep [cited 2017 Feb 24];9:135–9. Available from: http://linkinghub.elsevier.com/retrieve/pii/S2211034816301225

106. Bigna JJR, Um LN, Nansseu JRN. A comparison of quality of abstracts of systematic reviews including meta-analysis of randomized controlled trials in high-impact general medicine journals before and after the publication of PRISMA extension for abstracts: A systematic review and m. Syst Rev [Internet]. 2016 Oct 13 [cited 2017 Jun 26];5(1):174. Available from: http://www.embase.com/search/results?subaction=viewrecord&from=export&id=L612833923

107. Sarkis-Onofre R, Poletto-Neto V, Cenci MS, Pereira-Cenci T, Moher D. Impact of the CONSORT Statement endorsement in the completeness of reporting of randomized clinical trials in restorative dentistry. J Dent [Internet]. 2017 Mar 31

For peer review only

[cited 2017 Feb 24];58:54–9. Available from: http://linkinghub.elsevier.com/retrieve/pii/S0300571217300283

108. Tharyan P, Premkumar TS, Mathew V, Barnabas JP, Manuelraj. Editorial policy and the reporting of randomized controlled trials: a survey of instructions for authors and assessment of trial reports in Indian medical journals (2004-05). Natl Med J India [Internet]. [cited 2017 Nov 9];21(2):62–8. Available from: http://www.ncbi.nlm.nih.gov/pubmed/18807310

109. Lai TYY, Wong VWY, Lam RF, Cheng ACO, Lam DSC, Leung GM. Quality of reporting of key methodological items of randomized controlled trials in clinical ophthalmic journals. Ophthalmic Epidemiol [Internet]. 14(6):390–8. Available from: http://www.ncbi.nlm.nih.gov/pubmed/18161613

110. Jilka RL. The Road to Reproducibility in Animal Research. J Bone Miner Res [Internet]. 2016 Jul [cited 2017 Jul 21];31(7):1317–9. Available from: http://doi.wiley.com/10.1002/jbmr.2881

111. Moher D, Altman DG, Roberts I, Dirnagl U, Chalmers I, Berlin J. Four Proposals to Help Improve the Medical Research Literature. PLOS Med [Internet]. 2015 Sep 22 [cited 2017 Aug 29];12(9):e1001864. Available from: http://dx.plos.org/10.1371/journal.pmed.1001864

112. Hutton B, Salanti G, Caldwell DM, Chaimani A, Schmid CH, Cameron C, et al. The PRISMA extension statement for reporting of systematic reviews incorporating network meta-analyses of health care interventions: checklist and explanations. Ann Intern Med [Internet]. 2015 Jun 2 [cited 2017 Jun 26];162(11):777–84. Available from: http://annals.org/article.aspx?doi=10.7326/M14-2385

113. Cobo E, Selva-O'Callagham A, Ribera JM, Cardellach F, Dominguez R, Vilardell M. Statistical reviewers improve reporting in biomedical articles: A randomized trial. PLoS One. 2007;2(3).

114. Hirst A, Altman DG. Are peer reviewers encouraged to use reporting guidelines? A survey of 116 health research journals. Cameron DW, editor. PLoS One

For peer review only

[Internet]. 2012 Apr 27 [cited 2017 Jun 7];7(4):e35621. Available from: http://dx.plos.org/10.1371/journal.pone.0035621

115. Pandis N, Shamseer L, Kokich VG, Fleming PS, Moher D. Active implementation strategy of CONSORT adherence by a dental specialty journal improved randomized clinical trial reporting. J Clin Epidemiol [Internet]. 2014 Sep [cited 2017 Jan 15];67(9):1044–8. Available from: http://linkinghub.elsevier.com/retrieve/pii/S0895435614001140

116. Cobo E, Cortes J, Ribera JM, Cardellach F, Selva-O'Callaghan A, Kostov B, et al. Effect of using reporting guidelines during peer review on quality of final manuscripts submitted to a biomedical journal: masked randomised trial. 2011 Nov;343(nov22 2). Available from: http://www.ncbi.nlm.nih.gov/pubmed/22108262

117. Hopewell S, Collins GS, Boutron I, Yu L-M, Cook J, Shanyinde M, et al. Impact of peer review on reports of randomised trials published in open peer review journals: retrospective before and after study. BMJ [Internet]. 2014 [cited 2017 Jun 7];349. Available from: http://www.bmj.com/content/349/bmj.g4145

118. Statreviewer. Available from: http://www.statreviewer.com/

119. A peerless review? Automating methodological and statistical review. Available from: https://blogs.biomedcentral.com/bmcblog/2016/05/23/peerless-review-automating-methodological-statistical-review/

120. Tools and templates for implementing reporting guidelines. Available from: http://www.equator-network.org/toolkits/using-guidelines-in-journals/tools-and-templates-for-implementing-reporting-guidelines/

121. Hopewell S, Boutron I, Altman DG, Barbour G, Moher D, Montori V, et al. Impact of a web-based tool (WebCONSORT) to improve the reporting of randomised trials: results of a randomised controlled trial. BMC Med [Internet]. 2016 Nov 28 [cited 2017 Feb 24];14(1):199. Available from: http://www.ncbi.nlm.nih.gov/pubmed/27894295

For peer review only

122. Mbuagbaw L, Thabane M, Vanniyasingam T, Borg Debono V, Kosa S, Zhang S, et al. Improvement in the quality of abstracts in major clinical journals since CONSORT extension for abstracts: a systematic review. Contemp Clin Trials [Internet]. 2014 Jul [cited 2017 Feb 24];38(2):245–50. Available from: http://linkinghub.elsevier.com/retrieve/pii/S1551714414000809

123. PubPeer. Available from: https://pubpeer.com/

124. ScienceOpen. Available from: https://www.scienceopen.com/

125. Schriger DL, Altman DG. Inadequate post-publication review of medical research. BMJ [Internet]. 2010 Aug 11 [cited 2018 Mar 4];341:c3803. Available from: http://www.ncbi.nlm.nih.gov/pubmed/20702543

126. Pandis N, Polychronopoulou A, Eliades T. An assessment of quality characteristics of randomised control trials published in dental journals. J Dent [Internet]. 2010 Sep [cited 2017 Feb 24];38(9):713–21. Available from: http://linkinghub.elsevier.com/retrieve/pii/S0300571210001235

127. Péron J, You B, Gan HK, Maillet D, Chen EX, Pond GR, et al. Influence of statistician involvement on reporting of randomized clinical trials in medical oncology. Anticancer Drugs [Internet]. 2013 Mar [cited 2017 Feb 24];24(3):306–9. Available from: http://content.wkhealth.com/linkback/openurl?sid=WKPTLP:landingpage&an=00001813-201303000-00010

128. Kloukos D, Papageorgiou SN, Doulis I, Petridis H, Pandis N. Reporting quality of randomised controlled trials published in prosthodontic and implantology journals. J Oral Rehabil [Internet]. 2015 Dec [cited 2017 Feb 24];42(12):914–25. Available from: http://doi.wiley.com/10.1111/joor.12325

129. Blanco D, Biggane AM, Cobo E. Are CONSORT checklists submitted by authors adequately reflecting what information is actually reported in published papers? Trials [Internet]. 2018 Dec 29 [cited 2018 Jan 31];19(1):80. Available from: https://trialsjournal.biomedcentral.com/articles/10.1186/s13063-018-2475-0

For peer review only

130. Koletsi D, Fleming PS, Behrents RG, Lynch CD, Pandis N. The use of tailored subheadings was successful in enhancing compliance with CONSORT in a dental journal. J Dent [Internet]. 2017 Dec [cited 2018 Mar 23];67:66–71. Available from: http://www.ncbi.nlm.nih.gov/pubmed/28941813

131. Cobo E, González JA. Taking advantage of unexpected WebCONSORT results. BMC Med [Internet]. 2016 Dec 5 [cited 2018 Feb 16];14(1):204. Available from: http://bmcmedicine.biomedcentral.com/articles/10.1186/s12916-016-0758-4

132. Tijdink JK, Schipper K, Bouter LM, Maclaine Pont P, de Jonge J, Smulders YM. How do scientists perceive the current publication culture? A qualitative focus group interview study among Dutch biomedical researchers. BMJ Open [Internet]. 2016 Feb 17 [cited 2017 Sep 28];6(2):e008681. Available from: http://bmjopen.bmj.com/lookup/doi/10.1136/bmjopen-2015-008681

Figures, tables and supplementary files

Figure 1: PRISMA flow diagram

Figure 2: Typology of interventions to improve adherence to RGs according to type of intervention and research stage. **Legend:** Evaluated interventions are shown in bold.

Figure 3: Gaps in research on the evaluation of interventions to improve adherence to reporting guidelines. **Legend:** Each circle represents one intervention. Variables displayed: 1) Circle size: Number of studies evaluating each intervention (bigger = more studies); 2) Circle colour: Study design of those studies (blue for RCTs and green for observational studies) and 3) Circle fill: Kind of RG implementation (plain for checklist and stripes for bullet points and examples). Research gaps are highlighted in red.

Table 1: Rationale of the interventions identified.

Supplementary file 1: Description of the acronyms and full names of the reporting guidelines shown in the EQUATOR website as "Reporting Guidelines for main study types" on 8 May 2017.

For peer review only

Supplementary file 2: Search terms for PubMed (from January 1, 1996, to March 31, 2017) via PubMed.

Supplementary file 3: implementation details of the evaluated interventions.

For peer review only

Group	Intervention	Rationale
**Training**	Introduction of RGs & journalology into graduate curricula (17-21)	To introduce good research reporting habits early in young researchers' scientific careers.
	Student's development of protocols for coursework and research using RGs (20)	
	Funder's support of author training on RGs (1)	Authors, editors, and peer reviewers have insufficient training in issues related to reporting.
	Training for peer reviewers and editors on RGs by journals (1,21)	
**Improved understanding**	Translation of RGs to further languages (22)	Language barriers may affect the proper use of RGs.
	Development of expanded database of examples for each RG (23)	Authors need more examples of good reporting to properly understand certain items.
**Encouraging adherence**	Dissemination of RGs by scientific associations (24)	A large number of researchers are not aware of the existence of RGs.
	Author use of RGs as a template for grant application proposals (20)	Using RGs in early stages may facilitate completeness of reporting of published research.
	Required checklist for ethics approval application (11)	
	Funder's requirement of checklists in author's report (20,25)	

only

	Author use of the writing aid tool COBWEB (12)	A) Authors need help to successfully adhere to RGs at the writing stage and B) Dividing RG items into bullet points and providing examples might help.
	Author use of a structured approach for reporting research (27)	A) To help authors avoid omissions, B) to aid reviewers and editors in appraising articles and C) to allow more efficient data extraction during the systematic review process.
	Author markup of the manuscript to indicate where each RG item is addressed (29)	
	Editorial statement endorsing certain RGs (30-109)	Authors read editorial statements and follow “Instructions to authors”.
	Recommendation or requirement to follow RGs in the "Instructions to authors“ (30-109)	
	Requirement to submit a RG checklist together with the manuscript indicating page numbers corresponding to each item (30-109)	Authors may not consider editorial statements or recommendations in “Instructions to authors” to be important. Compulsory submission of checklists or text mark-up may encourage authors to be more compliant with RGs.
	Requirement to populate and submit a RG checklist with text from the manuscript (112)	

only

For peer review only - http://bmjopen.bmj.com/site/about/guidelines.xhtml

	Journal development of core versions of RGs containing key items (110)	Focusing on the most important items could be more effective than considering the whole checklist.
	Guidance to authors on manuscript preparation by publication officers (111)	Trained journal officers may enhance authors' compliance with RGs during manuscript preparation.
	Suggestion for peer reviewers to use RGs (113)	Peer reviewers often do not detect reporting flaws. Therefore, they may need to follow a more systematic approach and use RGs.
	Editor's questions to peer reviewers about whether the authors have followed RGs (114)	
**Monitoring adherence and providing feedback**	Completeness of reporting check by editors (115)	Requiring checklists at submission does not guarantee adherence. Editors and peer reviewers have to check whether submitted papers are compliant with RGs.
	Peer review against RGs (116)	
	Internal peer review against RGs by a trained editorial assistant (117)	It is extremely unlikely that the average clinical peer reviewer has the methodological expertise to check a paper against RGs.
	Implementation of the automatic tool Statreviewer (119)	
	Email to authors to revise the manuscript according to RGs (13)	It might be more effective to ask authors for adherence to RGs during the revision process

only

	Implementation of the tool WebCONSORT (121)	because they will do anything to get their paper published.
	Completeness of reporting check at copy-editing (122)	Copy-editing and post-publication offer alternate time points to improve adherence to RGs.
	Post- publication peer review (125)	
**Collaboration among authors and experts**	Statistician involvement (84,126-128)	Professionals with specific knowledge of RGs might help authors when designing, conducting or reporting their research.
	Medical writer involvement (25)	

For peer review only - http://bmjopen.bmj.com/site/about/guidelines.xhtml

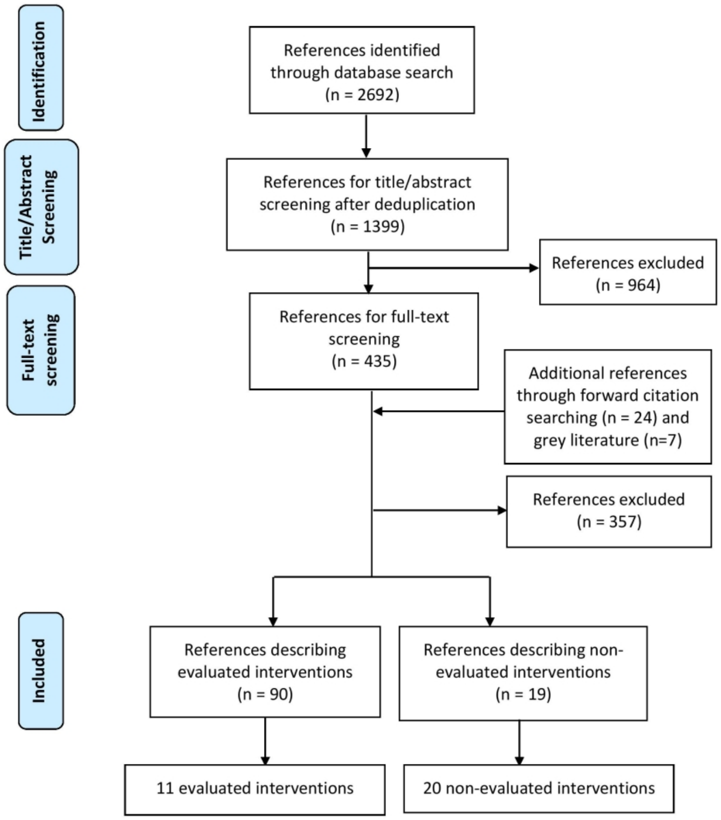

215x279mm (300 x 300 DPI)

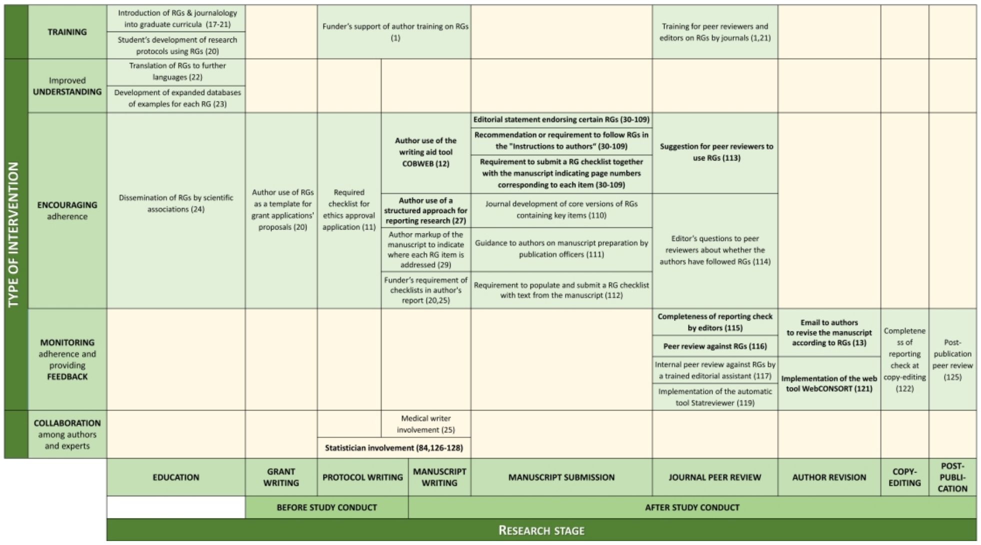

670x379mm (300 x 300 DPI)

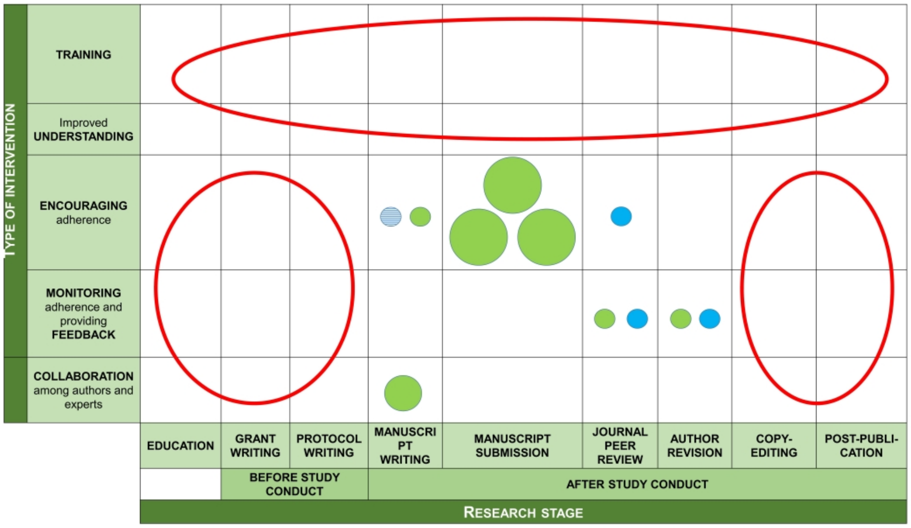

670x379mm (300 x 300 DPI)

Acronym	Full name
CONSORT	Consolidated Standards of Reporting Trials
STROBE	Strengthening the Reporting of Observational Studies in Epidemiology
PRISMA	Preferred Reporting Items for Systematic Reviews and Meta-Analyses
SRQR	Standards for Reporting Qualitative Research
COREQ	Consolidated criteria for Reporting Qualitative research
STARD	Standard Protocol Items: Recommendations for Interventional Trials
TRIPOD	Transparent Reporting of a multivariable prediction model for Individual Prognosis or Diagnosis
SQUIRE	Standards for Quality Improvement Reporting Excellence
CHEERS	Consolidated Health Economic Evaluation Reporting Standards
SPIRIT	Standard Protocol Items: Recommendations for Interventional Trials
PRISMA-P	Preferred Reporting Items for Systematic Review and Meta-Analysis Protocols
CARE	Case Report
AGREE	Appraisal of Guidelines, Research and Evaluation
ARRIVE	Animal Research: Reporting In Vivo Experiments
RIGHT	Reporting Tool for Practice Guidelines in Health Care

For peer review only

Steps	Search terms
S1	impact* [tw]
S2	improv* [tw]
S3	enhanc* [tw]
S4	boost* [tw]
S5	increas* [tw]
S6	influenc* [tw]
S7	effect [tw]
S8	S1 **OR** S2 **OR** S3 **OR** S4 **OR** S5 **OR** S6 **OR** S7
S9	compliance [tw]
S10	adherence [tw]
S11	completeness [tw]
S12	quality of reporting [tw]
S13	reporting quality [tw]
S14	S9 **OR** S10 **OR** S11 **OR** S12 **OR** S 13
S15	Consolidated [tw] Standards [tw] Reporting [tw] Trials [tw] **OR** CONSORT[tw]
S16	Strengthening [tw] Reporting [tw] Observational [tw] Studies [tw] Epidemiology[tw] **OR** STROBE[tw]
S17	Preferred [tw] Reporting [tw] Items [tw] Systematic [tw] reviews [tw] Meta-Analyses [tw] **OR** PRISMA[tw]
S18	Standards [tw] Reporting [tw] Qualitative Research[tw] **OR** SRQR[tw]
S19	Consolidated [tw] Criteria [tw] Reporting [tw] Qualitative [tw] Research[tw] **OR** COREQ[tw]
S20	Standard [tw] Protocol [tw] Items [tw] Recommendations [tw] Interventional [tw] Trials[tw] **OR** STARD[tw]
S21	Transparent [tw] Reporting [tw] multivariable [tw] prediction [tw] model [tw] Individual [tw] Prognosis [tw] Diagnosis[tw] **OR** TRIPOD[tw]
S22	Standards [tw] QUality [tw] Improvement [tw] Reporting [tw] Excellence[tw] **OR** SQUIRE[tw]
S23	Consolidated [tw] Health [tw] Economic [tw] Evaluation [tw] Reporting [tw] Standards[tw] **OR** CHEERS[tw]
S24	Standard [tw] Protocol [tw] Items [tw] Recommendations [tw] Interventional [tw] Trials[tw] **OR** SPIRIT[tw]
S25	Preferred [tw] Reporting [tw] Items [tw] Systematic [tw] Review [tw] Meta-Analysis [tw] Protocols[tw] **OR** PRISMA-P[tw]
S26	Quality [tw] Reporting [tw] Meta-analyses[tw] **OR** QUOROM[tw]
S27	Case [tw] Report [tw] **AND** CARE[tw]
S28	Appraisal [tw] Guidelines [tw] Research [tw] Evaluation[tw] **AND** AGREE[tw]
S29	Animal [tw] Research [tw] Reporting [tw] Vivo [tw] Experiments[tw] **AND** ARRIVE[tw]
S30	Reporting [tw] Tool [tw] Practice [tw] Guidelines [tw] Health [tw] Care[tw] **AND** RIGHT[tw]
S31	S15 **OR** S16 **OR** S17 **OR** S18 **OR** S19 **OR** S20 **OR** S21 **OR** S22 **OR** S23 **OR** S24 **OR** S25 **OR** S26 **OR** S27 **OR** S28 **OR** S29 **OR** S30
S32	S8 **AND** S14 **AND** S31
S33	S32 **AND** "1996/01/01"[PDAT] : "2017/03/31"[PDAT]

For peer review only

For peer review only

Type of intervention	Intervention	Number of studies and study design	Details of the intervention	RGs implemented	Format of RG implementation	Measure of adherence to RGs	Effect on adherence to RGs
Encouraged adherence	Implementation of the writing aid tool COBWEB (12)	1 RCT	Participants have to write the six domains of the methods section of the manuscript for the protocol they receive. They have access to COBWEB tool for a random three of the six domains.	CONSORT & CONSORT extension for non-pharmacological interventions	Bullet points and examples (6 items)	Mean global score for completeness of reporting (scale 0–10, items weighted).	Significant: increase of 2.1 (95% CI 1.5-2.7)
	Author use of a structured approach for reporting research (27)	1 Observational study (cross-sectional evaluation)	Results are posted in a standard tabular format without discussions or conclusions.	CONSORT	Checklist (4 items)	Percentage compliance of each RG item	Significant: all 4 items studied improved significantly
	Journal endorsement (3 interventions, see “Details of the intervention”) (30-109)	80 observational studies (57 cross sectional evaluations of endorsing vs non-endorsing journals, 9 before and after evaluations of endorsing journals before and after endorsement, 14 both kind of evaluations)	A) Editorial statement endorsing certain RGs,<br>B) Recommendation or requirement to follow RGs in the "Instructions to authors", and<br>C) Requirement to submit a RG checklist together with the manuscript indicating page numbers corresponding to each item.	CONSORT (46 of 80)<br>CONSORT extensions (9 of 80)<br>QUOROM (3 of 80)<br>PRISMA (4 of 80)<br>PRISMA extensions (1 of 80)<br>STARD (11 of 80)	Checklist (all items)	Relative proportions of studies adequately reporting any of the item & percentage compliance of each RG item	Significant for CONSORT: 25 items improved, five significantly<br><br>Not significant for other RGs**

				STROBE (4 of 80) ARRIVE (1 of 80) CONSORT, STROBE and PRISMA (11 of 80)			
	Suggestion for peer reviewers to use RGs (113)	1 RCT	Peer reviewers are sent a standard letter encouraging them to use different reporting guidelines. Reviewers are not asked to report whether they used the reporting guideline in reviewing the manuscript.	CONSORT, QUOROM, STARD	Checklist (all items)	Modified version of Manuscript Quality Assessment Instrument (scale 36-180)	Not significant: increase of 0.9 (95% CI -0.3 to +2.1)
Monitoring adherence and providing feedback	Completeness of reporting check by the editors (115)	1 Observational Study (Before and after evaluation)	Initial submissions are vetted by the editor-in-chief. If the submission is considered appropriate, manuscripts are assessed by the associate editor for CONSORT adherence. Authors are asked to make changes accordingly until associate editor deems appropriate that they move to the next step of the review process leading to an editorial decision.	CONSORT	Checklist (all items)	Percentage compliance of each RG item	Significant: Before – compliance ranged from 0% to 100% (Median 40%) / After – perfect compliance in 33 out of 37 items

	Additional review against RGs (116)	1 RCT	A senior statistician does an additional review of all papers and provides authors suggestions on how to follow reporting guideline checklists.	STROBE, CONSORT, STARD	Checklist (all items)	Modified version of Manuscript Quality Assessment Instrument (scale 1 to 9)	Not significant: increase of 0.25 (95% CI -0.05 to +0.54)
	Active implementation of RG by editors (2 interventions, see "Details of the intervention") (13)	1 Observational study (Interrupted time series evaluation)	A) Email is sent to authors to revise the abstract according to the guidelines at the revision stage and B) Changes are made by the assistant editors of these journals towards the end of the editorial process.	CONSORT extension for abstracts	Checklist (9 of 17 items)	Monthly mean number of items reported	Significant: increase 1.50 items
	Implementation of the web-based tool WebCONSORT (121)	1 RCT	Journal editor includes a link to WebCONSORT in the revision letter to authors. Authors are directed to an automatically generated list of items and a flow diagram customised to their specific trial design.	CONSORT & some CONSORT extensions	Checklist (10 of 25 items)	Percentage of possible items reported for each article	Not significant: increase of 0.04 (95% CI −0.02 to +0.10)
Collaboration among authors and experts	Statistician involvement (84,126-128)	4 Observational studies (cross sectional evaluations)	Statisticians (or epidemiologists or other quantitative methodologists) are involved in the design, conduct or reporting of the study	CONSORT	Checklist (all items)	Percentage of possible items reported for each article	Significant in three studies (84,126,128), not significant in one (127)

*According to the systematic review by Turner et al. (6) **According to the systematic review by Stevens et al. (3)

BMJ Open

A scoping review on interventions to improve adherence to reporting guidelines in health research

Journal:	*BMJ Open*
Manuscript ID	bmjopen-2018-026589.R1
Article Type:	Research
Date Submitted by the Author:	20-Nov-2018
Complete List of Authors:	Blanco, David; Universitat Politècnica de Catalunya, Statistics and Operations Research<br>Altman, Doug; Centre for Statistics in Medicine<br>Moher, David; Ottawa Hospital Research Institute, Centre for Journalology<br>Boutron, Isabelle; Université Paris Descartes, Centre d\'épidémiologie clinique<br>Kirkham, Jamie; University of Liverpool, Biostatistics<br>Cobo, Erik; Universitat Politècnica de Catalunya, Statistics and Operations Research
**Primary Subject Heading**:	Medical publishing and peer review
Secondary Subject Heading:	Medical education and training, Research methods
Keywords:	Scoping review, Quality of reporting, Completeness of reporting, Reporting guidelines, Knowledge synthesis, Adherence

SCHOLARONE™
Manuscripts

A scoping review on interventions to improve adherence to reporting guidelines in health research

Authors

David Blanco[1,2], Doug Altman[3], David Moher[4], Isabelle Boutron[5], Jamie J Kirkham[6*] and Erik Cobo[1*].

*JJK and EC are joint senior authors.

[1]Statistics and Operations Research Department, Universitat Politècnica de Catalunya - Barcelona Tech, Barcelona, Spain.

[2]INSERM, U1153 Epidemiology and Biostatistics Sorbonne Paris Cité Research Center (CRESS), Methods of Therapeutic Evaluation of Chronic Diseases (METHODS) Team, Paris Descartes University, Sorbonne Paris Cité, Paris F-75014, France.

[3]Centre for Statistics in Medicine, University of Oxford, Oxford, UK.

[4]Centre for Journalology, Ottawa Hospital Research Institute, Ottawa, Canada.

[5]Centre d'Epidémiologie Clinique, Université Paris Descartes, Paris, France.

[6]Department of Biostatistics, University of Liverpool, Liverpool, UK.

Correspondence to David Blanco; david.blanco.tena@upc.edu

Keywords

Scoping review, knowledge synthesis, reporting guidelines, completeness of reporting, quality of reporting, adherence

Abstract

Objectives: The goal of this study is to identify, analyse and classify interventions to improve adherence to reporting guidelines in order to obtain a wide picture of how the

For peer review only

problem of enhancing the completeness of reporting of biomedical literature has been tackled so far.

Design: Scoping review.

Search strategy: We searched the MEDLINE, EMBASE, and Cochrane Library databases and conducted a grey literature search for (i) studies evaluating interventions to improve adherence to reporting guidelines in health research and (ii) other types of references describing interventions that have been performed or suggested but never evaluated. The characteristics and effect of the evaluated interventions were analysed. Moreover, we explored the rationale of the interventions identified and determined the existing gaps in research on the evaluation of interventions to improve adherence to reporting guidelines.

Results: 109 references containing 31 interventions (11 evaluated) were included. These were grouped into five categories: (1) training on the use of reporting guidelines, (2) improving understanding, (3) encouraging adherence, (4) checking adherence and providing feedback, and (5) involvement of experts. Research gaps identified included the evaluation of interventions (i) on training on the use of reporting guidelines and improving understanding of these, (ii) at early stages of research, and (iii) after the final acceptance of the manuscript.

Conclusions: This scoping review identifies a wide range of strategies to improve adherence to reporting guidelines that can be taken by different stakeholders. Future randomised trials should consider evaluating some of the interventions that have not been assessed yet, therefore addressing the research gaps identified.

Strengths and limitations

- We considered wide range of reporting guidelines as well as their extensions.
- Merging the evidence found in the published and grey literature allowed us to provide a broad picture of how the problem of enhancing adherence to reporting guidelines has been tackled so far and could be faced in the future.
- The screening and data extraction were performed in duplicate.

For peer review only

- We could have missed evidence of possible interventions that may not be present in the published or grey literature but are instead used in practice and continue to be used.

Background

Approximately 85% of all biomedical research today is estimated to be wasted, due, in part, to incomplete or inaccurate reporting (1). The past two decades have given rise to a number of changes in an effort to help authors and the broader scientific community properly report research methods and findings, which would allow them to contribute to the broader goal of combating waste in biomedical research. The most prominent of these changes has been the inception of reporting guidelines for different study types, data, and clinical areas (2).

The vast majority of reporting guidelines have not yet been assessed as to whether they help improve the reporting of research (3), but some, such as the Consolidated Standards of Reporting Trials (CONSORT) for the reporting of randomised controlled trials (RCTs) (4), have been shown to enhance the completeness of reporting (5,6).

Dozens of systematic reviews have explored the extent of adherence to some reporting guidelines in certain areas of health research (7–10). Saaman et al. (11) went one step further and performed a systematic review of systematic reviews assessing adherence to reporting guidelines. As they considered a broad range of clinical areas and study designs, their results provided a global picture of adherence to reporting guidelines in health research. Although some studies reported acceptable overall levels of completeness of reporting and found that it had improved since the introduction of certain reporting guidelines such as CONSORT, the authors of most of the reviews (43 of 50, 86%) concluded that more improvement is needed or that adherence to reporting guidelines was inadequate, poor, medium or suboptimal. Therefore, it is warranted to explore and develop strategies to improve the current levels of adherence to reporting guidelines.

In recent years, several initiatives aiming to improve adherence to reporting guidelines have been proposed, some of which have already been evaluated. For example, the effect of journal endorsement of reporting guidelines (3,5,6) and the implementation of writing aid tools for authors such as the CONSORT-based web tool (COBWEB) (12) have been assessed. While some of these strategies have not been shown to have a benefit (3), others report better but still suboptimal levels of reporting (5,6) or even clear benefits (12,13).

As mentioned, several reviews have analysed the quality of reporting in different clinical areas and for different study types (7–10). However, no scoping review has been performed that provides a global picture of different strategies aiming to improve adherence to reporting guidelines. Given the low levels of completeness of reporting in health research that have been observed (11), along with the imperative need to take further actions for mitigating this problem, we considered that performing such a scoping review was warranted.

In addition to analysing the implementation and effect of interventions that have already been evaluated, we aimed to gather other possible strategies that could be implemented and evaluated in the future.

For clarification, some relevant terms used throughout the scoping are defined in Box 1, which is based on Stevens et al. (3).

Box 1: relevant definitions in the context of this scoping review

For peer review only

Adherence: Action(s) taken by authors to ensure that a research report is compliant with the items recommended by the appropriate/relevant reporting guideline. These can take place before or after the first version of the manuscript is published.

Endorsement: Action(s) taken by journals to indicate their support for the use of one or more reporting guideline(s) by authors submitting research reports for consideration.

Implementation: Action(s) taken by journals to ensure that authors adhere to an endorsed reporting guideline and that therefore published papers are completely reported.

Complete reporting: Pertains to the state of reporting of a study report and whether it is compliant with all the items recommended by the appropriate/relevant reporting guideline.

Methods

As presented in the published protocol (14), this scoping review follows the methodology manual published by the Joanna Briggs Institute for scoping reviews (15).

Objectives

The scoping review questions are:

1. What interventions to improve adherence to reporting guidelines in health research have been evaluated?
2. What further interventions to improve adherence to reporting guidelines have been performed or suggested but never evaluated?

We aimed to analyse and classify the interventions found for both questions in order to obtain a wide picture of how the problem of adhering better to reporting guidelines has been tackled so far and can be tackled in the future.

Eligibility criteria

We included:

1. Studies evaluating interventions aiming to improve adherence to reporting guidelines in health research, irrespective of study design.

peer review only

2. Commentaries, editorials, letters, studies, and online sources describing possible interventions to improve adherence to reporting guidelines that have been performed or suggested but never evaluated.

The reporting guidelines considered were those shown on 8 May 2017 on the EQUATOR (Enhancing the QUAlity and Transparency Of Health Research) Network website (16) as “Reporting Guidelines for main study types”. In addition, we included QUOROM (Quality of Reporting of Meta-analyses), since it was the precursor of PRISMA. Supplementary file 1 shows all reporting guidelines considered.

We considered the following languages: English, Spanish, French, German and Catalan.

Exclusion criteria

We have excluded references that include interventions that do not specifically aim to improve the completeness of reporting, even though these interventions may actually influence completeness. For example, we have excluded clinical trial registration even though it may enhance completeness of reporting, because its main goals are to improve clinical trial transparency while also reducing publication and selective reporting biases.

Search strategy and study selection

On 8 May 2017, we searched PubMed, EMBASE, and Cochrane Library databases for articles published between 1 January 1996 and 31 March 2017, in accordance with our scheduled search (14). The detailed search terms for PubMed can be found in the protocol.

The retrieved studies were exported into Mendeley and duplicates were automatically removed using it. One reviewer (DB) first screened the titles and abstracts for eligibility. Each of the other two reviewers (JJK and EC) was randomly assigned 50% of the references and screened the titles and abstracts independently of the first reviewer. The reviewers classified the references into one of the following groups:

A) Evaluated: Includes references describing interventions to improve adherence to reporting guidelines that have been empirically assessed.

For peer review only

B) Non-evaluated: Includes references describing interventions to improve adherence to reporting guidelines that have been performed or suggested but never evaluated.

C) Unclear: Includes references (i) containing vague statements such as "Authors, editors, and journals have to adhere better to reporting guidelines to improve the quality of reporting" or "greater efforts have to be made by authors to check that their research is compliant with [the relevant reporting guideline]", or (ii) not having the abstract available.

D) Excluded: Includes references (i) not describing interventions to improve adherence to any of the reporting guidelines considered and (ii) describing but not evaluating certain interventions that have already been classified as evaluated.

Disagreements were solved by discussion among the reviewers.

Second, one reviewer (DB) examined the full-text of all group A and B references to confirm the previous classification, then all group C references to reclassify them either as group A, B, or D. Re-classification was verified by the initial reviewer (JJK or EC). Finally, one reviewer (DB) ensured literature saturation by searching the reference lists of included studies, the lists of articles citing them according to PubMed, and the individual studies included in two relevant systematic reviews (3,6).

In addition, we performed a grey literature search, which included: the websites of networks and organizations promoting the use of reporting guidelines (i.e., EQUATOR Network and National Library of Medicine Research Reporting Guidelines and Initiatives); work groups of medical journal editors (i.e., International Committee of Medical Journal Editors (ICMJE) and World Association of Medical Editors (WAME)); biomedical journal publishers (i.e., BMJ Publishing Group and BioMed Central); funding agencies (i.e., National Institute of Health (NIH) and European Research Council); online platforms of post-publication peer review (i.e., PubPeer and ScienceOpen); and the abstract books of the past editions of the International Congress on Peer Review and Biomedical Publication.

For peer review only

Some of the included references were described in studies co-authored by some of the authors this scoping review. These references underwent the same process of screening, data extraction, and data synthesis as the others.

Data extraction

A data extraction form was developed to collect the information necessary for data synthesis. Two reviewers (DB, JJK) independently performed a pilot data extraction on a random sample of 5 articles and subsequently refined the form.

Extracted data included:

1. Publication characteristics: title, year of publication, author, author's affiliation country, and field of study.
2. Characteristics of the intervention:
    a. Classification as evaluated or non-evaluated.
    b. Research stage: education, grant writing, protocol writing, manuscript writing, submission, journal peer review, copy-editing, and post-publication.
    c. Rationale of the intervention, which refers to the deduced reasons why the intervention is evaluated or proposed.
    d. For evaluated interventions: details of the intervention, study design (e.g. RCT, before-after, etc.), reporting guidelines considered and format (checklist, bullet points and/or examples), period of intervention, number of journals and articles involved, effect size of the intervention on adherence to reporting guidelines and measure used to assess this effect.
3. Relevant conclusions.

Two reviewers (DB, JJK) independently performed data extraction for all studies except for the individual studies of the two systematic reviews evaluating journal endorsement of reporting guidelines (3,6), since none of these studies described further interventions and their results had already been reported in these reviews. Discrepancies between reviewers were discussed and solved by consensus.

For peer review only

Data synthesis

Following data extraction, interventions to improve adherence to reporting guidelines were categorised as follows:

1. Training on the practical use of reporting guidelines: mentoring of different stakeholders on the practical use of reporting guidelines.
2. Enhancing accessibility and understanding: dissemination of reporting guidelines and the improvement of authors' understanding of their content.
3. Encouraging adherence: suggestions and tools to facilitate compliance.
4. Checking adherence and providing feedback: checking the level of compliance and indicating incorrect or missing items.
5. Involvement of experts: interaction and cooperation on methodology and reporting.

One reviewer (DB) performed the initial categorization, which was verified and refined by the other two reviewers (JJK and EC).

Furthermore, we determined the existing gaps in research on the evaluation of interventions to improve adherence to reporting guidelines. More specifically, we identified which categories of interventions and which research stages have not been addressed so far in studies evaluating interventions.

Deviations from the protocol

In order to better capture the most relevant aspects of the included studies, the original data extraction form proposed in the protocol was modified. We removed the health care area of the studies included, refined the research stages considered, and included more details on the implementation of the evaluated interventions.

Patients and public involvement

No patients or public were involved in the study.

For peer review only

Results

The database search yielded 1399 citations after deduplication (see Figure 1). Screening of titles and abstracts resulted in a first classification, after which 435 papers were included for full text review. We also reviewed the full text of 24 additional references found through forward citation searching. Furthermore, a grey literature search yielded 7 additional references. Finally, 109 references were included. Some of these interventions appeared in more than one reference and some of the references contained more than one intervention. 90 of these references (86 observational and 4 randomised studies) described 11 evaluated interventions and the other 19 (12 research studies, 2 editorials, 2 blogs, 1 commentary, 1 essay, and 1 perspective) described 20 non-evaluated interventions. Figure 2 displays these 31 interventions according to their categorization and the research stage where they can be performed. Moreover, Table 1 shows all interventions in a tabular format together with their rationale.

For peer review only

Group	Intervention	Rationale
**Training on the practical use of RGs**	Introduction of RGs & journalology into graduate curricula (18-22)	To introduce good research reporting habits early in young researchers' scientific careers.
	Student's development of protocols for coursework and research using RGs (21)	
	Funder's support of author training on RGs (23)	Authors, editors, and peer reviewers have insufficient training in issues related to reporting.
	Training for peer reviewers and editors on RGs by journals (22,23)	
**Enhancing accessibility and understanding**	Dissemination of RGs by scientific associations (24)	A large number of researchers are not aware of the existence of RGs.
	Translation of RGs to further languages (25)	Language barriers may affect the proper use of RGs.
	Development of expanded database of examples for each RG (26)	Authors need more examples of good reporting to properly understand certain items.
**Encouraging adherence**	Author use of RGs as a template for grant application proposals (21)	Using RGs in early stages may facilitate completeness of reporting of published research.
	Required checklist for ethics approval application (11)	
	Funder's requirement of checklists in author's report (21,108)	
	Author use of the writing aid tool COBWEB (12)	A) Authors need help to successfully adhere to RGs at the writing stage and B) Dividing RG items into bullet points and providing examples might help.
	Author use of a structured approach for reporting research (47,112)	A) To help authors avoid omissions, B) to aid reviewers and editors in appraising articles and C) to allow more efficient data extraction during the systematic review process.
	Author markup of the manuscript to indicate where each RG item is addressed (109)	
	Editorial statement endorsing certain RGs (27–46,48–106,113)	Authors read editorial statements and follow "Instructions to authors".
	Recommendation or requirement to follow RGs in the "Instructions to	

	authors" (27–46,48–106,113)	
	Requirement to submit a RG checklist together with the manuscript indicating page numbers corresponding to each item (27–46,48–106,113)	Authors may not consider editorial statements or recommendations in "Instructions to authors" to be important. Compulsory submission of checklists or text mark-up may encourage authors to be more compliant with RGs.
	Requirement to populate and submit a RG checklist with text from the manuscript (114)	
	Journal development of core versions of RGs containing key items (110)	Focusing on the most important items could be more effective than considering the whole checklist.
	Guidance to authors on manuscript preparation by publication officers (111)	Trained journal officers may enhance authors' compliance with RGs during manuscript preparation.
	Suggestion for peer reviewers to use RGs (107)	Peer reviewers often do not detect reporting flaws. Therefore, they may need to follow a more systematic approach and use RGs.
	Editor's questions to peer reviewers about whether the authors have followed RGs (115)	
**Checking adherence and providing feedback**	Completeness of reporting check by editors (117)	Requiring checklists at submission does not guarantee adherence. Editors and peer reviewers have to check whether submitted papers are compliant with RGs.
	Peer review against RGs (118)	
	Internal peer review against RGs by a trained editorial assistant (120)	It is extremely unlikely that the average clinical peer reviewer has the methodological expertise to check a paper against RGs.
	Implementation of the automatic tool Statreviewer (121)	
	Email to authors to revise the manuscript according to RGs (13)	It might be more effective to ask authors for adherence to RGs during the revision process because they will do anything to get their paper published.
	Implementation of the tool WebCONSORT (119)	
	Completeness of reporting check at copy-editing (122)	Copy-editing and post-publication offer alternate time points to improve adherence to RGs.
	Post- publication peer review (123)	
**Involvement of**	Statistician involvement (78,128-130)	Professionals with specific knowledge of RGs

**experts**	Medical writer involvement (108)	might help authors when designing, conducting or reporting their research.

Among the 11 evaluated interventions identified, we found a variety of measures used to assess their effect on adherence to reporting guidelines, including:

- Score for completeness of reporting for each paper, either assigning different or equal weights to RG items, on a 0-10 scale.
- Percentage of items reported for each paper.
- Percentage of compliance per RG item.
- Score for the Manuscript Quality Assessment Instrument (17) for each paper.

Due to the heterogeneity of these measures and for the sake of clarity, we prefer to omit the information on the exact effect sizes in the main body of the manuscript and show it in Supplementary file 2, together with the implementation details of the evaluated interventions. In this way, these effects can be understood in an appropriate context.

Research gaps identified (see Figure 3) included the evaluation of interventions (i) on training on the use of reporting guidelines and improving understanding of these, and (ii) at early stages of research (education, grant writing or protocol writing), and (iii) after the final acceptance of the manuscript (copyediting or post-publication peer review).

Hereafter, we describe the interventions found for each category (Table 1 and Supplementary file 2 summarise these interventions).

Training on the practical use of reporting guidelines

Four non-evaluated interventions related to educating different stakeholders on the practical use of reporting guidelines were found (18-23).

In a first step, health profession schools could incorporate reporting guidelines into curricula that address research methodology and publication standards (18–22). In line with this, students could develop protocols for coursework and research using reporting guidelines such as SPIRIT (randomised trials) and PRISMA-P (systematic reviews), and

For peer review only

educators may encourage adherence to those guidelines and grade the protocols using them (21). For their part, funders may consider supporting author training on reporting guidelines (23). Finally, journals or publishers may consider investing resources in training editors and reviewers on the content and use of reporting guidelines (22,23).

Enhancing accessibility and understanding

We identified three non-evaluated interventions focused on increasing the awareness of the existence of reporting guidelines, as well as the authors' understanding of content of these (24-26).

First, international scientific associations may play an important role in disseminating and popularizing reporting guidelines to large audiences (24). Second, reporting guideline developers might consider translating them to new languages that have not been addressed yet (25). Finally, further databases of examples of good reporting for different reporting guidelines that are accessible to authors can be developed, as has been done for CONSORT (26).

Encouraging adherence

Fourteen interventions found were associated with different strategies to facilitate compliance with reporting guidelines (11,12,21,27–115). Six of these were evaluated (47)(12,27–46,48–107,113).

Funders might require authors to use reporting guidelines as a template for grant application proposals (21). Later on, research ethics boards may require that protocols submitted for ethical approval clearly state which reporting guidelines the study will be using based on the study design, and that reporting guideline checklists are part of the application for ethics approval (11). Funders could also encourage adherence to reporting guidelines by asking for reporting guideline checklists as part of the authors' report (21,108).

One initiative to support authors adhering to reporting guidelines at the writing stage of the manuscript has been COBWEB, a writing aid tool that aims to help authors adequately combine the different extensions of the CONSORT statement (12). This tool divided the CONSORT items into bullet points showing the key elements that need to be

For peer review only

reported together with examples of adequate reporting. The impact of COBWEB was evaluated in a randomised trial that showed a large effect of this intervention (12) (see Supplementary file 2 for more details about this and other evaluated interventions). A second option to support authors at manuscript writing is that they follow a more structured approach. For example, ClinicalTrials.gov requires authors to report key information in a tabular format when registering a study or making available its results (116). This has been shown to be effective: some results posted on this platform, especially harms, are more complete than those in corresponding journal articles reporting the same trials (47). Another possibility to improve the structure of manuscripts is to include new subheadings corresponding to different reporting guideline items within the traditional IMRaD format (Introduction, Methods, Results, and Discussion), as the American Journal of Orthodontics and Dentofacial Orthopedics (AJO-DO) proposed (112). Finally, authors may also avoid omissions when writing the manuscript if mark up the text and highlight where each item of the relevant checklist is addressed (109).

At manuscript submission stage, different editorial actions have been taken to improve adherence to reporting guidelines. The most popular is what has traditionally been defined as journal endorsement of reporting guidelines, which is usually defined as one or more of the three following interventions: (a) journal editorial statement endorsing certain reporting guidelines; (b) requirement or recommendation in journal's 'Instructions to Authors' to follow certain reporting guidelines when preparing their manuscript; or (c) requirement for authors to submit the appropriate reporting guideline checklist together with their manuscript indicating page numbers corresponding to each item (6). Dozens of observational studies have explored the possible effect of journal endorsement of different reporting guidelines in different clinical areas (27–46,48–106,113). A recent systematic review focused on CONSORT evaluations showed relative but suboptimal improvements in the completeness of reporting in journals by following the aforementioned policies (6), while another systematic review considering 9 other guidelines showed no improvements (3).

Journals might also consider other strategies to enhance adherence to reporting guidelines at submission. A first option could be to develop shorter, core versions of

For peer review only

reporting guidelines containing key items, which could be provided to authors as part of the submission process (110). Second, they might introduce publication officers in order to provide guidance to authors on preparing manuscripts for submission (111). Third, editors may ask authors to populate the relevant checklist with text from their manuscript and not accept a submission unless this is provided (114).

Finally, editors may suggest that peer reviewers use reporting guidelines (107). In addition, by asking peer reviewers questions about whether the author has followed reporting guidelines, this might be an indirect way to encourage them (115).

Checking adherence and providing feedback

Eight interventions were related to monitoring level of compliance with reporting guidelines of the manuscripts and providing instructions to authors on how to improve the reporting of missing or incorrect items (13,117–123). Four of them were evaluated (13,117–119).

Some journals have opted for implementing reporting guidelines at peer review. First, an associate editor may assess manuscripts for adherence to the relevant reporting guideline and ask authors to make changes accordingly (117). This process may be repeated until the associate editor thinks that the manuscript can move to the next step of the review process, leading to an editorial decision. This intervention was evaluated at the AJO-DO and showed satisfactory results: 33 of 37 items reached perfect compliance (117). Second, peer reviewers could also assess the manuscripts against the appropriate checklist (118). While the observed effect of this intervention was slightly positive, it was smaller than hypothesized. In fact, investigators pointed out that authors tended to comply better with suggestions coming from standard reviews rather than from reviews against reporting guidelines, implying that it might be difficult to adhere to high methodological standards at late stages of research if these standards are not considered earlier in the research process. Third, journals could also ask trained editorial assistants to check manuscripts against reporting guidelines (120) or to implement automatic peer review tools such as Statreviewer (124), software that automatically checks adherence to reporting guidelines and evaluates the appropriate use and reporting of statistical tests (121). Currently, its performance is being assessed through

For peer review only

For peer review only - http://bmjopen.bmj.com/site/about/guidelines.xhtml

a pilot trial in collaboration with four BioMed Central Journals (121). In any of those cases, emails could be sent to authors asking them to revise the manuscript according to guidelines (13). To do this, the EQUATOR Network has provided standard letters that can be used a) after checks by an editor or a single peer reviewer, b) after full peer review, or c) alongside acceptance (125). Furthermore, at the time of author revision of the manuscript, Hopewell et al. found no significant effect when incorporating WebCONSORT, a web-based tool that generates a unique list of items customised to the trial design, to the revision process of journals that endorsed CONSORT but had no active policy for implementing it (119). Finally, in a late stage of the publication process, copyediting of the manuscript could also ensure that all items are covered (122).

Once the paper is published, the scientific community could use online platforms of post-publication peer review such as PubPeer (126) or ScienceOpen (127) to evaluate the adherence to reporting guidelines of published articles and to provide feedback to authors (123).

Involvement of experts

Two interventions identified implied interaction and cooperation between authors and experts on methodology and reporting at different stages of research (78,108,128–130). One of them was evaluated (78,128–130).

On the one hand, statisticians (or epidemiologists or other quantitative methodologists) may get involved in the design, conduct or reporting of the study might contribute to properly reporting key areas such as sample size calculation, randomization, blinding, and appropriate statistical analysis (129). While three studies found a statistically significant positive relationship between CONSORT scores and statistician involvement (78,129,130), another one did not (128). On the other hand, it has been hypothesized that the involvement of medical writers during the manuscript writing stage of research could improve the completeness of reporting (108).

Interventions described in papers co-authored by authors of this scoping review

For peer review only

25 (of 109) included references describing 21 (of 31) included interventions were co-authored by at least one of the authors of this scoping review (12,13,63,67,74,76,80,104,107,111,114,115,20,117–120,123,21–23,26,47,54,55).

Discussion

In this scoping review, we identified 31 interventions to improve adherence to reporting guidelines. We have also determined the gaps in research on the evaluation of this type of interventions. By considering a wide range of reporting guidelines as well as their extensions and merging the evidence found in the published and grey literature, this review provides a broad picture of how the problem of enhancing adherence to reporting guidelines has been tackled so far and could be faced in the future.

This study reveals that it is primarily journals that have made most of the efforts to improve adherence to reporting guidelines in health research – although they can certainly do more. Typically, their strategies range from making available editorial statements that endorse certain reporting guidelines, recommending or requiring authors to follow reporting guidelines in the "Instructions to authors", and requiring authors to submit a reporting guideline checklist together with the manuscript, with page numbers indicated for each item. However, these strategies have been shown not to have the desired effect (3,6,131). Recent research has called for more active and enforced journal policies throughout the editorial process, such as requiring the use of structured approaches with new subheadings adapted to different kinds of study designs (112), which was also found to be beneficial in a new study outside of our search period (132); providing guidance on manuscript preparation (111); making sure the peer review process involves editorial assistants who have specific training on reporting issues (120); and implementing automatic peer review tools (121). Journals will vary in their ability to make some of these strategies effective, depending on factors such as their resources, their guidelines to peer reviewers and the dedication of their editors – many editors and editorial staff work part-time and have limited amount of time.

Moreover, editors' education and performance should be improved. A recent study pointed out that more than a third (39%) of the manuscripts classified as randomised

For peer review only

trials by the editorial staff were not actually randomised trials (119,133). Consequently, it seems difficult to improve author and peer reviewer adherence to reporting guidelines if journal gatekeepers are not properly trained in methodological and reporting issues.

Apart from journals, editors and peer reviewers, other key stakeholders such as medical schools, research funders, universities and other research institutions should also take responsibility regarding this issue. This scoping review provides some strategies to follow. However, as the problem is complex and the possible interventions are varied, enhancing the completeness of reporting most likely depends not so much on any isolated action but on a set of strategies by several different stakeholders. These could be enacted at different stages of research, from education to article post-publication.

For interventions aiming to improve adherence to reporting guidelines, we should require the same level of evidence that we require for interventions to improve health. For this reason, it is striking that we found only 4 published randomised trials that evaluated interventions to improve adherence to reporting guidelines (12,107,118,119). Among these trials, statistically significant effect of the intervention was only observed for the use of the writing aid tool for authors COBWEB (12). While performing an additional review against reporting guidelines showed slightly positive but not significant effect (118), suggesting the use of reporting guidelines to peer reviewers (107) or implementing at the process of author revision of the manuscript the web-based tool WebCONSORT showed no benefit (119). The rest of the evaluations of interventions found (86 of 90) were observational studies, whose results are subject to the influence of confounding factors (6). For example, evaluations of the effect of journal endorsement may be influenced by whether different journals are actively checking that authors adhere to the requirements or recommendations they provide to authors at submission (6). For all these reasons, future randomised trials should be performed to evaluate further interventions to improve adherence to reporting guidelines. Moreover, these trials might consider addressing some of the research gaps identified in this review, such as improving adherence to reporting guidelines at the grant application or protocol writing stages.

A few of the interventions found in this review were shown to enhance adherence to reporting guidelines. However, it is noteworthy there is no evidence that some successful interventions (12,117) have been implemented more widely later. For this reason, more resources and efforts are needed to further implement these interventions in other settings, evaluate the effect, and share the results with the scientific community. In any case, it is important to keep in mind that contemporary publication culture may harm the potential improvements in reporting quality. This could result from the fact that most scientists feel that the primary evaluation tool of their research is the quantity of their scientific output rather than its quality (134); and such attitudes may undermine the potential effect of any intervention to improve adherence to reporting guidelines.

Our scoping review has some limitations. First, we did not formally assess the methodological quality of the studies that evaluated interventions. Second, restricting to certain databases or not having standard search terms for the databases searched may have excluded relevant publications. Third, it is possible that we could have missed evidence of possible interventions that may have never been reflected in the published or grey literature but are instead used in practice and continue to be used. For example, journals might be applying specific editorial strategies that are not publicly available on their websites or in the published literature.

This review is part of a larger project whose next goals are (i) to capture editors' perceptions on the barriers and facilitators of some promising interventions identified in this review, (ii) to explore new possible interventions, and (iii) to evaluate one of these interventions in collaboration with BMJ Open.

Conclusion

Improving adherence to reporting guidelines is one of the key issues in order to enhance complete and accurate reporting and therefore reduce waste in research.

Different stakeholders – such as research funders, ethics boards, and journals – should consider implementing and evaluating some of the interventions identified in this study.

For peer review only

List of abbreviations

AJO-DO: American Journal of Orthodontics and Dentofacial Orthopedics; CONSORT: CONsolidated Standards Of Reporting Trials; COBWEB: CONSORT-based web tool; EQUATOR: Enhancing the QUAlity and Transparency Of Health Research; RCT: Randomised Controlled Trial; RG: Reporting Guideline; SPIRIT: Standard Protocol Items: Recommendations for Interventional Trials; PRISMA: Preferred Reporting Items for Systematic Reviews and Meta-Analyses

Declarations

Funding: This scoping review belongs to the ESR 14 research project from the Methods in Research on Research (MiRoR) project (http://miror-ejd.eu/), which has received funding from the European Union's Horizon 2020 research and innovation programme under the Marie Sklodowska-Curie grant agreement No 676207. DM is supported through a University Research Chair (University of Ottawa).

Competing interests: DA and DM are Directors of the UK and Canadian EQUATOR Centres, respectively. IB is deputy director of French EQUATOR Centre.

Author contributions: All authors contributed to conceptualizing and designing the study. DB, EC, and JJK independently performed screening. DB and JJK independently performed data extraction. DB performed initial data synthesis and EC, IB, DM, DGA, and JJK refined it. DB drafted the manuscript. EC, IB, DM, DGA, and JJK made major revisions. Due to the strong involvement of JJK and EC at several different stages of the study, all authors agreed to consider them joint senior authors of the scoping review, although EC was the only senior author of the protocol. All authors read and approved the final manuscript, which was completed in April 2018. DGA passed away in June 2018 and therefore could not approve the revised manuscript (November 2018).

Availability of data and materials: The datasets used and/or analysed during the current study are available from the corresponding author on reasonable request.

Acknowledgements: The authors thank the MiRoR Project (http://miror-ejd.eu/) and Marie Sklodowska-Curie Actions for their support. The authors also thank Matt Elmore for editorial help.

References

1. Chalmers I, Glasziou P. Avoidable waste in the production and reporting of research evidence. Lancet [Internet]. 2009 Jul [cited 2018 Nov 12];374(9683):86–9. Available from: http://linkinghub.elsevier.com/retrieve/pii/S0140673609603299

2. EQUATOR Network. Library for health research reporting. Available from: http://www.equator-network.org/resource-centre/library-of-health-research-reporting

3. Stevens A, Shamseer L, Weinstein E, Yazdi F, Turner L, Thielman J, et al. Relation of completeness of reporting of health research to journals' endorsement of reporting guidelines: systematic review. BMJ [Internet]. 2014 Jun 25 [cited 2017 Jun 7];348(jun25 2):g3804–g3804. Available from: http://www.bmj.com/cgi/doi/10.1136/bmj.g3804

4. Schulz KF, Altman DG, Moher D, CONSORT Group. CONSORT 2010 statement: updated guidelines for reporting parallel group randomised trials. BMJ [Internet]. 2010 Mar 23 [cited 2018 Mar 3];340:c332. Available from: http://www.ncbi.nlm.nih.gov/pubmed/20332509

5. Plint AC, Moher D, Morrison A, Schulz K, Altman DG, Hill C, et al. Does the CONSORT checklist improve the quality of reports of randomised controlled trials? A systematic review. Med J Aust [Internet]. 2006 Sep 4 [cited 2017 Feb 24];185(5):263–7. Available from: http://www.ncbi.nlm.nih.gov/pubmed/16948622

6. Turner L, Shamseer L, Altman DG, Schulz KF, Moher D, L. T, et al. Does use of the CONSORT Statement impact the completeness of reporting of randomised controlled trials published in medical journals? A Cochrane review. Syst Rev [Internet]. 2012 Nov 29 [cited 2017 Feb 24];1(1):60. Available from: http://systematicreviewsjournal.biomedcentral.com/articles/10.1186/2046-4053-1-60

For peer review only

7. Bereza BG, Machado M, Einarson TR, B.G. B, M. M, T.R. E, et al. Assessing the reporting and scientific quality of meta-analyses of randomized controlled trials of treatments for anxiety disorders. Ann Pharmacother [Internet]. 2008 Oct 26 [cited 2017 Feb 23];42(10):1402–9. Available from: http://www.embase.com/search/results?subaction=viewrecord&from=export&id=L352440213

8. Fung AE, Palanki R, Bakri SJ, Depperschmidt E, Gibson A. Applying the CONSORT and STROBE statements to evaluate the reporting quality of neovascular age-related macular degeneration studies. Ophthalmology [Internet]. 2009 Feb [cited 2017 Jun 26];116(2):286–96. Available from: http://linkinghub.elsevier.com/retrieve/pii/S0161642008009020

9. Rios LP, Odueyungbo A, Moitri MO, Rahman MO, Thabane L, L.P. R, et al. Quality of reporting of randomized controlled trials in general endocrinology literature. J Clin Endocrinol Metab [Internet]. 2008 Oct [cited 2017 Feb 24];93(10):3810–6. Available from: http://press.endocrine.org/doi/10.1210/jc.2008-0817

10. Shea B, Bouter LM, Grimshaw JM, Francis D, Ortiz Z, Wells GA, et al. Scope for improvement in the quality of reporting of systematic reviews. From the Cochrane Musculoskeletal Group. J Rheumatol [Internet]. 2006 Jan [cited 2017 Feb 24];33(1):9–15. Available from: http://www.ncbi.nlm.nih.gov/pubmed/16267878

11. Samaan Z, Mbuagbaw L, Kosa D, Borg Debono V, Dillenburg R, Zhang S, et al. A systematic scoping review of adherence to reporting guidelines in health care literature. J Multidiscip Healthc [Internet]. 2013 May [cited 2017 Jun 26];6:169–88. Available from: http://www.dovepress.com/a-systematic-scoping-review-of-adherence-to-reporting-guidelines-in-he-peer-reviewed-article-JMDH

12. Barnes C, Boutron I, Giraudeau B, Porcher R, Altman DG, Ravaud P. Impact of an online writing aid tool for writing a randomized trial report: the COBWEB (Consort-based WEB tool) randomized controlled trial. BMC Med [Internet]. 2015 Sep 15 [cited 2017 Feb 24];13(1):221. Available from:

For peer review only

http://bmcmedicine.biomedcentral.com/articles/10.1186/s12916-015-0460-y

13. Hopewell S, Ravaud P, Baron G, Boutron I. Effect of editors' implementation of CONSORT guidelines on the reporting of abstracts in high impact medical journals: interrupted time series analysis. 2012 Jun 22 [cited 2017 Jan 16];344(jun22 1). Available from: http://www.ncbi.nlm.nih.gov/pubmed/22730543

14. Blanco D, Kirkham JJ, Altman DG, Moher D, Boutron I, Cobo E. Interventions to improve adherence to reporting guidelines in health research: a scoping review protocol. BMJ Open [Internet]. 2017;7(11):e017551. Available from: http://bmjopen.bmj.com/lookup/doi/10.1136/bmjopen-2017-017551

15. Reviewers' Manual. 2015; Available from: www.joannabriggs.org

16. EQUATOR Network. Available from: http://www.equator-network.org/

17. Goodman SN, Berlin J, Fletcher SW, Fletcher RH. Manuscript quality before and after peer review and editing at Annals of Internal Medicine. Ann Intern Med [Internet]. 1994 Jul 1 [cited 2018 Nov 16];121(1):11–21. Available from: http://www.ncbi.nlm.nih.gov/pubmed/8198342

18. Ma B, Qi G, Lin X, Wang T, Chen Z, Yang K. Epidemiology, quality, and reporting characteristics of systematic reviews of acupuncture interventions published in Chinese journals. J Altern Complement Med [Internet]. 2012 Sep [cited 2017 Jul 17];18(9):813–7. Available from: http://online.liebertpub.com/doi/abs/10.1089/acm.2011.0274

19. Larson EL, Cortazal M. Publication guidelines need widespread adoption. J Clin Epidemiol [Internet]. 2012 Mar [cited 2017 Jul 21];65(3):239–46. Available from: http://linkinghub.elsevier.com/retrieve/pii/S0895435611002290

20. Sarkis-Onofre R, Cenci MS, Moher D, Pereira-Cenci T. Research Reporting Guidelines in Dentistry: A Survey of Editors. Braz Dent J [Internet]. 2017 [cited 2017 Nov 9];28(1):3–8. Available from: http://dx.doi.org/10.1590/0103-

For peer review only

6440201601426

21. Shamseer L, Moher D, Clarke M, Ghersi D, Liberati A, Petticrew M, et al. Preferred reporting items for systematic review and meta-analysis protocols (PRISMA-P) 2015: elaboration and explanation. BMJ [Internet]. 2015 Jan 2 [cited 2017 Feb 24];349(jan02 1):g7647–g7647. Available from: http://www.bmj.com/cgi/doi/10.1136/bmj.g7647

22. Simera I, Moher D, Hirst A, Hoey J, Schulz KF, Altman DG. Transparent and accurate reporting increases reliability, utility, and impact of your research: reporting guidelines and the EQUATOR Network. BMC Med [Internet]. 2010;8(1):24. Available from: http://bmcmedicine.biomedcentral.com/articles/10.1186/1741-7015-8-24

23. Glasziou P, Altman DG, Bossuyt P, Boutron I, Clarke M, Julious S, et al. Reducing waste from incomplete or unusable reports of biomedical research. Lancet [Internet]. 2014 Jan 18 [cited 2017 Sep 29];383(9913):267–76. Available from: http://www.ncbi.nlm.nih.gov/pubmed/24411647

24. Verbeek J. Moose Consort Strobe and Miame Stard Remark or how can we improve the quality of reporting studies. Scand J Work Environ Health [Internet]. 2008 Jun [cited 2017 Feb 24];34(3):165–7. Available from: http://www.ncbi.nlm.nih.gov/pubmed/18728905

25. Kim KH, Kang JW, Lee MS, Lee J-D. Assessment of the quality of reporting in randomised controlled trials of acupuncture in the Korean literature using the CONSORT statement and STRICTA guidelines. BMJ Open [Internet]. 2014 Jul 29 [cited 2017 Jul 17];4(7):e005068. Available from: http://bmjopen.bmj.com/cgi/doi/10.1136/bmjopen-2014-005068

26. Moher D, Hopewell S, Schulz KF, Altman DG. Resources for authors of reports of randomized trials: harnessing the wisdom of authors, editors, and readers. Trials [Internet]. 2011;12(1):98. Available from: http://trialsjournal.biomedcentral.com/articles/10.1186/1745-6215-12-98

For peer review only

27. Faunce TA, Buckley NA. Of consents and CONSORTs: reporting ethics, law, and human rights in RCTs involving monitored overdose of healthy volunteers pre and post the "CONSORT" guidelines. J Toxicol Clin Toxicol [Internet]. 2003 [cited 2017 Nov 9];41(2):93–9. Available from: http://www.ncbi.nlm.nih.gov/pubmed/12733843

28. Halpern SH, Darani R, Douglas MJ, Wight W, Yee J, S.H. H, et al. Compliance with the CONSORT checklist in obstetric anaesthesia randomised controlled trials. Int J Obstet Anesth [Internet]. 2004 Oct [cited 2017 Feb 24];13(4):207–14. Available from: http://www.ncbi.nlm.nih.gov/pubmed/15477048

29. Hewitt C, Hahn S, Torgerson DJ, Watson J, Bland JM. Adequacy and reporting of allocation concealment: review of recent trials published in four general medical journals. BMJ [Internet]. 2005 May 7 [cited 2017 Nov 9];330(7499):1057–8. Available from: http://www.bmj.com/cgi/doi/10.1136/bmj.38413.576713.AE

30. Greenfield MLVH, Rosenberg AL, O???Reilly M, Shanks AM, Sliwinski MJ, Nauss MD. The Quality of Randomized Controlled Trials in Major Anesthesiology Journals. Anesth Analg [Internet]. 2005 Jun [cited 2017 Nov 9];100(6):1759–64. Available from: http://www.ncbi.nlm.nih.gov/pubmed/15920210

31. Llorca J, Martínez-Sanz F, Prieto-Salceda D, Fariñas-Alvarez C, Chinchón MV, Quinones D, et al. Quality of controlled clinical trials on glaucoma and intraocular high pressure. J Glaucoma [Internet]. 2005 Jun [cited 2017 Nov 9];14(3):190–5. Available from: http://www.ncbi.nlm.nih.gov/pubmed/15870599

32. Haahr MT, Hróbjartsson A. Who is blinded in randomized clinical trials? A study of 200 trials and a survey of authors. Clin Trials [Internet]. 2006;3(4):360–5. Available from: http://www.ncbi.nlm.nih.gov/pubmed/17060210

33. Kober T, Trelle S, Engert A. Reporting of randomized controlled trials in Hodgkin lymphoma in biomedical journals. J Natl Cancer Inst [Internet]. 2006 May 3 [cited 2017 Feb 24];98(9):620–5. Available from:

For peer review only

http://www.ncbi.nlm.nih.gov/pubmed/16670387

34. Biondi-Zoccai GGL, Lotrionte M, Abbate A, Testa L, Remigi E, Burzotta F, et al. Compliance with QUOROM and quality of reporting of overlapping meta-analyses on the role of acetylcysteine in the prevention of contrast associated nephropathy: case study. BMJ [Internet]. 2006 Jan 28 [cited 2017 Nov 9];332(7535):202–9. Available from: http://www.bmj.com/cgi/doi/10.1136/bmj.38693.516782.7C

35. Balasubramanian SP, Wiener M, Alshameeri Z, Tiruvoipati R, Elbourne D, Reed MW, et al. Standards of reporting of randomized controlled trials in general surgery: Can we do better? Ann Surg [Internet]. 2006 Nov [cited 2017 Feb 24];244(5):663–7. Available from: http://www.ncbi.nlm.nih.gov/pubmed/17060756

36. Dias S, McNamee R, Vail A, S. D, R. M, A. V, et al. Evidence of improving quality of reporting of randomized controlled trials in subfertility. Hum Reprod [Internet]. 2006 Oct [cited 2017 Feb 24];21(10):2617–27. Available from: http://www.ncbi.nlm.nih.gov/pubmed/16793995

37. Smidt N, Rutjes AWS, van der Windt DAWM, Ostelo RWJG, Bossuyt PM, Reitsma JB, et al. The quality of diagnostic accuracy studies since the STARD statement: Has it improved? Neurology [Internet]. 2006 Sep 12 [cited 2018 Mar 12];67(5):792–7. Available from: http://www.ncbi.nlm.nih.gov/pubmed/16966539

38. Coppus SFPJ, van der Veen F, Bossuyt PMM, Mol BWJ, S.F.P.J. C, F. van der V, et al. Quality of reporting of test accuracy studies in reproductive medicine: impact of the Standards for Reporting of Diagnostic Accuracy (STARD) initiative. Fertil Steril [Internet]. 2006 Nov [cited 2017 Jun 26];86(5):1321–9. Available from: http://www.embase.com/search/results?subaction=viewrecord&from=export&id=L44602037

39. Tiruvoipati R, Balasubramanian SP, Atturu G, Peek GJ, Elbourne D. Improving the

For peer review only

quality of reporting randomized controlled trials in cardiothoracic surgery: the way forward. J Thorac Cardiovasc Surg [Internet]. 2006 Aug [cited 2017 Jul 21];132(2):233–40. Available from: http://www.ncbi.nlm.nih.gov/pubmed/16872940

40. Mahoney J, Ellison J. Assessing the quality of glucose monitor studies: a critical evaluation of published reports. Clin Chem [Internet]. 2007 Jun 19 [cited 2017 Nov 9];53(6):1122–8. Available from: http://www.clinchem.org/cgi/doi/10.1373/clinchem.2006.083493

41. Poolman RW, Abouali JAK, Conter HJ, Bhandari M. Overlapping systematic reviews of anterior cruciate ligament reconstruction comparing hamstring autograft with bone-patellar tendon-bone autograft: why are they different? J Bone Joint Surg Am [Internet]. 2007 Jul 1 [cited 2017 Nov 9];89(7):1542–52. Available from: http://jbjs.org/cgi/doi/10.2106/JBJS.F.01292

42. Spring B, Pagoto S, Knatterud G, Kozak A, Hedeker D. Examination of the analytic quality of behavioral health randomized clinical trials. J Clin Psychol [Internet]. 2007 Jan [cited 2017 Nov 9];63(1):53–71. Available from: http://www.ncbi.nlm.nih.gov/pubmed/17115429

43. Agha R, Cooper D, Muir G. The reporting quality of randomised controlled trials in surgery: A systematic review. Int J Surg [Internet]. 2007 Dec [cited 2017 Nov 9];5(6):413–22. Available from: http://www.ncbi.nlm.nih.gov/pubmed/18029237

44. Kane RL, Wang J, Garrard J. Reporting in randomized clinical trials improved after adoption of the CONSORT statement. J Clin Epidemiol [Internet]. 2007 Mar [cited 2017 Nov 9];60(3):241–9. Available from: http://linkinghub.elsevier.com/retrieve/pii/S0895435606002575

45. Paranjothy B, Shunmugam M, Azuara-Blanco A, B. P, M. S, A. A-B, et al. The quality of reporting of diagnostic accuracy studies in glaucoma using scanning laser polarimetry. J Glaucoma [Internet]. 2007 Dec [cited 2017 Feb

For peer review only

24];16(8):670–5. Available from: http://www.ncbi.nlm.nih.gov/pubmed/18091453

46. Johnson ZK, Siddiqui MAR, Azuara-Blanco A, Z.K. J, M.A.R. S, A. A-B, et al. The Quality of Reporting of Diagnostic Accuracy Studies of Optical Coherence Tomography in Glaucoma. Ophthalmology [Internet]. 2007 Sep [cited 2017 Feb 24];114(9):1607–12. Available from: http://www.ncbi.nlm.nih.gov/pubmed/17434589

47. Riveros C, Dechartres A, Perrodeau E, Haneef R, Boutron I, Ravaud P. Timing and Completeness of Trial Results Posted at ClinicalTrials.gov and Published in Journals. Dickersin K, editor. PLoS Med [Internet]. 2013 Dec 3 [cited 2018 Nov 19];10(12):e1001566. Available from: https://dx.plos.org/10.1371/journal.pmed.1001566

48. Hind D, Booth A. Do health technology assessments comply with QUOROM diagram guidance? An empirical study. BMC Med Res Methodol [Internet]. 2007 Nov 20 [cited 2017 Jul 17];7:49. Available from: http://www.ncbi.nlm.nih.gov/pubmed/18021461

49. Lee PE, Fischer HD, Rochon PA, Gill SS, Herrmann N, Bell CM, et al. Published randomized controlled trials of drug therapy for dementia often lack complete data on harm. J Clin Epidemiol [Internet]. 2008 Nov [cited 2017 Nov 22];61(11):1152–60. Available from: http://linkinghub.elsevier.com/retrieve/pii/S0895435607004829

50. Pat K, Dooms C, Vansteenkiste J, K. P, C. D, J. V, et al. Systematic review of symptom control and quality of life in studies on chemotherapy for advanced non-small cell lung cancer: How CONSORTed are the data? Lung Cancer [Internet]. 2008 Oct [cited 2017 Mar 10];62(1):126–38. Available from: http://linkinghub.elsevier.com/retrieve/pii/S0169500208001062

51. Folkes A, Urquhart R, Grunfeld E, A. F, R. U, E. G. Are leading medical journals following their own policies on CONSORT reporting? Contemp Clin Trials

For peer review only

[Internet]. 2008 Nov [cited 2017 Jun 26];29(6):843–6. Available from: http://linkinghub.elsevier.com/retrieve/pii/S1551714408000906

52. Sinha S, Sinha S, Ashby E, Jayaram R, Grocott MPW. Quality of reporting in randomized trials published in high-quality surgical journals. J Am Coll Surg [Internet]. 2009 Nov [cited 2017 Jul 17];209(5):565–571.e1. Available from: http://linkinghub.elsevier.com/retrieve/pii/S107275150901151X

53. Freeman K, Szczepura A, Osipenko L, K. F, A. S, L. O, et al. Non-invasive fetal RHD genotyping tests: a systematic review of the quality of reporting of diagnostic accuracy in published studies. Eur J Obstet Gynecol Reprod Biol [Internet]. 2009 Feb [cited 2017 Jun 26];142(2):91–8. Available from: http://linkinghub.elsevier.com/retrieve/pii/S0301211508003916

54. Uetani K, Nakayama T, Ikai H, Yonemoto N, Moher D, K. U, et al. Quality of reports on randomized controlled trials conducted in Japan: Evaluation of adherence to the CONSORT statement. Intern Med [Internet]. 2009 [cited 2017 Feb 24];48(5):307–13. Available from: http://www.ncbi.nlm.nih.gov/pubmed/19252352

55. Ethgen M, Boutron L, Steg PG, Roy C, Ravaud P, M. E, et al. Quality of reporting internal and external validity data from randomized controlled trials evaluating stents for percutaneous coronary intervention. BMC Med Res Methodol [Internet]. 2009 Apr 9 [cited 2017 Feb 24];9(1):24. Available from: http://www.embase.com/search/results?subaction=viewrecord&from=export&id=L354667428

56. Krzych LJ, Liszka L. No improvement in studies reporting the diagnostic accuracy of B-type natriuretic peptide. Med Sci Monit [Internet]. 2009 May [cited 2017 Nov 9];15(5):SR5-14. Available from: http://www.ncbi.nlm.nih.gov/pubmed/19396053

57. Pagoto SL, Kozak AT, John P, Bodenlos JS, Hedeker D, Spring B, et al. Intention-to-treat analyses in behavioral medicine randomized clinical trials. Int J Behav

For peer review only

Med [Internet]. 2009 Dec 25 [cited 2017 Feb 24];16(4):316–22. Available from: http://link.springer.com/10.1007/s12529-009-9039-3

58. Kidwell CS, Liebeskind DS, Starkman S, Saver JL. Trends in acute ischemic stroke trials through the 20th century. Stroke [Internet]. 2001 Jun [cited 2017 Nov 9];32(6):1349–59. Available from: http://www.ncbi.nlm.nih.gov/pubmed/11387498

59. Han C, Kwak K, Marks DM, Pae C-U, Wu L-T, Bhatia KS, et al. The impact of the CONSORT statement on reporting of randomized clinical trials in psychiatry. Contemp Clin Trials [Internet]. 2009 Mar [cited 2017 Jun 26];30(2):116–22. Available from: http://linkinghub.elsevier.com/retrieve/pii/S1551714408001511

60. Alvarez F, Meyer N, Gourraud PA, Paul C, F. A, N. M, et al. CONSORT adoption and quality of reporting of randomized controlled trials: a systematic analysis in two dermatology journals. Br J Dermatol [Internet]. 2009 Nov [cited 2017 Feb 23];161(5):1159–65. Available from: http://www.embase.com/search/results?subaction=viewrecord&from=export&id=L355782314

61. Wei X, Tiejun L, Cheng W. Current situation on the reporting quality of randomized controlled trials in 5 leading Chinese medical journals. J Med Coll PLA [Internet]. 2009 Apr [cited 2018 Mar 12];24(2):105–11. Available from: http://linkinghub.elsevier.com/retrieve/pii/S1000194809600253

62. Ladd BO, McCrady BS, Manuel JK, Campbell W, B.O. L, B.S. M, et al. Improving the quality of reporting alcohol outcome studies: Effects of the CONSORT statement. Addict Behav [Internet]. 2010 Jul [cited 2017 Feb 24];35(7):660–6. Available from: http://linkinghub.elsevier.com/retrieve/pii/S0306460310000560

63. Yu L-M, Chan A-W, Hopewell S, Deeks JJ, Altman DG. Reporting on covariate adjustment in randomised controlled trials before and after revision of the 2001 CONSORT statement: a literature review. Trials [Internet]. 2010 May 18 [cited 2017 Nov 9];11(1):59. Available from:

For peer review only

http://trialsjournal.biomedcentral.com/articles/10.1186/1745-6215-11-59

64. Areia M, Soares M, Dinis-Ribeiro M. Quality reporting of endoscopic diagnostic studies in gastrointestinal journals: where do we stand on the use of the STARD and CONSORT statements? Endoscopy [Internet]. 2010 Feb 5 [cited 2017 Nov 9];42(2):138–47. Available from: http://www.thieme-connect.de/DOI/DOI?10.1055/s-0029-1243846

65. Delaney M, Meyer E, Cserti-Gazdewich C, Haspel RL, Lin Y, Morris A, et al. A systematic assessment of the quality of reporting for platelet transfusion studies. Transfusion [Internet]. 2010 Oct [cited 2017 Jun 26];50(10):2135–44. Available from: http://doi.wiley.com/10.1111/j.1537-2995.2010.02691.x

66. Flint HE, Harrison JE. How well do reports of clinical trials in the orthodontic literature comply with the CONSORT statement? J Orthod [Internet]. 2010 Dec 16 [cited 2017 Feb 24];37(4):250–61. Available from: http://www.tandfonline.com/doi/full/10.1179/14653121043191

67. Hopewell S, Dutton S, Yu L-M, Chan A-W, Altman DG, S. H, et al. The quality of reports of randomised trials in 2000 and 2006: comparative study of articles indexed in PubMed. BMJ [Internet]. 2010 Mar 23 [cited 2017 Jun 26];340(mar23 1):c723. Available from: http://www.embase.com/search/results?subaction=viewrecord&from=export&id=L358557565

68. Ernst E, Hung SK CY. NCCAM-funded RCTs of herbal medicines: An independent, critical assessment. Perfusion [Internet]. 2011;24:89–102. Available from: https://www.researchgate.net/publication/288438592_NCCAM-funded_RCTs_of_herbal_medicines_An_independent_critical_assessment

69. Sánchez-Thorin JC, Cortés MC, Montenegro M, Villate N, J.C. S-T, M.C. C, et al. The quality of reporting of randomized clinical trials published in Ophthalmology. Ophthalmology [Internet]. 2001 Feb [cited 2017 Feb 24];108(2):410–5. Available from:

For peer review only

http://www.embase.com/search/results?subaction=viewrecord&from=export&id=L32115976

70. Selman TJ, Morris RK, Zamora J, Khan KS. The quality of reporting of primary test accuracy studies in obstetrics and gynaecology: application of the STARD criteria. BMC Womens Health [Internet]. 2011 Mar 23 [cited 2017 Jul 17];11(1):8. Available from: http://bmcwomenshealth.biomedcentral.com/articles/10.1186/1472-6874-11-8

71. Parsons NR, Hiskens R, Price CL, Achten J, Costa ML, N.R. P, et al. A systematic survey of the quality of research reporting in general orthopaedic journals. J Bone Jt Surg - Ser B [Internet]. 2011 Sep 1 [cited 2017 Feb 24];93 B(9):1154–9. Available from: http://www.bjj.boneandjoint.org.uk/cgi/doi/10.1302/0301-620X.93B9.27193

72. Kiehna EN, Starke RM, Pouratian N, Dumont AS. Standards for reporting randomized controlled trials in neurosurgery. J Neurosurg [Internet]. 2011 Feb [cited 2017 Feb 24];114(2):280–5. Available from: http://thejns.org/doi/10.3171/2010.8.JNS091770

73. Strech D, Soltmann B, Weikert B, Bauer M, Pfennig A, D. S, et al. Quality of reporting of randomized controlled trials of pharmacologic treatment of bipolar disorders: A systematic review. J Clin Psychiatry [Internet]. 2011 Sep 15 [cited 2017 Feb 24];72(9):1214–21. Available from: http://article.psychiatrist.com/?ContentType=START&ID=10007267

74. Turner L-A, Singh K, Garritty C, Tsertsvadze A, Manheimer E, Wieland LS, et al. An evaluation of the completeness of safety reporting in reports of complementary and alternative medicine trials. BMC Complement Altern Med [Internet]. 2011 Aug 22 [cited 2017 Jun 26];11(1):67. Available from: http://bmccomplementalternmed.biomedcentral.com/articles/10.1186/1472-6882-11-67

75. Haidich A-B, Birtsou C, Dardavessis T, Tirodimos I, Arvanitidou M, A.-B. H, et al.

For peer review only

The quality of safety reporting in trials is still suboptimal: survey of major general medical journals. J Clin Epidemiol [Internet]. 2011 Feb [cited 2017 Feb 24];64(2):124–35. Available from: http://linkinghub.elsevier.com/retrieve/pii/S0895435610001083

76. Gray R, Sullivan M, Altman DG, Gordon-Weeks AN, R. G, M. S, et al. Adherence of trials of operative intervention to the CONSORT statement extension for nonpharmacological treatments: A comparative before and after study. Ann R Coll Surg Engl [Internet]. 2012 Sep 1 [cited 2017 Feb 24];94(6):388–94. Available from: http://openurl.ingenta.com/content/xref?genre=article&issn=0035-8843&volume=94&issue=6&spage=388

77. Cornelius VR, Sauzet O, Williams JE, Ayis S, Farquhar-Smith P, Ross JR, et al. Adverse event reporting in randomised controlled trials of neuropathic pain: considerations for future practice. Pain [Internet]. 2013 Feb [cited 2017 Nov 22];154(2):213–20. Available from: http://content.wkhealth.com/linkback/openurl?sid=WKPTLP:landingpage&an=00006396-201302000-00009

78. Diaz-Ordaz K, Froud R, Sheehan B, Eldridge S. A systematic review of cluster randomised trials in residential facilities for older people suggests how to improve quality. BMC Med Res Methodol [Internet]. 2013 Oct 22 [cited 2017 Jul 17];13(1):127. Available from: http://bmcmedresmethodol.biomedcentral.com/articles/10.1186/1471-2288-13-127

79. Geha NN, Moseley AM, Elkins MR, Chiavegato LD, Shiwa SR, Costa LOP. The quality and reporting of randomized trials in cardiothoracic physical therapy could be substantially improved. Respir Care [Internet]. 2013 Nov;58(11):1899–906. Available from: http://rc.rcjournal.com/cgi/doi/10.4187/respcare.02379

80. Moher D, Jones A, Lepage L, CONSORT Group (Consolidated Standards for Reporting of Trials). Use of the CONSORT statement and quality of reports of randomized trials: a comparative before-and-after evaluation. JAMA [Internet].

For peer review only

2001 Apr 18 [cited 2017 Jun 7];285(15):1992–5. Available from: http://www.ncbi.nlm.nih.gov/pubmed/11308436

81. Liu LQ, Morris PJ, Pengel LHM, L.Q. L, P.J. M, L.H.M. P, et al. Compliance to the CONSORT statement of randomized controlled trials in solid organ transplantation: A 3-year overview. Transpl Int [Internet]. 2013 Mar [cited 2017 Feb 24];26(3):300–6. Available from: http://www.embase.com/search/results?subaction=viewrecord&from=export&id=L52379666

82. Panic N, Leoncini E, de Belvis G, Ricciardi W, Boccia S, N. P, et al. Evaluation of the endorsement of the preferred reporting items for systematic reviews and meta-analysis (PRISMA) statement on the quality of published systematic review and meta-analyses. Derrick GE, editor. PLoS One [Internet]. 2013 Dec 26 [cited 2017 Jan 15];8(12):e83138. Available from: http://www.embase.com/search/results?subaction=viewrecord&from=export&id=L372005920

83. Fleming PS, Seehra J, Polychronopoulou A, Fedorowicz Z, Pandis N. A PRISMA assessment of the reporting quality of systematic reviews in orthodontics. Angle Orthod [Internet]. 2013 Jan [cited 2017 Nov 9];83(1):158–63. Available from: http://www.ncbi.nlm.nih.gov/pubmed/22720835

84. Péron J, Maillet D, Gan HK, Chen EX, You B, J. P, et al. Adherence to CONSORT adverse event reporting guidelines in randomized clinical trials evaluating systemic cancer therapy: A systematic review. J Clin Oncol [Internet]. 2013 Nov 1 [cited 2017 Feb 24];31(31):3957–63. Available from: http://ascopubs.org/doi/10.1200/JCO.2013.49.3981

85. Tunis AS, McInnes MDF, Hanna R, Esmail K. Association of Study Quality with Completeness of Reporting: Have Completeness of Reporting and Quality of Systematic Reviews and Meta-Analyses in Major Radiology Journals Changed Since Publication of the PRISMA Statement? Radiology [Internet]. 2013 Nov [cited 2018 Mar 12];269(2):413–26. Available from:

For peer review only

http://pubs.rsna.org/doi/10.1148/radiol.13130273

86. Maclean EN, Stone IS, Ceelen F, Garcia-Albeniz X, Sommer WH, Petersen SE. Quality of reporting in cardiac MRI, CT and SPECT diagnostic accuracy studies: Analysis of the impact of STARD criteria. J Cardiovasc Magn Reson [Internet]. 2013 Jun 1 [cited 2017 Feb 24];15(6):61. Available from: http://www.embase.com/search/results?subaction=viewrecord&from=export&id=L70993941

87. Baker D, Lidster K, Sottomayor A, Amor S. Two Years Later: Journals Are Not Yet Enforcing the ARRIVE Guidelines on Reporting Standards for Pre-Clinical Animal Studies. Eisen JA, editor. PLoS Biol [Internet]. 2014 Jan 7 [cited 2017 Sep 29];12(1):e1001756. Available from: http://www.ncbi.nlm.nih.gov/pubmed/24409096

88. Choi J, Jun JH, Kang BK, Kim KH, Lee MS, J. C, et al. Endorsement for improving the quality of reports on randomized controlled trials of traditional medicine journals in Korea: a systematic review. Trials [Internet]. 2014 Nov 5 [cited 2017 Jan 15];15(1):429. Available from: http://trialsjournal.biomedcentral.com/articles/10.1186/1745-6215-15-429

89. Walther S, Schueler S, Tackmann R, Schuetz GM, Schlattmann P, Dewey M, et al. Compliance with STARD checklist among studies of coronary CT angiography: systematic review. Radiology [Internet]. 2014 Apr [cited 2017 Feb 23];271(1):74–86. Available from: http://pubs.rsna.org/doi/10.1148/radiol.13121720

90. Ghimire S, Kyung E, Lee H, Kim E. Oncology trial abstracts showed suboptimal improvement in reporting: a comparative before-and-after evaluation using CONSORT for Abstract guidelines. J Clin Epidemiol [Internet]. 2014 Jun [cited 2017 Feb 24];67(6):658–66. Available from: http://linkinghub.elsevier.com/retrieve/pii/S0895435613004435

91. Devereaux PJ, Manns BJ, Ghali WA, Quan H, Guyatt GH, P.J. D, et al. The

For peer review only

reporting of methodological factors in randomized controlled trials and the association with a journal policy to promote adherence to the Consolidated Standards of Reporting Trials (CONSORT) checklist. Control Clin Trials [Internet]. 2002 Aug [cited 2017 Feb 24];23(4):380–8. Available from: http://www.embase.com/search/results?subaction=viewrecord&from=export&id=L34874890

92. Song T-J, Leng H-F, Zhong LL, Wu T-X, Bian Z-X. CONSORT in China: past development and future direction. Trials [Internet]. 2015 Jun 1 [cited 2017 Jun 26];16(1):243. Available from: http://trialsjournal.biomedcentral.com/articles/10.1186/s13063-015-0769-z

93. Stevely A, Dimairo M, Todd S, Julious SA, Nicholl J, Hind D, et al. An Investigation of the Shortcomings of the CONSORT 2010 Statement for the Reporting of Group Sequential Randomised Controlled Trials: A Methodological Systematic Review. Shamji M, editor. PLoS One [Internet]. 2015 Nov 3 [cited 2017 Feb 24];10(11):e0141104. Available from: http://dx.plos.org/10.1371/journal.pone.0141104

94. Adie S, Ma D, Harris IA, Naylor JM, Craig JC, S. A, et al. Quality of conduct and reporting of meta-analyses of surgical interventions. Ann Surg [Internet]. 2015 Apr [cited 2017 Jun 26];261(4):685–94. Available from: http://content.wkhealth.com/linkback/openurl?sid=WKPTLP:landingpage%7B&%7Dan=00000658-201504000-00012

95. Jull A, Aye PS. Endorsement of the CONSORT guidelines, trial registration, and the quality of reporting randomised controlled trials in leading nursing journals: A cross-sectional analysis. Int J Nurs Stud [Internet]. 2015 Jun [cited 2017 Feb 10];52(6):1071–9. Available from: http://linkinghub.elsevier.com/retrieve/pii/S0020748914003022

96. Bearn DR, Alharbi F. Reporting of clinical trials in the orthodontic literature from 2008 to 2012: observational study of published reports in four major journals. J Orthod [Internet]. 2015 Sep 15 [cited 2017 Feb 24];42(3):186–91. Available

For peer review only

from: http://www.tandfonline.com/doi/full/10.1179/1465313315Y.0000000011

97. Agha RA, Fowler AJ, Limb C, Whitehurst K, Coe R, Sagoo H, et al. Impact of the mandatory implementation of reporting guidelines on reporting quality in a surgical journal: A before and after study. Int J Surg [Internet]. 2016 Jun [cited 2017 Feb 20];30:169–72. Available from: http://linkinghub.elsevier.com/retrieve/pii/S1743919116300620

98. Pouwels KB, Widyakusuma NN, Groenwold RHH, Hak E. Quality of reporting of confounding remained suboptimal after the STROBE guideline. J Clin Epidemiol [Internet]. 2016 Jan [cited 2017 Feb 24];69:217–24. Available from: http://linkinghub.elsevier.com/retrieve/pii/S0895435615003856

99. Rao A, Brück K, Methven S, Evans R, Stel VS, Jager KJ, et al. Quality of reporting and study design of CKD cohort studies assessing mortality in the elderly before and after STROBE: A systematic review. Zhou X, editor. PLoS One [Internet]. 2016 May 11 [cited 2017 Jun 26];11(5):e0155078. Available from: http://dx.plos.org/10.1371/journal.pone.0155078

100. Grob ATM, van der Vaart LR, Withagen MIJ, van der Vaart CH. The quality of reporting of diagnostic accuracy studies in pelvic floor transperineal three-dimensional ultrasound: a systematic review. Ultrasound Obstet Gynecol [Internet]. 2016 Dec 21 [cited 2017 Mar 10]; Available from: http://doi.wiley.com/10.1002/uog.17390

101. Rikos D, Dardiotis E, Tsivgoulis G, Zintzaras E, Hadjigeorgiou GM, D. R, et al. Reporting quality of randomized-controlled trials in multiple sclerosis from 2000 to 2015, based on CONSORT statement. Mult Scler Relat Disord [Internet]. 2016 Sep [cited 2017 Feb 24];9:135–9. Available from: http://linkinghub.elsevier.com/retrieve/pii/S2211034816301225

102. Montori VM, Bhandari M, Devereaux PJ, Manns BJ, Ghali WA, Guyatt GH. In the dark: the reporting of blinding status in randomized controlled trials. J Clin Epidemiol [Internet]. 2002 Aug [cited 2017 Nov 9];55(8):787–90. Available from:

For peer review only - http://bmjopen.bmj.com/site/about/guidelines.xhtml

http://www.ncbi.nlm.nih.gov/pubmed/12384193

103. Bigna JJR, Um LN, Nansseu JRN, J.J.R. B, L.N. U, J.R.N. N. A comparison of quality of abstracts of systematic reviews including meta-analysis of randomized controlled trials in high-impact general medicine journals before and after the publication of PRISMA extension for abstracts: A systematic review and m. Syst Rev [Internet]. 2016 Oct 13 [cited 2017 Jun 26];5(1):174. Available from: http://www.embase.com/search/results?subaction=viewrecord&from=export&id=L612833923

104. Sarkis-Onofre R, Poletto-Neto V, Cenci MS, Pereira-Cenci T, Moher D. Impact of the CONSORT Statement endorsement in the completeness of reporting of randomized clinical trials in restorative dentistry. J Dent [Internet]. 2017 Mar 31 [cited 2017 Feb 24];58:54–9. Available from: http://linkinghub.elsevier.com/retrieve/pii/S0300571217300283

105. Tharyan P, Premkumar TS, Mathew V, Barnabas JP, Manuelraj. Editorial policy and the reporting of randomized controlled trials: a survey of instructions for authors and assessment of trial reports in Indian medical journals (2004-05). Natl Med J India [Internet]. [cited 2017 Nov 9];21(2):62–8. Available from: http://www.ncbi.nlm.nih.gov/pubmed/18807310

106. Lai TYY, Wong VWY, Lam RF, Cheng ACO, Lam DSC, Leung GM. Quality of reporting of key methodological items of randomized controlled trials in clinical ophthalmic journals. Ophthalmic Epidemiol [Internet]. 14(6):390–8. Available from: http://www.ncbi.nlm.nih.gov/pubmed/18161613

107. Cobo E, Selva-O'Callagham A, Ribera JM, Cardellach F, Dominguez R, Vilardell M. Statistical reviewers improve reporting in biomedical articles: A randomized trial. PLoS One. 2007;2(3).

108. Implementing Reporting Guidelines: Why and How, for Journal Editors [Internet]. World Association of Medical Editors. Available from: https://wame.blog/2017/09/17/implementing-reporting-guidelines-why-and-

For peer review only

how-for-journal-editors/

109. Rupinski M, Zagorowicz E, Regula J, Fijuth J, Kraszewska E, Polkowski M, et al. Randomized Comparison of Three Palliative Regimens Including Brachytherapy, Photodynamic Therapy and APC in Patients With Malignant Dysphagia (CONSORT 1a) (Revised II). Am J Gastroenterol [Internet]. 2011 Sep 14 [cited 2018 Mar 4];106(9):1612–20. Available from: http://www.nature.com/articles/ajg2011178

110. Jilka RL. The Road to Reproducibility in Animal Research. J Bone Miner Res [Internet]. 2016 Jul [cited 2017 Jul 21];31(7):1317–9. Available from: http://doi.wiley.com/10.1002/jbmr.2881

111. Moher D, Altman DG, Roberts I, Dirnagl U, Chalmers I, Berlin J. Four Proposals to Help Improve the Medical Research Literature. PLOS Med [Internet]. 2015 Sep 22 [cited 2017 Aug 29];12(9):e1001864. Available from: http://dx.plos.org/10.1371/journal.pmed.1001864

112. Pandis N, Turpin DL, N. P, D.L. T, Pandis N, Turpin DL. Enhancing CONSORT compliance for improved reporting of randomized controlled trials. Am J Orthod Dentofac Orthop [Internet]. 2014 Jan [cited 2017 Jul 21];145(1):1. Available from: http://www.embase.com/search/results?subaction=viewrecord&from=export&id=L372038916

113. Hill CL, LaValley MP, Felson DT. Secular changes in the quality of published randomized clinical trials in rheumatology. Arthritis Rheum [Internet]. 2002 Mar [cited 2017 Nov 9];46(3):779–84. Available from: http://doi.wiley.com/10.1002/art.512

114. Hutton B, Salanti G, Caldwell DM, Chaimani A, Schmid CH, Cameron C, et al. The PRISMA extension statement for reporting of systematic reviews incorporating network meta-analyses of health care interventions: checklist and explanations. Ann Intern Med [Internet]. 2015 Jun 2 [cited 2017 Jun 26];162(11):777–84.

For peer review only

Available from: http://annals.org/article.aspx?doi=10.7326/M14-2385

115. Hirst A, Altman DG. Are peer reviewers encouraged to use reporting guidelines? A survey of 116 health research journals. Cameron DW, editor. PLoS One [Internet]. 2012 Apr 27 [cited 2017 Jun 7];7(4):e35621. Available from: http://dx.plos.org/10.1371/journal.pone.0035621

116. ClinicalTrials.gov. National Library of Medicine (US). Available from: https://clinicaltrials.gov/

117. Pandis N, Shamseer L, Kokich VG, Fleming PS, Moher D, N. P, et al. Active implementation strategy of CONSORT adherence by a dental specialty journal improved randomized clinical trial reporting. J Clin Epidemiol [Internet]. 2014 Sep [cited 2017 Jan 15];67(9):1044–8. Available from: http://linkinghub.elsevier.com/retrieve/pii/S0895435614001140

118. Cobo E, Cortes J, Ribera JM, Cardellach F, Selva-O'Callaghan A, Kostov B, et al. Effect of using reporting guidelines during peer review on quality of final manuscripts submitted to a biomedical journal: masked randomised trial. 2011 Nov;343(nov22 2). Available from: http://www.ncbi.nlm.nih.gov/pubmed/22108262

119. Hopewell S, Boutron I, Altman DG, Barbour G, Moher D, Montori V, et al. Impact of a web-based tool (WebCONSORT) to improve the reporting of randomised trials: results of a randomised controlled trial. BMC Med [Internet]. 2016 Nov 28 [cited 2017 Feb 24];14(1):199. Available from: http://www.ncbi.nlm.nih.gov/pubmed/27894295

120. Hopewell S, Collins GS, Boutron I, Yu L-M, Cook J, Shanyinde M, et al. Impact of peer review on reports of randomised trials published in open peer review journals: retrospective before and after study. BMJ [Internet]. 2014 [cited 2017 Jun 7];349. Available from: http://www.bmj.com/content/349/bmj.g4145

121. A peerless review? Automating methodological and statistical review. Available from: https://blogs.biomedcentral.com/bmcblog/2016/05/23/peerless-review-

For peer review only

automating-methodological-statistical-review/

122. Mbuagbaw L, Thabane M, Vanniyasingam T, Borg Debono V, Kosa S, Zhang S, et al. Improvement in the quality of abstracts in major clinical journals since CONSORT extension for abstracts: a systematic review. Contemp Clin Trials [Internet]. 2014 Jul [cited 2017 Feb 24];38(2):245–50. Available from: http://linkinghub.elsevier.com/retrieve/pii/S1551714414000809

123. Schriger DL, Altman DG. Inadequate post-publication review of medical research. BMJ [Internet]. 2010 Aug 11 [cited 2018 Mar 4];341:c3803. Available from: http://www.ncbi.nlm.nih.gov/pubmed/20702543

124. Statreviewer. Available from: http://www.statreviewer.com/

125. Tools and templates for implementing reporting guidelines. Available from: http://www.equator-network.org/toolkits/using-guidelines-in-journals/tools-and-templates-for-implementing-reporting-guidelines/

126. PubPeer. Available from: https://pubpeer.com/

127. ScienceOpen. Available from: https://www.scienceopen.com/

128. Péron J, You B, Gan HK, Maillet D, Chen EX, Pond GR, et al. Influence of statistician involvement on reporting of randomized clinical trials in medical oncology. Anticancer Drugs [Internet]. 2013 Mar [cited 2017 Feb 24];24(3):306–9. Available from: http://content.wkhealth.com/linkback/openurl?sid=WKPTLP:landingpage&an=00001813-201303000-00010

129. Pandis N, Polychronopoulou A, Eliades T. An assessment of quality characteristics of randomised control trials published in dental journals. J Dent [Internet]. 2010 Sep [cited 2017 Feb 24];38(9):713–21. Available from: http://linkinghub.elsevier.com/retrieve/pii/S0300571210001235

130. Kloukos D, Papageorgiou SN, Doulis I, Petridis H, Pandis N. Reporting quality of randomised controlled trials published in prosthodontic and implantology

For peer review only

1086 journals. J Oral Rehabil [Internet]. 2015 Dec [cited 2017 Feb 24];42(12):914–25. Available from: http://doi.wiley.com/10.1111/joor.12325

1088 131. Blanco D, Biggane AM, Cobo E. Are CONSORT checklists submitted by authors adequately reflecting what information is actually reported in published papers? Trials [Internet]. 2018 Dec 29 [cited 2018 Jan 31];19(1):80. Available from: https://trialsjournal.biomedcentral.com/articles/10.1186/s13063-018-2475-0

1092 132. Koletsi D, Fleming PS, Behrents RG, Lynch CD, Pandis N. The use of tailored subheadings was successful in enhancing compliance with CONSORT in a dental journal. J Dent [Internet]. 2017 Dec [cited 2018 Mar 23];67:66–71. Available from: http://www.ncbi.nlm.nih.gov/pubmed/28941813

1096 133. Cobo E, González JA. Taking advantage of unexpected WebCONSORT results. BMC Med [Internet]. 2016 Dec 5 [cited 2018 Feb 16];14(1):204. Available from: http://bmcmedicine.biomedcentral.com/articles/10.1186/s12916-016-0758-4

1099 134. Tijdink JK, Schipper K, Bouter LM, Maclaine Pont P, de Jonge J, Smulders YM. How do scientists perceive the current publication culture? A qualitative focus group interview study among Dutch biomedical researchers. BMJ Open [Internet]. 2016 Feb 17 [cited 2017 Sep 28];6(2):e008681. Available from: http://bmjopen.bmj.com/lookup/doi/10.1136/bmjopen-2015-008681

1104

1105 Figures, tables and supplementary files

1106 **Figure 1:** PRISMA flow diagram.

1107 **Figure 2:** Typology of interventions to improve adherence to RGs according to type of intervention and research stage. **Legend:** Evaluated interventions are shown in bold.

1109 **Figure 3**: Gaps in research on the evaluation of interventions to improve adherence to reporting guidelines. **Legend:** Each circle represents one intervention. Variables displayed: 1) Circle size: Number of studies evaluating each intervention (bigger = more studies); 2) Circle colour: Study design of those studies (blue for RCTs and green for observational studies) and 3) Circle fill: Kind

For peer review only

of RG implementation (plain for checklist and stripes for bullet points and examples). Research gaps are highlighted in red.

Table 1: Rationale of the interventions identified.

Supplementary file 1: Description of the acronyms and full names of all reporting guidelines considered.

Supplementary file 2: implementation details of the evaluated interventions.

For peer review only

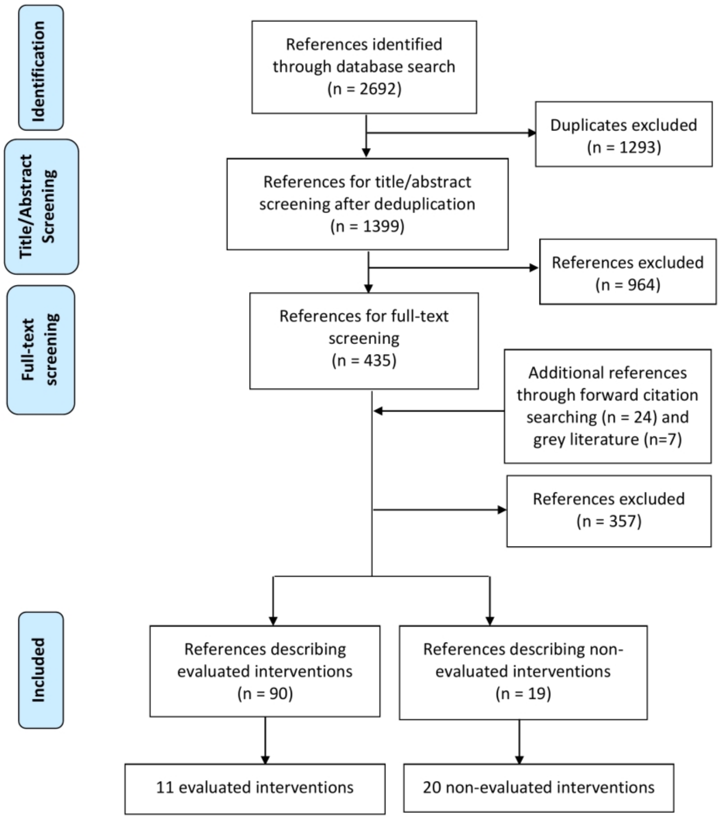

215x279mm (300 x 300 DPI)

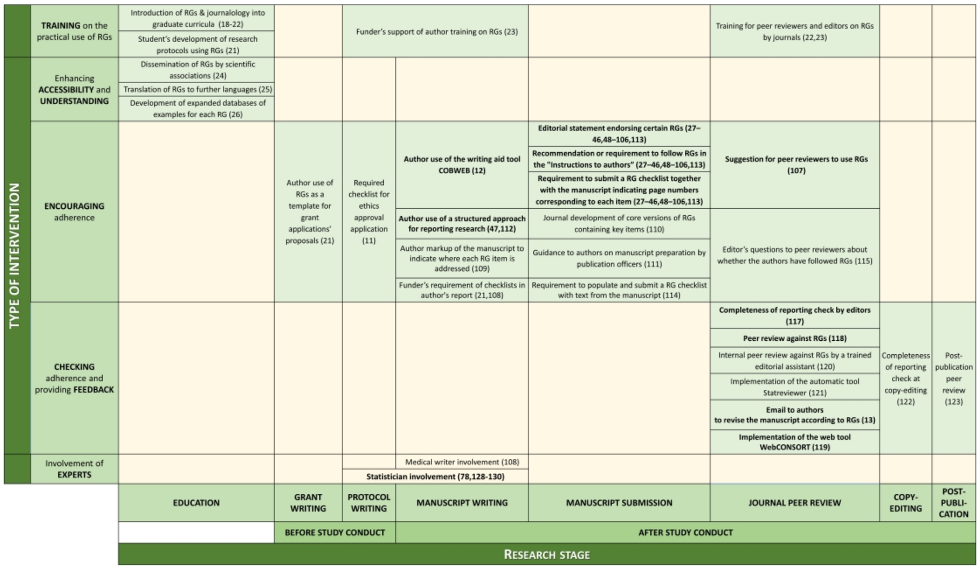

Typology of interventions to improve adherence to RGs according to type of intervention and research stage.
Legend: Evaluated interventions are shown in bold.

670x379mm (300 x 300 DPI)

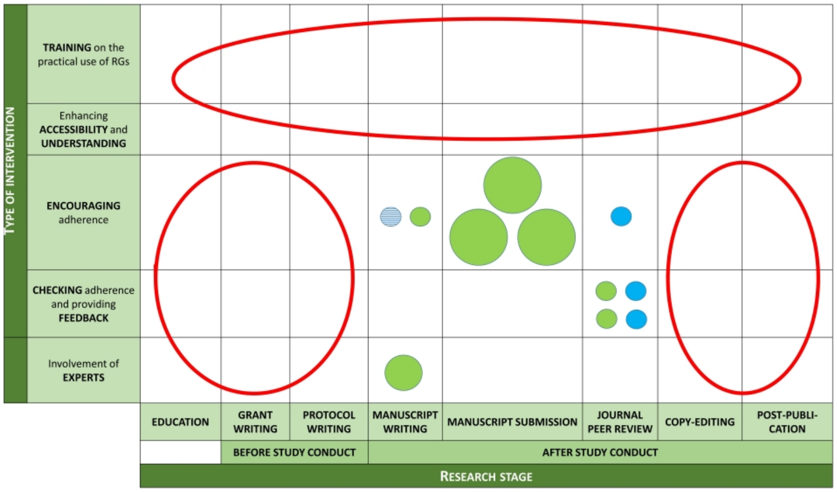

Gaps in research on the evaluation of interventions to improve adherence to reporting guidelines. Legend: Each circle represents one intervention. Variables displayed: 1) Circle size: Number of studies evaluating each intervention (bigger = more studies); 2) Circle colour: Study design of those studies (blue for RCTs and green for observational studies) and 3) Circle fill: Kind of RG implementation (plain for checklist and stripes for bullet points and examples). Research gaps are highlighted in red.

670x379mm (300 x 300 DPI)

Acronym	Full name
CONSORT	Consolidated Standards of Reporting Trials
STROBE	Strengthening the Reporting of Observational Studies in Epidemiology
PRISMA	Preferred Reporting Items for Systematic Reviews and Meta-Analyses
SRQR	Standards for Reporting Qualitative Research
COREQ	Consolidated criteria for Reporting Qualitative research
STARD	Standard Protocol Items: Recommendations for Interventional Trials
TRIPOD	Transparent Reporting of a multivariable prediction model for Individual Prognosis or Diagnosis
SQUIRE	Standards for Quality Improvement Reporting Excellence
CHEERS	Consolidated Health Economic Evaluation Reporting Standards
SPIRIT	Standard Protocol Items: Recommendations for Interventional Trials
PRISMA-P	Preferred Reporting Items for Systematic Review and Meta-Analysis Protocols
CARE	Case Report
AGREE	Appraisal of Guidelines, Research and Evaluation
ARRIVE	Animal Research: Reporting In Vivo Experiments
RIGHT	Reporting Tool for Practice Guidelines in Health Care
QUOROM	Quality of Reporting of Meta-analyses

For peer review only

Type of intervention	Intervention	Number of studies and study design	Details of the intervention	RGs implemented	Format of RG implementation	Measure of adherence to RGs	Effect on adherence to RGs*
Encouraging adherence	Implementation of the writing aid tool COBWEB (12)	1 RCT	Participants have to write the six domains of the methods section of the manuscript for the protocol they receive. They have access to COBWEB tool for a random three of the six domains.	CONSORT & CONSORT extension for non-pharmacological interventions	Bullet points and examples (6 items)	Mean score for completeness of reporting (scale 0–10, items weighted)	Difference of 2.1 (95% CI 1.5-2.7)
	Author use of a structured approach for reporting research (47)	1 Observational study (cross-sectional evaluation)	Results are posted in a standard tabular format without discussions or conclusions.	CONSORT	Checklist (4 items)	Percentage compliance of each RG item	Difference of 0.16, 0.10, 0.18 and 0.36 for each of the 4 items considered
	Journal endorsement (3 interventions, see “Details of the intervention”) (27–46,48–106,113)	80 observational studies (57 cross sectional evaluations of endorsing vs non-endorsing journals, 9 before and after evaluations of endorsing journals before and after endorsement, 14 both kind of evaluations)	A) Editorial statement endorsing certain RGs,<br>B) Recommendation or requirement to follow RGs in the "Instructions to authors", and<br>C) Requirement to submit a RG checklist together with the manuscript indicating page numbers corresponding to each item.	CONSORT (46 of 80)<br>CONSORT extensions (9 of 80)<br>QUOROM (3 of 80)<br>PRISMA (4 of 80)<br>PRISMA extensions (1 of 80)<br>STARD (11 of 80)<br>STROBE (4 of 80)<br>ARRIVE (1 of 80)<br>CONSORT, STROBE and PRISMA (11 of 80)	Checklist (all items)	For CONSORT: percentage of compliance for each item**<br><br>For other RGs: Mean summed score for completeness of reporting	For CONSORT: 25 items improved (see details for each item on figure 2 on Turner et al. (6))<br><br>For CONSORT extension for harms: Difference of 0.04 (99% CI –1.50 to 1.58)

							For PRISMA: Difference of 0.53 (99% CI 0.02 to 1.03)<br><br>For STARD: Difference of 0.52 (99% CI –0.11 to 1.16)<br><br>For STRICTA: Difference of 1.42 (99% CI –0.04 to 2.88)<br><br>For STROBE: Difference of 1.55 (99% CI –3.19 to 6.29)
	Suggestion for peer reviewers to use RGs (107)	1 RCT	Peer reviewers are sent a standard letter encouraging them to use different reporting guidelines. Reviewers are not asked to report whether they used the reporting guideline in reviewing the manuscript.	CONSORT, QUOROM, STARD	Checklist (all items)	Modified version of Manuscript Quality Assessment Instrument (scale 36-180)	Difference of 0.9 (95% CI -0.3 to +2.1)
Checking adherence and providing feedback	Completeness of reporting check by the editors (117)	1 Observational Study (Before and after evaluation)	Initial submissions are vetted by the editor-in-chief. If the submission is considered appropriate, manuscripts are assessed by the associate editor for CONSORT	CONSORT	Checklist (all items)	Percentage of compliance of each RG item	Before – compliance ranges from 0% to 100% (Median 40%)

			adherence. Authors are asked to make changes accordingly until associate editor deems appropriate that they move to the next step of the review process leading to an editorial decision.				After – perfect compliance in 33 out of 37 items
	Additional review against RGs (118)	1 RCT	A senior statistician does an additional review of all papers and provides authors suggestions on how to follow reporting guideline checklists.	STROBE, CONSORT, STARD	Checklist (all items)	Modified version of Manuscript Quality Assessment Instrument (scale 1 to 9)	Difference of 0.25 (95% CI -0.05 to +0.54)
	Active implementation of RG by editors (2 interventions, see "Details of the intervention") (13)	1 Observational study (Interrupted time series evaluation)	A) Email is sent to authors to revise the abstract according to the guidelines at the revision stage and B) Changes are made by the assistant editors of these journals towards the end of the editorial process.	CONSORT extension for abstracts	Checklist (9 of 17 items)	Monthly mean number of items reported (scale 0 to 9)	Difference of 1.5 items
	Implementation of the web-based tool WebCONSORT (119)	1 RCT	Journal editor includes a link to WebCONSORT in the revision letter to authors. Authors are directed to an automatically generated list of items and a flow diagram customised to their specific trial design.	CONSORT & some CONSORT extensions	Checklist (10 of 25 items)	Percentage of items reported for each article	Difference of 0.04 (95% CI −0.02 to +0.10)

Involvement of experts	Statistician involvement (78,128-130)	4 Observational studies (cross sectional evaluations)	Statisticians (or epidemiologists or other quantitative methodologists) are involved in the design, conduct or reporting of the study	CONSORT	Checklist (all items)	Mean score for completeness of reporting (scale 0-10, items not weighted)	In Diaz-Ordaz (78): No global effect provided (see effects for individual items in Table 2 of the paper)<br><br>In Pandis et al. (128): Difference of 0.93<br><br>In Péron et al. (129): No difference in medians<br><br>In Kloukos et al. (130): 0.27

*Difference between adherence to RGs in intervention and non-intervention group. We did not report the CI of the effect size when authors did not report it in the original papers.

**As the 80 individual studies that belong to this category used different measures of adherence to reporting guidelines, we report here the measures used in the two systematic reviews that summarized the pooled results of most of these studies (3,6).

review only

PRISMA-ScR Checklist*

Section/topic	#	Checklist item	Reported on page #
**TITLE**			
Title	1	Identify the report as a scoping review.	1
**ABSTRACT**			
Structured summary	2	Provide a structured summary that includes (as applicable) background, objectives, eligibility criteria, sources of evidence, charting methods, results, and conclusions that relate to the review questions and objectives.	1,2
**INTRODUCTION**			
Rationale	3	Describe the rationale for the review in the context of what is already known. Explain why the review questions/objectives lend themselves to a scoping review approach.	3,4 ("Background")
Objectives	4	Provide an explicit statement of the questions and objectives being addressed with reference to their key elements (e.g., population or participants, concepts, and context) or other relevant key elements used to conceptualize the review questions and/or objectives.	5 ("Objectives")
**METHODS**			
Protocol and registration	5	Indicate whether a review protocol exists; state if and where it can be accessed (e.g., a Web address); and if available, provide registration information, including the registration number.	5 ("Methods")
Eligibility criteria	6	Specify characteristics of the sources of evidence used as eligibility criteria (e.g., years considered, language, and publication status), and provide a rationale.	5,6 ("Eligibility Criteria", "Exclusion Criteria")
Information sources	7	Describe all information sources in the search (e.g., databases with dates of coverage and contact with authors to identify additional sources), as well as the date the most recent search was executed.	6 ("Search Strategy and Study Selection")
Search	8	Present the full electronic search strategy for at least 1 database, including any limits used, such that it could be repeated.	6 ("Search Strategy and Study Selection")
Study selection	9	State the process for selecting sources of evidence (i.e., screening and eligibility) included in the scoping review.	6,7 ("Search Strategy and Study

PRISMA-ScR Checklist*

			Selection")
Data collection process	10	Describe the methods of charting data from the included sources of evidence (e.g., calibrated forms or forms that have been tested by the team before their use, and whether data charting was done independently or in duplicate) and any processes for obtaining and confirming data from investigators.	8 ("Data Extraction")
Data items	11	List and define all variables for which data were sought and any assumptions and simplifications made.	8 ("Data Extraction")
Risk of bias in individual studies	12	If done, provide a rationale for conducting a critical appraisal of included sources of evidence; describe the methods used and how this information was used in any data synthesis (if appropriate).	N/A
Summary measures	13	Not applicable for scoping reviews.	N/A
Synthesis of results	14	Describe the methods of handling and summarizing the data that were charted.	9 ("Data Synthesis)

Section/topic	#	Checklist item 10	Reported on page #
Risk of bias across studies	15	Not applicable for scoping reviews.	N/A
Additional analyses	16	Not applicable for scoping reviews.	N/A
**RESULTS**			
Study selection	17	Give numbers of sources of evidence screened, assessed for eligibility, and included in the review, with reasons for exclusions at each stage, ideally using a flow diagram.	Figure 1
Study characteristics	18	For each source of evidence, present characteristics for which data were charted and provide the citations.	10 ("Results)
Risk of bias within studies	19	If done, present data on critical appraisal of included sources of evidence (see item 12).	N/A
Results of individual studies	20	For each included source of evidence, present the relevant data that were charted that relate to the review questions and objectives.	10 ("Results")
Synthesis of results	21	Summarize and/or present the charting results as they relate to the review questions and objectives.	10-15 ("Results")

For peer review only - http://bmjopen.bmj.com/site/about/guidelines.xhtml

PRISMA-ScR Checklist*

Risk of bias across studies	22	Not applicable for scoping reviews.	
Additional analysis	23	Not applicable for scoping reviews.	
**DISCUSSION**			
Summary of evidence	24	Summarize the main results (including an overview of concepts, themes, and types of evidence available), link to the review questions and objectives, and consider the relevance to key groups.	15 (“Discussion)
Limitations	25	Discuss the limitations of the scoping review process.	17 (“Discussion”)
Conclusions	26	Provide a general interpretation of the results with respect to the review questions and objectives, as well as potential implications and/or next steps.	15-17 (“Discussion)
**FUNDING**			
Funding	27	Describe sources of funding for the included sources of evidence, as well as sources of funding for the scoping review. Describe the role of the funders of the scoping review.	18 (“Declarations – Funding”)

Original source: http://annals.org/aim/fullarticle/2700389/prisma-extension-scoping-reviews-prisma-scr-checklist-explanation

For peer review only

For peer review only - http://bmjopen.bmj.com/site/about/guidelines.xhtml

BMJ Open

A scoping review on interventions to improve adherence to reporting guidelines in health research

Journal:	*BMJ Open*
Manuscript ID	bmjopen-2018-026589.R2
Article Type:	Research
Date Submitted by the Author:	17-Jan-2019
Complete List of Authors:	Blanco, David; Universitat Politècnica de Catalunya, Statistics and Operations Research<br>Altman, Doug; Centre for Statistics in Medicine<br>Moher, David; Ottawa Hospital Research Institute, Centre for Journalology<br>Boutron, Isabelle; Université Paris Descartes, Centre d\'épidémiologie clinique<br>Kirkham, Jamie; University of Liverpool, Biostatistics<br>Cobo, Erik; Universitat Politècnica de Catalunya, Statistics and Operations Research
**Primary Subject Heading**:	Medical publishing and peer review
Secondary Subject Heading:	Medical education and training, Research methods
Keywords:	Scoping review, Quality of reporting, Completeness of reporting, Reporting guidelines, Knowledge synthesis, Adherence

SCHOLARONE™
Manuscripts

A scoping review on interventions to improve adherence to reporting guidelines in health research

Authors

David Blanco[1,2], Doug Altman[3], David Moher[4], Isabelle Boutron[5], Jamie J Kirkham[6*] and Erik Cobo[1*].

*JJK and EC are joint senior authors.

[1]Statistics and Operations Research Department, Universitat Politècnica de Catalunya - Barcelona Tech, Barcelona, Spain.

[2]INSERM, U1153 Epidemiology and Biostatistics Sorbonne Paris Cité Research Center (CRESS), Methods of Therapeutic Evaluation of Chronic Diseases (METHODS) Team, Paris Descartes University, Sorbonne Paris Cité, Paris F-75014, France.

[3]Centre for Statistics in Medicine, University of Oxford, Oxford, UK.

[4]Centre for Journalology, Ottawa Hospital Research Institute, Ottawa, Canada.

[5]Centre d'Epidémiologie Clinique, Université Paris Descartes, Paris, France.

[6]Department of Biostatistics, University of Liverpool, Liverpool, UK.

Correspondence to David Blanco; david.blanco.tena@upc.edu

For peer review only

Keywords

Scoping review, knowledge synthesis, reporting guidelines, completeness of reporting, quality of reporting, adherence

Abstract

Objectives: The goal of this study is to identify, analyse and classify interventions to improve adherence to reporting guidelines in order to obtain a wide picture of how the

problem of enhancing the completeness of reporting of biomedical literature has been tackled so far.

Design: Scoping review.

Search strategy: We searched the MEDLINE, EMBASE, and Cochrane Library databases and conducted a grey literature search for (i) studies evaluating interventions to improve adherence to reporting guidelines in health research and (ii) other types of references describing interventions that have been performed or suggested but never evaluated. The characteristics and effect of the evaluated interventions were analysed. Moreover, we explored the rationale of the interventions identified and determined the existing gaps in research on the evaluation of interventions to improve adherence to reporting guidelines.

Results: 109 references containing 31 interventions (11 evaluated) were included. These were grouped into five categories: (1) training on the use of reporting guidelines, (2) improving understanding, (3) encouraging adherence, (4) checking adherence and providing feedback, and (5) involvement of experts. Additionally, we identified lack of evaluated interventions (i) on training on the use of reporting guidelines and improving their understanding, (ii) at early stages of research, and (iii) after the final acceptance of the manuscript.

Conclusions: This scoping review identified a wide range of strategies to improve adherence to reporting guidelines that can be taken by different stakeholders. Additional research is needed to assess the effectiveness of many of these interventions.

Strengths and limitations

- We considered wide range of reporting guidelines as well as their extensions.
- Merging the evidence found in the published and grey literature allowed us to provide a broad picture of how the problem of enhancing adherence to reporting guidelines has been tackled so far and could be faced in the future.
- The screening and data extraction were performed in duplicate.

For peer review only

- We could have missed evidence of possible interventions that may not be present in the published or grey literature but are instead used in practice and continue to be used.

Background

Approximately 85% of all biomedical research today is estimated to be wasted, due, in part, to incomplete or inaccurate reporting (1). The past two decades have given rise to a number of changes in an effort to help authors and the broader scientific community properly report research methods and findings, which would allow them to contribute to the broader goal of combating waste in biomedical research. The most prominent of these changes has been the inception of reporting guidelines for different study types, data, and clinical areas (2).

The vast majority of reporting guidelines have not yet been assessed as to whether they help improve the reporting of research (3), but some, such as the Consolidated Standards of Reporting Trials (CONSORT) for the reporting of randomised controlled trials (RCTs) (4), have been shown to enhance the completeness of reporting (5,6).

Dozens of systematic reviews have explored the extent of adherence to some reporting guidelines in certain areas of health research (7–10). Saaman et al. (11) went one step further and performed a systematic review of systematic reviews assessing adherence to reporting guidelines. As they considered a broad range of clinical areas and study designs, their results provided a global picture of adherence to reporting guidelines in health research. Although some studies reported acceptable overall levels of completeness of reporting and found that it had improved since the introduction of certain reporting guidelines such as CONSORT, the authors of most of the reviews (43 of 50, 86%) concluded that more improvement is needed or that adherence to reporting guidelines was inadequate, poor, medium or suboptimal. Therefore, it is warranted to explore and develop strategies to improve the current levels of adherence to reporting guidelines.

For peer review only - http://bmjopen.bmj.com/site/about/guidelines.xhtml

In recent years, several initiatives aiming to improve adherence to reporting guidelines have been proposed, some of which have already been evaluated. For example, the effect of journal endorsement of reporting guidelines (3,5,6) and the implementation of writing aid tools for authors such as the CONSORT-based web tool (COBWEB) (12) have been assessed. While some of these strategies have not been shown to have a benefit (3), others report better but still suboptimal levels of reporting (5,6) or even clear benefits (12,13).

As mentioned, several reviews have analysed the quality of reporting in different clinical areas and for different study types (7–10). However, no scoping review has been performed that provides a global picture of different strategies aiming to improve adherence to reporting guidelines. Given the low levels of completeness of reporting in health research that have been observed (11), along with the imperative need to take further actions for mitigating this problem, we considered that performing such a scoping review was warranted.

In addition to analysing the implementation and effect of interventions that have already been evaluated, we aimed to gather other possible strategies that could be implemented and evaluated in the future.

For clarification, some relevant terms used throughout the scoping are defined in Box 1, which is based on Stevens et al. (3).

Box 1: relevant definitions in the context of this scoping review

For peer review only

Adherence: Action(s) taken by authors to ensure that a research report is compliant with the items recommended by the appropriate/relevant reporting guideline. These can take place before or after the first version of the manuscript is published.

Endorsement: Action(s) taken by journals to indicate their support for the use of one or more reporting guideline(s) by authors submitting research reports for consideration.

Implementation: Action(s) taken by journals to ensure that authors adhere to an endorsed reporting guideline and that therefore published papers are completely reported.

Complete reporting: Pertains to the state of reporting of a study report and whether it is compliant with all the items recommended by the appropriate/relevant reporting guideline.

Methods

As presented in the published protocol (14), this scoping review follows the methodology manual published by the Joanna Briggs Institute for scoping reviews (15).

Objectives

The scoping review questions are:

1. What interventions to improve adherence to reporting guidelines in health research have been evaluated?
2. What further interventions to improve adherence to reporting guidelines have been performed or suggested but never evaluated?

We aimed to analyse and classify the interventions found for both questions in order to obtain a wide picture of how the problem of adhering better to reporting guidelines has been tackled so far and can be tackled in the future.

Eligibility criteria

We included:

1. Studies evaluating interventions aiming to improve adherence to reporting guidelines in health research, irrespective of study design.

peer review only

2. Commentaries, editorials, letters, studies, and online sources describing possible interventions to improve adherence to reporting guidelines that have been performed or suggested but never evaluated.

The reporting guidelines considered were those shown on 8 May 2017 on the EQUATOR (Enhancing the QUAlity and Transparency Of Health Research) Network website (16) as "Reporting Guidelines for main study types". In addition, we included QUOROM (Quality of Reporting of Meta-analyses), since it was the precursor of PRISMA. Supplementary file 1 shows all reporting guidelines considered.

We considered the following languages: English, Spanish, French, German and Catalan.

Exclusion criteria

We have excluded references that include interventions that do not specifically aim to improve the completeness of reporting, even though these interventions may actually influence completeness. For example, we have excluded clinical trial registration even though it may enhance completeness of reporting, because its main goals are to improve clinical trial transparency while also reducing publication and selective reporting biases.

Search strategy and study selection

On 8 May 2017, we searched PubMed, EMBASE, and Cochrane Library databases for articles published between 1 January 1996 and 31 March 2017, in accordance with our scheduled search (14). The detailed search terms for PubMed can be found in the protocol.

The retrieved studies were exported into Mendeley and duplicates were automatically removed using it. One reviewer (DB) first screened the titles and abstracts for eligibility. Each of the other two reviewers (JJK and EC) was randomly assigned 50% of the references and screened the titles and abstracts independently of the first reviewer. The reviewers classified the references into one of the following groups:

A) Evaluated: Includes references describing interventions to improve adherence to reporting guidelines that have been empirically assessed.

For peer review only

B) Non-evaluated: Includes references describing interventions to improve adherence to reporting guidelines that have been performed or suggested but never evaluated.

C) Unclear: Includes references (i) containing vague statements such as “Authors, editors, and journals have to adhere better to reporting guidelines to improve the quality of reporting” or “greater efforts have to be made by authors to check that their research is compliant with [the relevant reporting guideline]”, or (ii) not having the abstract available.

D) Excluded: Includes references (i) not describing interventions to improve adherence to any of the reporting guidelines considered and (ii) describing but not evaluating certain interventions that have already been classified as evaluated.

Disagreements were solved by discussion among the reviewers.

Second, one reviewer (DB) examined the full-text of all group A and B references to confirm the previous classification, then all group C references to reclassify them either as group A, B, or D. Re-classification was verified by the initial reviewer (JJK or EC). Finally, one reviewer (DB) ensured literature saturation by searching the reference lists of included studies, the lists of articles citing them according to PubMed, and the individual studies included in two relevant systematic reviews (3,6).

In addition, we performed a grey literature search, which included: the websites of networks and organizations promoting the use of reporting guidelines (i.e., EQUATOR Network and National Library of Medicine Research Reporting Guidelines and Initiatives); work groups of medical journal editors (i.e., International Committee of Medical Journal Editors (ICMJE) and World Association of Medical Editors (WAME)); biomedical journal publishers (i.e., BMJ Publishing Group and BioMed Central); funding agencies (i.e., National Institute of Health (NIH) and European Research Council); online platforms of post-publication peer review (i.e., PubPeer and ScienceOpen); and the abstract books of the past editions of the International Congress on Peer Review and Biomedical Publication.

For peer review only

Some of the included references were described in studies co-authored by some of the authors this scoping review. These references underwent the same process of screening, data extraction, and data synthesis as the others.

Data extraction

A data extraction form was developed to collect the information necessary for data synthesis. Two reviewers (DB, JJK) independently performed a pilot data extraction on a random sample of 5 articles and subsequently refined the form.

Extracted data included:

1. Publication characteristics: title, year of publication, author, author's affiliation country, and field of study.
2. Characteristics of the intervention:
   a. Classification as evaluated or non-evaluated.
   b. Research stage: education, grant writing, protocol writing, manuscript writing, submission, journal peer review, copy-editing, and post-publication.
   c. Rationale of the intervention, which refers to the deduced reasons why the intervention is evaluated or proposed.
   d. For evaluated interventions: details of the intervention, study design (e.g. RCT, before-after, etc.), reporting guidelines considered and format (checklist, bullet points and/or examples), period of intervention, number of journals and articles involved, effect size of the intervention on adherence to reporting guidelines and measure used to assess this effect.
3. Relevant conclusions.

Two reviewers (DB, JJK) independently performed data extraction for all studies except for the individual studies of the two systematic reviews evaluating journal endorsement of reporting guidelines (3,6), since none of these studies described further interventions and their results had already been reported in these reviews. Discrepancies between reviewers were discussed and solved by consensus.

For peer review only - http://bmjopen.bmj.com/site/about/guidelines.xhtml

Data synthesis

Following data extraction, interventions to improve adherence to reporting guidelines were categorised as follows:

1. Training on the practical use of reporting guidelines: mentoring of different stakeholders on the practical use of reporting guidelines.
2. Enhancing accessibility and understanding: dissemination of reporting guidelines and the improvement of authors' understanding of their content.
3. Encouraging adherence: suggestions and tools to facilitate compliance.
4. Checking adherence and providing feedback: checking the level of compliance and indicating incorrect or missing items.
5. Involvement of experts: interaction and cooperation on methodology and reporting.

One reviewer (DB) performed the initial categorization, which was verified and refined by the other two reviewers (JJK and EC).

Furthermore, we determined the existing gaps in research on the evaluation of interventions to improve adherence to reporting guidelines. More specifically, we identified which categories of interventions and which research stages have not been addressed so far in studies evaluating interventions.

We did not perform a meta-analysis of the observational studies assessing journal endorsement of reporting guidelines that were not included in the two systematic reviews previously mentioned (3,6). We considered that, for the purpose of this scoping review, these systematic reviews provided a reliable picture of the impact of this editorial intervention.

Deviations from the protocol

In order to better capture the most relevant aspects of the included studies, the original data extraction form proposed in the protocol was modified. We removed the health care area of the studies included, refined the research stages considered, and included more details on the implementation of the evaluated interventions.

For peer review only

Patients and public involvement

No patients or public were involved in the study.

Results

The database search yielded 1399 citations after deduplication (see Figure 1). Screening of titles and abstracts resulted in a first classification, after which 435 papers were included for full text review. We also reviewed the full text of 24 additional references found through forward citation searching. Furthermore, a grey literature search yielded 7 additional references. Finally, 109 references were included. Some of these interventions appeared in more than one reference and some of the references contained more than one intervention. 90 of these references (86 observational and 4 randomised studies) described 11 evaluated interventions and the other 19 (12 research studies, 2 editorials, 2 blogs, 1 commentary, 1 essay, and 1 perspective) described 20 non-evaluated interventions. Figure 2 displays these 31 interventions according to their categorization and the research stage where they can be performed. Moreover, Table 1 shows all interventions in a tabular format together with their rationale. All interventions reported in this section were found in the literature and do not necessarily correspond to the personal ideas of the scoping review authors.

Among the 11 evaluated interventions identified, we found a variety of measures used to assess their effect on adherence to reporting guidelines, including:

- Score for completeness of reporting for each paper, either assigning different or equal weights to RG items, on a 0-10 scale.
- Percentage of items reported for each paper.
- Percentage of compliance per RG item.
- Score for the Manuscript Quality Assessment Instrument (17) for each paper.

Due to the heterogeneity of these measures and for the sake of clarity, we prefer to omit the information on the exact effect sizes in the main body of the manuscript and show it in Supplementary file 2, together with the implementation details of the

For peer review only

evaluated interventions. In this way, these effects can be understood in an appropriate context.

Research gaps identified (see Figure 3) included the evaluation of interventions (i) on training on the use of reporting guidelines and improving understanding of these, and (ii) at early stages of research (education, grant writing or protocol writing), and (iii) after the final acceptance of the manuscript (copyediting or post-publication peer review).

Hereafter, we describe the interventions found for each category (Table 1 and Supplementary file 2 summarise these interventions).

Training on the practical use of reporting guidelines

Four non-evaluated interventions related to educating different stakeholders on the practical use of reporting guidelines were found (18-23).

In a first step, health profession schools could incorporate reporting guidelines into curricula that address research methodology and publication standards (18–22). In line with this, students could develop protocols for coursework and research using reporting guidelines such as SPIRIT (randomised trials) and PRISMA-P (systematic reviews), and educators may encourage adherence to those guidelines and grade the protocols using them (21). For their part, funders may consider supporting author training on reporting guidelines (23). Finally, journals or publishers may consider investing resources in training editors and reviewers on the content and use of reporting guidelines (22,23).

Enhancing accessibility and understanding

We identified three non-evaluated interventions focused on increasing the awareness of the existence of reporting guidelines, as well as the authors' understanding of content of these (24-26).

First, international scientific associations may play an important role in disseminating and popularizing reporting guidelines to large audiences (24). Second, reporting guideline developers might consider translating them to new languages that have not been addressed yet (25). Finally, further databases of examples of good reporting for

For peer review only

different reporting guidelines that are accessible to authors can be developed, as has been done for CONSORT (26).

Encouraging adherence

Fourteen interventions found were associated with different strategies to facilitate compliance with reporting guidelines (11,12,21,27–115). Six of these were evaluated (47)(12,27–46,48–107,113).

Funders might require authors to use reporting guidelines as a template for grant application proposals (21). Later on, research ethics boards may require that protocols submitted for ethical approval clearly state which reporting guidelines the study will be using based on the study design, and that reporting guideline checklists are part of the application for ethics approval (11). Funders could also encourage adherence to reporting guidelines by asking for reporting guideline checklists as part of the authors' report (21,108).

One initiative to support authors adhering to reporting guidelines at the writing stage of the manuscript has been COBWEB, a writing aid tool that aims to help authors adequately combine the different extensions of the CONSORT statement (12). This tool divided the CONSORT items into bullet points showing the key elements that need to be reported together with examples of adequate reporting. The impact of COBWEB was evaluated in a randomised trial that showed a large effect of this intervention (12) (see Supplementary file 2 for more details about this and other evaluated interventions). A second option to support authors at manuscript writing is that they follow a more structured approach. For example, ClinicalTrials.gov requires authors to report key information in a tabular format when registering a study or making available its results (116). This has been shown to be effective: some results posted on this platform, especially harms, are more complete than those in corresponding journal articles reporting the same trials (47). Another possibility to improve the structure of manuscripts is to include new subheadings corresponding to different reporting guideline items within the traditional IMRaD format (Introduction, Methods, Results, and Discussion), as the American Journal of Orthodontics and Dentofacial Orthopedics (AJO-DO) proposed (112). Finally, authors may also avoid omissions when writing the

For peer review only

manuscript if mark up the text and highlight where each item of the relevant checklist is addressed (109).

At manuscript submission stage, different editorial actions have been taken to improve adherence to reporting guidelines. The most popular is what has traditionally been defined as journal endorsement of reporting guidelines, which is usually defined as one or more of the three following interventions: (a) journal editorial statement endorsing certain reporting guidelines; (b) requirement or recommendation in journal's 'Instructions to Authors' to follow certain reporting guidelines when preparing their manuscript; or (c) requirement for authors to submit the appropriate reporting guideline checklist together with their manuscript indicating page numbers corresponding to each item (6). Dozens of observational studies have explored the possible effect of journal endorsement of different reporting guidelines in different clinical areas (27–46,48–106,113). A recent systematic review focused on CONSORT evaluations showed relative but suboptimal improvements in the completeness of reporting in journals by following the aforementioned policies (6), while another systematic review considering 9 other guidelines showed no improvements (3).

Journals might also consider other strategies to enhance adherence to reporting guidelines at submission. A first option could be to develop shorter, core versions of reporting guidelines containing key items, which could be provided to authors as part of the submission process (110). Second, they might introduce publication officers in order to provide guidance to authors on preparing manuscripts for submission (111). Third, editors may ask authors to populate the relevant checklist with text from their manuscript and not accept a submission unless this is provided (114).

Finally, editors may suggest that peer reviewers use reporting guidelines (107). In addition, by asking peer reviewers questions about whether the author has followed reporting guidelines, this might be an indirect way to encourage them (115).

Checking adherence and providing feedback

Eight interventions were related to monitoring level of compliance with reporting guidelines of the manuscripts and providing instructions to authors on how to improve

For peer review only

the reporting of missing or incorrect items (13,117–123). Four of them were evaluated (13,117–119).

Some journals have opted for implementing reporting guidelines at peer review. First, an associate editor may assess manuscripts for adherence to the relevant reporting guideline and ask authors to make changes accordingly (117). This process may be repeated until the associate editor thinks that the manuscript can move to the next step of the review process, leading to an editorial decision. This intervention was evaluated at the AJO-DO and showed satisfactory results: 33 of 37 items reached perfect compliance (117). Second, peer reviewers could also assess the manuscripts against the appropriate checklist (118). While the observed effect of this intervention was slightly positive, it was smaller than hypothesized. In fact, investigators pointed out that authors tended to comply better with suggestions coming from standard reviews rather than from reviews against reporting guidelines, implying that it might be difficult to adhere to high methodological standards at late stages of research if these standards are not considered earlier in the research process. Third, journals could also ask trained editorial assistants to check manuscripts against reporting guidelines (120) or to implement automatic peer review tools such as Statreviewer (124), software that automatically checks adherence to reporting guidelines and evaluates the appropriate use and reporting of statistical tests (121). Currently, its performance is being assessed through a pilot trial in collaboration with four BioMed Central Journals (121). In any of those cases, emails could be sent to authors asking them to revise the manuscript according to guidelines (13). To do this, the EQUATOR Network has provided standard letters that can be used a) after checks by an editor or a single peer reviewer, b) after full peer review, or c) alongside acceptance (125). Furthermore, at the time of author revision of the manuscript, Hopewell et al. found no significant effect when incorporating WebCONSORT, a web-based tool that generates a unique list of items customised to the trial design, to the revision process of journals that endorsed CONSORT but had no active policy for implementing it (119). Finally, in a late stage of the publication process, copyediting of the manuscript could also ensure that all items are covered (122).

Once the paper is published, the scientific community could use online platforms of post-publication peer review such as PubPeer (126) or ScienceOpen (127) to evaluate

For peer review only

For peer review only - http://bmjopen.bmj.com/site/about/guidelines.xhtml

the adherence to reporting guidelines of published articles and to provide feedback to authors (123).

Involvement of experts

Two interventions identified implied interaction and cooperation between authors and experts on methodology and reporting at different stages of research (78,108,128–130). One of them was evaluated (78,128–130).

On the one hand, statisticians (or epidemiologists or other quantitative methodologists) may get involved in the design, conduct or reporting of the study might contribute to properly reporting key areas such as sample size calculation, randomization, blinding, and appropriate statistical analysis (129). While three studies found a statistically significant positive relationship between CONSORT scores and statistician involvement (78,129,130), another one did not (128). On the other hand, it has been hypothesized that the involvement of medical writers during the manuscript writing stage of research could improve the completeness of reporting (108).

Interventions described in papers co-authored by authors of this scoping review

25 (of 109) included references describing 21 (of 31) included interventions were co-authored by at least one of the authors of this scoping review (12,13,63,67,74,76,80,104,107,111,114,115,20,117–120,123,21–23,26,47,54,55).

Discussion

In this scoping review, we identified 31 interventions to improve adherence to reporting guidelines. We have also determined the gaps in research on the evaluation of this type of interventions. By considering a wide range of reporting guidelines as well as their extensions and merging the evidence found in the published and grey literature, this review provides a broad picture of how the problem of enhancing adherence to reporting guidelines has been tackled so far and could be faced in the future.

This study reveals that most published research aimed at improving adherence to reporting guidelines has been conducted in journals. Typically, journal strategies range

For peer review only

from making available editorial statements that endorse certain reporting guidelines, recommending or requiring authors to follow reporting guidelines in the "Instructions to authors", and requiring authors to submit a reporting guideline checklist together with the manuscript, with page numbers indicated for each item. However, these strategies have been shown not to have the desired effect (3,6,131). Recent research has called for more active and enforced journal policies throughout the editorial process, such as requiring the use of structured approaches with new subheadings adapted to different kinds of study designs (112), which was also found to be beneficial in a new study outside of our search period (132); providing guidance on manuscript preparation (111); making sure the peer review process involves editorial assistants who have specific training on reporting issues (120); and implementing automatic peer review tools (121). Journals will vary in their ability to make some of these strategies effective, depending on factors such as their resources, their guidelines to peer reviewers and the dedication of their editors – many editors and editorial staff work part-time and have limited amount of time.

Moreover, editors' education and performance should be improved. A recent study pointed out that more than a third (39%) of the manuscripts classified as randomised trials by the editorial staff were not actually randomised trials (119,133). Consequently, it seems difficult to improve author and peer reviewer adherence to reporting guidelines if journal gatekeepers are not properly trained in methodological and reporting issues.

Apart from journals, editors and peer reviewers, other key stakeholders such as medical schools, research funders, universities and other research institutions should also take responsibility regarding this issue. This scoping review provides some strategies to follow. However, as the problem is complex and the possible interventions are varied, enhancing the completeness of reporting most likely depends not so much on any isolated action but on a set of strategies by several different stakeholders. These could be enacted at different stages of research, from education to article post-publication.

For interventions aiming to improve adherence to reporting guidelines, we should require the same level of evidence that we require for interventions to improve health. For this reason, it is striking that we found only 4 published randomised trials that

For peer review only

evaluated interventions to improve adherence to reporting guidelines (12,107,118,119). Among these trials, statistically significant effect of the intervention was only observed for the use of the writing aid tool for authors COBWEB (12). While performing an additional review against reporting guidelines showed slightly positive but not significant effect (118), suggesting the use of reporting guidelines to peer reviewers (107) or implementing at the process of author revision of the manuscript the web-based tool WebCONSORT showed no benefit (119). The rest of the evaluations of interventions found (86 of 90) were observational studies, whose results are subject to the influence of confounding factors. As already mentioned, the impact of journal endorsement on completeness of reporting was suboptimal (3,6). However, completeness of reporting improved remarkably when reporting guidelines were actively implemented by editors (e.g. if editors perform a completeness of reporting check of the manuscript (117)) and when research results were posted in a tabular format without discussion or conclusions (47). Future randomised trials should consider evaluating these interventions or addressing some of the research gaps identified in this review, such as improving adherence to reporting guidelines at the grant application or protocol writing stages.

A few of the interventions found in this review were shown to enhance adherence to reporting guidelines. However, it is noteworthy there is no evidence that some successful interventions (12,117) have been implemented more widely later. For this reason, more resources and efforts are needed to further implement these interventions in other settings, evaluate the effect, and share the results with the scientific community. In any case, it is important to keep in mind that contemporary publication culture may harm the potential improvements in reporting quality. This could result from the fact that most scientists feel that the primary evaluation tool of their research is the quantity of their scientific output rather than its quality (134); and such attitudes may undermine the potential effect of any intervention to improve adherence to reporting guidelines.

For peer review only

Our scoping review has some limitations. First, we did not formally assess the methodological quality of the studies that evaluated interventions. Second, restricting to certain databases or not having standard search terms for the databases searched may have excluded relevant publications. Third, it is possible that we could have missed evidence of possible interventions that may have never been reflected in the published or grey literature but are instead used in practice and continue to be used. For example, journals might be applying specific editorial strategies that are not publicly available on their websites or in the published literature.

Conclusion

Improving adherence to reporting guidelines is one of the key issues in order to enhance complete and accurate reporting and therefore reduce waste in research.

Different stakeholders – such as research funders, ethics boards, and journals – should consider implementing and evaluating some of the interventions identified in this study.

List of abbreviations

AJO-DO: American Journal of Orthodontics and Dentofacial Orthopedics; CONSORT: CONsolidated Standards Of Reporting Trials; COBWEB: CONSORT-based web tool; EQUATOR: Enhancing the QUAlity and Transparency Of Health Research; RCT: Randomised Controlled Trial; RG: Reporting Guideline; SPIRIT: Standard Protocol Items: Recommendations for Interventional Trials; PRISMA: Preferred Reporting Items for Systematic Reviews and Meta-Analyses

Declarations

Funding: This scoping review belongs to the ESR 14 research project from the Methods in Research on Research (MiRoR) project (http://miror-ejd.eu/), which has received funding from the European Union's Horizon 2020 research and innovation programme under the Marie Sklodowska-Curie grant agreement No 676207. DM is supported through a University Research Chair (University of Ottawa).

Competing interests: DA and DM are Directors of the UK and Canadian EQUATOR Centres,

For peer review only

respectively. IB is deputy director of French EQUATOR Centre.

Author contributions: All authors contributed to conceptualizing and designing the study. DB, EC, and JJK independently performed screening. DB and JJK independently performed data extraction. DB performed initial data synthesis and EC, IB, DM, DGA, and JJK refined it. DB drafted the manuscript. EC, IB, DM, DGA, and JJK made major revisions. Due to the strong involvement of JJK and EC at several different stages of the study, all authors agreed to consider them joint senior authors of the scoping review, although EC was the only senior author of the protocol. All authors read and approved the final manuscript, which was completed in April 2018. DGA passed away in June 2018 and therefore could not approve the revised manuscript (November 2018).

Availability of data and materials: The datasets used and/or analysed during the current study are available from the corresponding author on reasonable request.

Acknowledgements: The authors thank the MiRoR Project (http://miror-ejd.eu/) and Marie Sklodowska-Curie Actions for their support. The authors also thank Matt Elmore for editorial help. This review is part of a larger project whose next goals are (i) to capture editors' perceptions on the barriers and facilitators of some promising interventions identified in this review, (ii) to explore new possible interventions, and (iii) to evaluate one of these interventions in collaboration with BMJ Open.

References

1. Chalmers I, Glasziou P. Avoidable waste in the production and reporting of research evidence. Lancet [Internet]. 2009 Jul [cited 2018 Nov 12];374(9683):86–9. Available from: http://linkinghub.elsevier.com/retrieve/pii/S0140673609603299

2. EQUATOR Network. Library for health research reporting. Available from: http://www.equator-network.org/resource-centre/library-of-health-research-reporting

3. Stevens A, Shamseer L, Weinstein E, Yazdi F, Turner L, Thielman J, et al. Relation of completeness of reporting of health research to journals' endorsement of

For peer review only

reporting guidelines: systematic review. BMJ [Internet]. 2014 Jun 25 [cited 2017 Jun 7];348(jun25 2):g3804–g3804. Available from: http://www.bmj.com/cgi/doi/10.1136/bmj.g3804

4. Schulz KF, Altman DG, Moher D, CONSORT Group. CONSORT 2010 statement: updated guidelines for reporting parallel group randomised trials. BMJ [Internet]. 2010 Mar 23 [cited 2018 Mar 3];340:c332. Available from: http://www.ncbi.nlm.nih.gov/pubmed/20332509

5. Plint AC, Moher D, Morrison A, Schulz K, Altman DG, Hill C, et al. Does the CONSORT checklist improve the quality of reports of randomised controlled trials? A systematic review. Med J Aust [Internet]. 2006 Sep 4 [cited 2017 Feb 24];185(5):263–7. Available from: http://www.ncbi.nlm.nih.gov/pubmed/16948622

6. Turner L, Shamseer L, Altman DG, Schulz KF, Moher D, L. T, et al. Does use of the CONSORT Statement impact the completeness of reporting of randomised controlled trials published in medical journals? A Cochrane review. Syst Rev [Internet]. 2012 Nov 29 [cited 2017 Feb 24];1(1):60. Available from: http://systematicreviewsjournal.biomedcentral.com/articles/10.1186/2046-4053-1-60

7. Bereza BG, Machado M, Einarson TR, B.G. B, M. M, T.R. E, et al. Assessing the reporting and scientific quality of meta-analyses of randomized controlled trials of treatments for anxiety disorders. Ann Pharmacother [Internet]. 2008 Oct 26 [cited 2017 Feb 23];42(10):1402–9. Available from: http://www.embase.com/search/results?subaction=viewrecord&from=export&id=L352440213

8. Fung AE, Palanki R, Bakri SJ, Depperschmidt E, Gibson A. Applying the CONSORT and STROBE statements to evaluate the reporting quality of neovascular age-related macular degeneration studies. Ophthalmology [Internet]. 2009 Feb [cited 2017 Jun 26];116(2):286–96. Available from: http://linkinghub.elsevier.com/retrieve/pii/S0161642008009020

For peer review only

9. Rios LP, Odueyungbo A, Moitri MO, Rahman MO, Thabane L, L.P. R, et al. Quality of reporting of randomized controlled trials in general endocrinology literature. J Clin Endocrinol Metab [Internet]. 2008 Oct [cited 2017 Feb 24];93(10):3810–6. Available from: http://press.endocrine.org/doi/10.1210/jc.2008-0817

10. Shea B, Bouter LM, Grimshaw JM, Francis D, Ortiz Z, Wells GA, et al. Scope for improvement in the quality of reporting of systematic reviews. From the Cochrane Musculoskeletal Group. J Rheumatol [Internet]. 2006 Jan [cited 2017 Feb 24];33(1):9–15. Available from: http://www.ncbi.nlm.nih.gov/pubmed/16267878

11. Samaan Z, Mbuagbaw L, Kosa D, Borg Debono V, Dillenburg R, Zhang S, et al. A systematic scoping review of adherence to reporting guidelines in health care literature. J Multidiscip Healthc [Internet]. 2013 May [cited 2017 Jun 26];6:169–88. Available from: http://www.dovepress.com/a-systematic-scoping-review-of-adherence-to-reporting-guidelines-in-he-peer-reviewed-article-JMDH

12. Barnes C, Boutron I, Giraudeau B, Porcher R, Altman DG, Ravaud P. Impact of an online writing aid tool for writing a randomized trial report: the COBWEB (Consort-based WEB tool) randomized controlled trial. BMC Med [Internet]. 2015 Sep 15 [cited 2017 Feb 24];13(1):221. Available from: http://bmcmedicine.biomedcentral.com/articles/10.1186/s12916-015-0460-y

13. Hopewell S, Ravaud P, Baron G, Boutron I. Effect of editors' implementation of CONSORT guidelines on the reporting of abstracts in high impact medical journals: interrupted time series analysis. 2012 Jun 22 [cited 2017 Jan 16];344(jun22 1). Available from: http://www.ncbi.nlm.nih.gov/pubmed/22730543

14. Blanco D, Kirkham JJ, Altman DG, Moher D, Boutron I, Cobo E. Interventions to improve adherence to reporting guidelines in health research: a scoping review protocol. BMJ Open [Internet]. 2017;7(11):e017551. Available from: http://bmjopen.bmj.com/lookup/doi/10.1136/bmjopen-2017-017551

For peer review only

15. Reviewers' Manual. 2015; Available from: www.joannabriggs.org

16. EQUATOR Network. Available from: http://www.equator-network.org/

17. Goodman SN, Berlin J, Fletcher SW, Fletcher RH. Manuscript quality before and after peer review and editing at Annals of Internal Medicine. Ann Intern Med [Internet]. 1994 Jul 1 [cited 2018 Nov 16];121(1):11–21. Available from: http://www.ncbi.nlm.nih.gov/pubmed/8198342

18. Ma B, Qi G, Lin X, Wang T, Chen Z, Yang K. Epidemiology, quality, and reporting characteristics of systematic reviews of acupuncture interventions published in Chinese journals. J Altern Complement Med [Internet]. 2012 Sep [cited 2017 Jul 17];18(9):813–7. Available from: http://online.liebertpub.com/doi/abs/10.1089/acm.2011.0274

19. Larson EL, Cortazal M. Publication guidelines need widespread adoption. J Clin Epidemiol [Internet]. 2012 Mar [cited 2017 Jul 21];65(3):239–46. Available from: http://linkinghub.elsevier.com/retrieve/pii/S0895435611002290

20. Sarkis-Onofre R, Cenci MS, Moher D, Pereira-Cenci T. Research Reporting Guidelines in Dentistry: A Survey of Editors. Braz Dent J [Internet]. 2017 [cited 2017 Nov 9];28(1):3–8. Available from: http://dx.doi.org/10.1590/0103-6440201601426

21. Shamseer L, Moher D, Clarke M, Ghersi D, Liberati A, Petticrew M, et al. Preferred reporting items for systematic review and meta-analysis protocols (PRISMA-P) 2015: elaboration and explanation. BMJ [Internet]. 2015 Jan 2 [cited 2017 Feb 24];349(jan02 1):g7647–g7647. Available from: http://www.bmj.com/cgi/doi/10.1136/bmj.g7647

22. Simera I, Moher D, Hirst A, Hoey J, Schulz KF, Altman DG. Transparent and accurate reporting increases reliability, utility, and impact of your research: reporting guidelines and the EQUATOR Network. BMC Med [Internet]. 2010;8(1):24. Available from: http://bmcmedicine.biomedcentral.com/articles/10.1186/1741-7015-8-24

For peer review only

23. Glasziou P, Altman DG, Bossuyt P, Boutron I, Clarke M, Julious S, et al. Reducing waste from incomplete or unusable reports of biomedical research. Lancet [Internet]. 2014 Jan 18 [cited 2017 Sep 29];383(9913):267–76. Available from: http://www.ncbi.nlm.nih.gov/pubmed/24411647

24. Verbeek J. Moose Consort Strobe and Miame Stard Remark or how can we improve the quality of reporting studies. Scand J Work Environ Health [Internet]. 2008 Jun [cited 2017 Feb 24];34(3):165–7. Available from: http://www.ncbi.nlm.nih.gov/pubmed/18728905

25. Kim KH, Kang JW, Lee MS, Lee J-D. Assessment of the quality of reporting in randomised controlled trials of acupuncture in the Korean literature using the CONSORT statement and STRICTA guidelines. BMJ Open [Internet]. 2014 Jul 29 [cited 2017 Jul 17];4(7):e005068. Available from: http://bmjopen.bmj.com/cgi/doi/10.1136/bmjopen-2014-005068

26. Moher D, Hopewell S, Schulz KF, Altman DG. Resources for authors of reports of randomized trials: harnessing the wisdom of authors, editors, and readers. Trials [Internet]. 2011;12(1):98. Available from: http://trialsjournal.biomedcentral.com/articles/10.1186/1745-6215-12-98

27. Faunce TA, Buckley NA. Of consents and CONSORTs: reporting ethics, law, and human rights in RCTs involving monitored overdose of healthy volunteers pre and post the "CONSORT" guidelines. J Toxicol Clin Toxicol [Internet]. 2003 [cited 2017 Nov 9];41(2):93–9. Available from: http://www.ncbi.nlm.nih.gov/pubmed/12733843

28. Halpern SH, Darani R, Douglas MJ, Wight W, Yee J, S.H. H, et al. Compliance with the CONSORT checklist in obstetric anaesthesia randomised controlled trials. Int J Obstet Anesth [Internet]. 2004 Oct [cited 2017 Feb 24];13(4):207–14. Available from: http://www.ncbi.nlm.nih.gov/pubmed/15477048

29. Hewitt C, Hahn S, Torgerson DJ, Watson J, Bland JM. Adequacy and reporting of allocation concealment: review of recent trials published in four general medical

For peer review only

journals. BMJ [Internet]. 2005 May 7 [cited 2017 Nov 9];330(7499):1057–8. Available from: http://www.bmj.com/cgi/doi/10.1136/bmj.38413.576713.AE

30. Greenfield MLVH, Rosenberg AL, O???Reilly M, Shanks AM, Sliwinski MJ, Nauss MD. The Quality of Randomized Controlled Trials in Major Anesthesiology Journals. Anesth Analg [Internet]. 2005 Jun [cited 2017 Nov 9];100(6):1759–64. Available from: http://www.ncbi.nlm.nih.gov/pubmed/15920210

31. Llorca J, Martínez-Sanz F, Prieto-Salceda D, Fariñas-Alvarez C, Chinchón MV, Quinones D, et al. Quality of controlled clinical trials on glaucoma and intraocular high pressure. J Glaucoma [Internet]. 2005 Jun [cited 2017 Nov 9];14(3):190–5. Available from: http://www.ncbi.nlm.nih.gov/pubmed/15870599

32. Haahr MT, Hróbjartsson A. Who is blinded in randomized clinical trials? A study of 200 trials and a survey of authors. Clin Trials [Internet]. 2006;3(4):360–5. Available from: http://www.ncbi.nlm.nih.gov/pubmed/17060210

33. Kober T, Trelle S, Engert A. Reporting of randomized controlled trials in Hodgkin lymphoma in biomedical journals. J Natl Cancer Inst [Internet]. 2006 May 3 [cited 2017 Feb 24];98(9):620–5. Available from: http://www.ncbi.nlm.nih.gov/pubmed/16670387

34. Biondi-Zoccai GGL, Lotrionte M, Abbate A, Testa L, Remigi E, Burzotta F, et al. Compliance with QUOROM and quality of reporting of overlapping meta-analyses on the role of acetylcysteine in the prevention of contrast associated nephropathy: case study. BMJ [Internet]. 2006 Jan 28 [cited 2017 Nov 9];332(7535):202–9. Available from: http://www.bmj.com/cgi/doi/10.1136/bmj.38693.516782.7C

35. Balasubramanian SP, Wiener M, Alshameeri Z, Tiruvoipati R, Elbourne D, Reed MW, et al. Standards of reporting of randomized controlled trials in general surgery: Can we do better? Ann Surg [Internet]. 2006 Nov [cited 2017 Feb 24];244(5):663–7. Available from:

For peer review only

http://www.ncbi.nlm.nih.gov/pubmed/17060756

36. Dias S, McNamee R, Vail A, S. D, R. M, A. V, et al. Evidence of improving quality of reporting of randomized controlled trials in subfertility. Hum Reprod [Internet]. 2006 Oct [cited 2017 Feb 24];21(10):2617–27. Available from: http://www.ncbi.nlm.nih.gov/pubmed/16793995

37. Smidt N, Rutjes AWS, van der Windt DAWM, Ostelo RWJG, Bossuyt PM, Reitsma JB, et al. The quality of diagnostic accuracy studies since the STARD statement: Has it improved? Neurology [Internet]. 2006 Sep 12 [cited 2018 Mar 12];67(5):792–7. Available from: http://www.ncbi.nlm.nih.gov/pubmed/16966539

38. Coppus SFPJ, van der Veen F, Bossuyt PMM, Mol BWJ, S.F.P.J. C, F. van der V, et al. Quality of reporting of test accuracy studies in reproductive medicine: impact of the Standards for Reporting of Diagnostic Accuracy (STARD) initiative. Fertil Steril [Internet]. 2006 Nov [cited 2017 Jun 26];86(5):1321–9. Available from: http://www.embase.com/search/results?subaction=viewrecord&from=export&id=L44602037

39. Tiruvoipati R, Balasubramanian SP, Atturu G, Peek GJ, Elbourne D. Improving the quality of reporting randomized controlled trials in cardiothoracic surgery: the way forward. J Thorac Cardiovasc Surg [Internet]. 2006 Aug [cited 2017 Jul 21];132(2):233–40. Available from: http://www.ncbi.nlm.nih.gov/pubmed/16872940

40. Mahoney J, Ellison J. Assessing the quality of glucose monitor studies: a critical evaluation of published reports. Clin Chem [Internet]. 2007 Jun 19 [cited 2017 Nov 9];53(6):1122–8. Available from: http://www.clinchem.org/cgi/doi/10.1373/clinchem.2006.083493

41. Poolman RW, Abouali JAK, Conter HJ, Bhandari M. Overlapping systematic reviews of anterior cruciate ligament reconstruction comparing hamstring autograft with bone-patellar tendon-bone autograft: why are they different? J

For peer review only

For peer review only - http://bmjopen.bmj.com/site/about/guidelines.xhtml

Bone Joint Surg Am [Internet]. 2007 Jul 1 [cited 2017 Nov 9];89(7):1542–52. Available from: http://jbjs.org/cgi/doi/10.2106/JBJS.F.01292

42. Spring B, Pagoto S, Knatterud G, Kozak A, Hedeker D. Examination of the analytic quality of behavioral health randomized clinical trials. J Clin Psychol [Internet]. 2007 Jan [cited 2017 Nov 9];63(1):53–71. Available from: http://www.ncbi.nlm.nih.gov/pubmed/17115429

43. Agha R, Cooper D, Muir G. The reporting quality of randomised controlled trials in surgery: A systematic review. Int J Surg [Internet]. 2007 Dec [cited 2017 Nov 9];5(6):413–22. Available from: http://www.ncbi.nlm.nih.gov/pubmed/18029237

44. Kane RL, Wang J, Garrard J. Reporting in randomized clinical trials improved after adoption of the CONSORT statement. J Clin Epidemiol [Internet]. 2007 Mar [cited 2017 Nov 9];60(3):241–9. Available from: http://linkinghub.elsevier.com/retrieve/pii/S0895435606002575

45. Paranjothy B, Shunmugam M, Azuara-Blanco A, B. P, M. S, A. A-B, et al. The quality of reporting of diagnostic accuracy studies in glaucoma using scanning laser polarimetry. J Glaucoma [Internet]. 2007 Dec [cited 2017 Feb 24];16(8):670–5. Available from: http://www.ncbi.nlm.nih.gov/pubmed/18091453

46. Johnson ZK, Siddiqui MAR, Azuara-Blanco A, Z.K. J, M.A.R. S, A. A-B, et al. The Quality of Reporting of Diagnostic Accuracy Studies of Optical Coherence Tomography in Glaucoma. Ophthalmology [Internet]. 2007 Sep [cited 2017 Feb 24];114(9):1607–12. Available from: http://www.ncbi.nlm.nih.gov/pubmed/17434589

47. Riveros C, Dechartres A, Perrodeau E, Haneef R, Boutron I, Ravaud P. Timing and Completeness of Trial Results Posted at ClinicalTrials.gov and Published in Journals. Dickersin K, editor. PLoS Med [Internet]. 2013 Dec 3 [cited 2018 Nov 19];10(12):e1001566. Available from:

For peer review only

https://dx.plos.org/10.1371/journal.pmed.1001566

48. Hind D, Booth A. Do health technology assessments comply with QUOROM diagram guidance? An empirical study. BMC Med Res Methodol [Internet]. 2007 Nov 20 [cited 2017 Jul 17];7:49. Available from: http://www.ncbi.nlm.nih.gov/pubmed/18021461

49. Lee PE, Fischer HD, Rochon PA, Gill SS, Herrmann N, Bell CM, et al. Published randomized controlled trials of drug therapy for dementia often lack complete data on harm. J Clin Epidemiol [Internet]. 2008 Nov [cited 2017 Nov 22];61(11):1152–60. Available from: http://linkinghub.elsevier.com/retrieve/pii/S0895435607004829

50. Pat K, Dooms C, Vansteenkiste J, K. P, C. D, J. V, et al. Systematic review of symptom control and quality of life in studies on chemotherapy for advanced non-small cell lung cancer: How CONSORTed are the data? Lung Cancer [Internet]. 2008 Oct [cited 2017 Mar 10];62(1):126–38. Available from: http://linkinghub.elsevier.com/retrieve/pii/S0169500208001062

51. Folkes A, Urquhart R, Grunfeld E, A. F, R. U, E. G. Are leading medical journals following their own policies on CONSORT reporting? Contemp Clin Trials [Internet]. 2008 Nov [cited 2017 Jun 26];29(6):843–6. Available from: http://linkinghub.elsevier.com/retrieve/pii/S1551714408000906

52. Sinha S, Sinha S, Ashby E, Jayaram R, Grocott MPW. Quality of reporting in randomized trials published in high-quality surgical journals. J Am Coll Surg [Internet]. 2009 Nov [cited 2017 Jul 17];209(5):565–571.e1. Available from: http://linkinghub.elsevier.com/retrieve/pii/S107275150901151X

53. Freeman K, Szczepura A, Osipenko L, K. F, A. S, L. O, et al. Non-invasive fetal RHD genotyping tests: a systematic review of the quality of reporting of diagnostic accuracy in published studies. Eur J Obstet Gynecol Reprod Biol [Internet]. 2009 Feb [cited 2017 Jun 26];142(2):91–8. Available from: http://linkinghub.elsevier.com/retrieve/pii/S0301211508003916

For peer review only

54. Uetani K, Nakayama T, Ikai H, Yonemoto N, Moher D, K. U, et al. Quality of reports on randomized controlled trials conducted in Japan: Evaluation of adherence to the CONSORT statement. Intern Med [Internet]. 2009 [cited 2017 Feb 24];48(5):307–13. Available from: http://www.ncbi.nlm.nih.gov/pubmed/19252352

55. Ethgen M, Boutron L, Steg PG, Roy C, Ravaud P, M. E, et al. Quality of reporting internal and external validity data from randomized controlled trials evaluating stents for percutaneous coronary intervention. BMC Med Res Methodol [Internet]. 2009 Apr 9 [cited 2017 Feb 24];9(1):24. Available from: http://www.embase.com/search/results?subaction=viewrecord&from=export&id=L354667428

56. Krzych LJ, Liszka L. No improvement in studies reporting the diagnostic accuracy of B-type natriuretic peptide. Med Sci Monit [Internet]. 2009 May [cited 2017 Nov 9];15(5):SR5-14. Available from: http://www.ncbi.nlm.nih.gov/pubmed/19396053

57. Pagoto SL, Kozak AT, John P, Bodenlos JS, Hedeker D, Spring B, et al. Intention-to-treat analyses in behavioral medicine randomized clinical trials. Int J Behav Med [Internet]. 2009 Dec 25 [cited 2017 Feb 24];16(4):316–22. Available from: http://link.springer.com/10.1007/s12529-009-9039-3

58. Kidwell CS, Liebeskind DS, Starkman S, Saver JL. Trends in acute ischemic stroke trials through the 20th century. Stroke [Internet]. 2001 Jun [cited 2017 Nov 9];32(6):1349–59. Available from: http://www.ncbi.nlm.nih.gov/pubmed/11387498

59. Han C, Kwak K, Marks DM, Pae C-U, Wu L-T, Bhatia KS, et al. The impact of the CONSORT statement on reporting of randomized clinical trials in psychiatry. Contemp Clin Trials [Internet]. 2009 Mar [cited 2017 Jun 26];30(2):116–22. Available from: http://linkinghub.elsevier.com/retrieve/pii/S1551714408001511

60. Alvarez F, Meyer N, Gourraud PA, Paul C, F. A, N. M, et al. CONSORT adoption

For peer review only

and quality of reporting of randomized controlled trials: a systematic analysis in two dermatology journals. Br J Dermatol [Internet]. 2009 Nov [cited 2017 Feb 23];161(5):1159–65. Available from: http://www.embase.com/search/results?subaction=viewrecord&from=export&id=L355782314

61. Wei X, Tiejun L, Cheng W. Current situation on the reporting quality of randomized controlled trials in 5 leading Chinese medical journals. J Med Coll PLA [Internet]. 2009 Apr [cited 2018 Mar 12];24(2):105–11. Available from: http://linkinghub.elsevier.com/retrieve/pii/S1000194809600253

62. Ladd BO, McCrady BS, Manuel JK, Campbell W, B.O. L, B.S. M, et al. Improving the quality of reporting alcohol outcome studies: Effects of the CONSORT statement. Addict Behav [Internet]. 2010 Jul [cited 2017 Feb 24];35(7):660–6. Available from: http://linkinghub.elsevier.com/retrieve/pii/S0306460310000560

63. Yu L-M, Chan A-W, Hopewell S, Deeks JJ, Altman DG. Reporting on covariate adjustment in randomised controlled trials before and after revision of the 2001 CONSORT statement: a literature review. Trials [Internet]. 2010 May 18 [cited 2017 Nov 9];11(1):59. Available from: http://trialsjournal.biomedcentral.com/articles/10.1186/1745-6215-11-59

64. Areia M, Soares M, Dinis-Ribeiro M. Quality reporting of endoscopic diagnostic studies in gastrointestinal journals: where do we stand on the use of the STARD and CONSORT statements? Endoscopy [Internet]. 2010 Feb 5 [cited 2017 Nov 9];42(2):138–47. Available from: http://www.thieme-connect.de/DOI/DOI?10.1055/s-0029-1243846

65. Delaney M, Meyer E, Cserti-Gazdewich C, Haspel RL, Lin Y, Morris A, et al. A systematic assessment of the quality of reporting for platelet transfusion studies. Transfusion [Internet]. 2010 Oct [cited 2017 Jun 26];50(10):2135–44. Available from: http://doi.wiley.com/10.1111/j.1537-2995.2010.02691.x

66. Flint HE, Harrison JE. How well do reports of clinical trials in the orthodontic

For peer review only

literature comply with the CONSORT statement? J Orthod [Internet]. 2010 Dec 16 [cited 2017 Feb 24];37(4):250–61. Available from: http://www.tandfonline.com/doi/full/10.1179/14653121043191

67. Hopewell S, Dutton S, Yu L-M, Chan A-W, Altman DG, S. H, et al. The quality of reports of randomised trials in 2000 and 2006: comparative study of articles indexed in PubMed. BMJ [Internet]. 2010 Mar 23 [cited 2017 Jun 26];340(mar23 1):c723. Available from: http://www.embase.com/search/results?subaction=viewrecord&from=export&id=L358557565

68. Ernst E, Hung SK CY. NCCAM-funded RCTs of herbal medicines: An independent, critical assessment. Perfusion [Internet]. 2011;24:89–102. Available from: https://www.researchgate.net/publication/288438592_NCCAM-funded_RCTs_of_herbal_medicines_An_independent_critical_assessment

69. Sánchez-Thorin JC, Cortés MC, Montenegro M, Villate N, J.C. S-T, M.C. C, et al. The quality of reporting of randomized clinical trials published in Ophthalmology. Ophthalmology [Internet]. 2001 Feb [cited 2017 Feb 24];108(2):410–5. Available from: http://www.embase.com/search/results?subaction=viewrecord&from=export&id=L32115976

70. Selman TJ, Morris RK, Zamora J, Khan KS. The quality of reporting of primary test accuracy studies in obstetrics and gynaecology: application of the STARD criteria. BMC Womens Health [Internet]. 2011 Mar 23 [cited 2017 Jul 17];11(1):8. Available from: http://bmcwomenshealth.biomedcentral.com/articles/10.1186/1472-6874-11-8

71. Parsons NR, Hiskens R, Price CL, Achten J, Costa ML, N.R. P, et al. A systematic survey of the quality of research reporting in general orthopaedic journals. J Bone Jt Surg - Ser B [Internet]. 2011 Sep 1 [cited 2017 Feb 24];93 B(9):1154–9. Available from: http://www.bjj.boneandjoint.org.uk/cgi/doi/10.1302/0301-620X.93B9.27193

For peer review only

72. Kiehna EN, Starke RM, Pouratian N, Dumont AS. Standards for reporting randomized controlled trials in neurosurgery. J Neurosurg [Internet]. 2011 Feb [cited 2017 Feb 24];114(2):280–5. Available from: http://thejns.org/doi/10.3171/2010.8.JNS091770

73. Strech D, Soltmann B, Weikert B, Bauer M, Pfennig A, D. S, et al. Quality of reporting of randomized controlled trials of pharmacologic treatment of bipolar disorders: A systematic review. J Clin Psychiatry [Internet]. 2011 Sep 15 [cited 2017 Feb 24];72(9):1214–21. Available from: http://article.psychiatrist.com/?ContentType=START&ID=10007267

74. Turner L-A, Singh K, Garritty C, Tsertsvadze A, Manheimer E, Wieland LS, et al. An evaluation of the completeness of safety reporting in reports of complementary and alternative medicine trials. BMC Complement Altern Med [Internet]. 2011 Aug 22 [cited 2017 Jun 26];11(1):67. Available from: http://bmccomplementalternmed.biomedcentral.com/articles/10.1186/1472-6882-11-67

75. Haidich A-B, Birtsou C, Dardavessis T, Tirodimos I, Arvanitidou M, A.-B. H, et al. The quality of safety reporting in trials is still suboptimal: survey of major general medical journals. J Clin Epidemiol [Internet]. 2011 Feb [cited 2017 Feb 24];64(2):124–35. Available from: http://linkinghub.elsevier.com/retrieve/pii/S0895435610001083

76. Gray R, Sullivan M, Altman DG, Gordon-Weeks AN, R. G, M. S, et al. Adherence of trials of operative intervention to the CONSORT statement extension for nonpharmacological treatments: A comparative before and after study. Ann R Coll Surg Engl [Internet]. 2012 Sep 1 [cited 2017 Feb 24];94(6):388–94. Available from: http://openurl.ingenta.com/content/xref?genre=article&issn=0035-8843&volume=94&issue=6&spage=388

77. Cornelius VR, Sauzet O, Williams JE, Ayis S, Farquhar-Smith P, Ross JR, et al. Adverse event reporting in randomised controlled trials of neuropathic pain: considerations for future practice. Pain [Internet]. 2013 Feb [cited 2017 Nov

For peer review only

22];154(2):213–20. Available from: http://content.wkhealth.com/linkback/openurl?sid=WKPTLP:landingpage&an=00006396-201302000-00009

78. Diaz-Ordaz K, Froud R, Sheehan B, Eldridge S. A systematic review of cluster randomised trials in residential facilities for older people suggests how to improve quality. BMC Med Res Methodol [Internet]. 2013 Oct 22 [cited 2017 Jul 17];13(1):127. Available from: http://bmcmedresmethodol.biomedcentral.com/articles/10.1186/1471-2288-13-127

79. Geha NN, Moseley AM, Elkins MR, Chiavegato LD, Shiwa SR, Costa LOP. The quality and reporting of randomized trials in cardiothoracic physical therapy could be substantially improved. Respir Care [Internet]. 2013 Nov;58(11):1899–906. Available from: http://rc.rcjournal.com/cgi/doi/10.4187/respcare.02379

80. Moher D, Jones A, Lepage L, CONSORT Group (Consolidated Standards for Reporting of Trials). Use of the CONSORT statement and quality of reports of randomized trials: a comparative before-and-after evaluation. JAMA [Internet]. 2001 Apr 18 [cited 2017 Jun 7];285(15):1992–5. Available from: http://www.ncbi.nlm.nih.gov/pubmed/11308436

81. Liu LQ, Morris PJ, Pengel LHM, L.Q. L, P.J. M, L.H.M. P, et al. Compliance to the CONSORT statement of randomized controlled trials in solid organ transplantation: A 3-year overview. Transpl Int [Internet]. 2013 Mar [cited 2017 Feb 24];26(3):300–6. Available from: http://www.embase.com/search/results?subaction=viewrecord&from=export&id=L52379666

82. Panic N, Leoncini E, de Belvis G, Ricciardi W, Boccia S, N. P, et al. Evaluation of the endorsement of the preferred reporting items for systematic reviews and meta-analysis (PRISMA) statement on the quality of published systematic review and meta-analyses. Derrick GE, editor. PLoS One [Internet]. 2013 Dec 26 [cited 2017 Jan 15];8(12):e83138. Available from:

For peer review only

http://www.embase.com/search/results?subaction=viewrecord&from=export&id=L372005920

83. Fleming PS, Seehra J, Polychronopoulou A, Fedorowicz Z, Pandis N. A PRISMA assessment of the reporting quality of systematic reviews in orthodontics. Angle Orthod [Internet]. 2013 Jan [cited 2017 Nov 9];83(1):158–63. Available from: http://www.ncbi.nlm.nih.gov/pubmed/22720835

84. Péron J, Maillet D, Gan HK, Chen EX, You B, J. P, et al. Adherence to CONSORT adverse event reporting guidelines in randomized clinical trials evaluating systemic cancer therapy: A systematic review. J Clin Oncol [Internet]. 2013 Nov 1 [cited 2017 Feb 24];31(31):3957–63. Available from: http://ascopubs.org/doi/10.1200/JCO.2013.49.3981

85. Tunis AS, McInnes MDF, Hanna R, Esmail K. Association of Study Quality with Completeness of Reporting: Have Completeness of Reporting and Quality of Systematic Reviews and Meta-Analyses in Major Radiology Journals Changed Since Publication of the PRISMA Statement? Radiology [Internet]. 2013 Nov [cited 2018 Mar 12];269(2):413–26. Available from: http://pubs.rsna.org/doi/10.1148/radiol.13130273

86. Maclean EN, Stone IS, Ceelen F, Garcia-Albeniz X, Sommer WH, Petersen SE. Quality of reporting in cardiac MRI, CT and SPECT diagnostic accuracy studies: Analysis of the impact of STARD criteria. J Cardiovasc Magn Reson [Internet]. 2013 Jun 1 [cited 2017 Feb 24];15(6):61. Available from: http://www.embase.com/search/results?subaction=viewrecord&from=export&id=L70993941

87. Baker D, Lidster K, Sottomayor A, Amor S. Two Years Later: Journals Are Not Yet Enforcing the ARRIVE Guidelines on Reporting Standards for Pre-Clinical Animal Studies. Eisen JA, editor. PLoS Biol [Internet]. 2014 Jan 7 [cited 2017 Sep 29];12(1):e1001756. Available from: http://www.ncbi.nlm.nih.gov/pubmed/24409096

For peer review only

88. Choi J, Jun JH, Kang BK, Kim KH, Lee MS, J. C, et al. Endorsement for improving the quality of reports on randomized controlled trials of traditional medicine journals in Korea: a systematic review. Trials [Internet]. 2014 Nov 5 [cited 2017 Jan 15];15(1):429. Available from: http://trialsjournal.biomedcentral.com/articles/10.1186/1745-6215-15-429

89. Walther S, Schueler S, Tackmann R, Schuetz GM, Schlattmann P, Dewey M, et al. Compliance with STARD checklist among studies of coronary CT angiography: systematic review. Radiology [Internet]. 2014 Apr [cited 2017 Feb 23];271(1):74–86. Available from: http://pubs.rsna.org/doi/10.1148/radiol.13121720

90. Ghimire S, Kyung E, Lee H, Kim E. Oncology trial abstracts showed suboptimal improvement in reporting: a comparative before-and-after evaluation using CONSORT for Abstract guidelines. J Clin Epidemiol [Internet]. 2014 Jun [cited 2017 Feb 24];67(6):658–66. Available from: http://linkinghub.elsevier.com/retrieve/pii/S0895435613004435

91. Devereaux PJ, Manns BJ, Ghali WA, Quan H, Guyatt GH, P.J. D, et al. The reporting of methodological factors in randomized controlled trials and the association with a journal policy to promote adherence to the Consolidated Standards of Reporting Trials (CONSORT) checklist. Control Clin Trials [Internet]. 2002 Aug [cited 2017 Feb 24];23(4):380–8. Available from: http://www.embase.com/search/results?subaction=viewrecord&from=export&id=L34874890

92. Song T-J, Leng H-F, Zhong LL, Wu T-X, Bian Z-X. CONSORT in China: past development and future direction. Trials [Internet]. 2015 Jun 1 [cited 2017 Jun 26];16(1):243. Available from: http://trialsjournal.biomedcentral.com/articles/10.1186/s13063-015-0769-z

93. Stevely A, Dimairo M, Todd S, Julious SA, Nicholl J, Hind D, et al. An Investigation of the Shortcomings of the CONSORT 2010 Statement for the Reporting of Group Sequential Randomised Controlled Trials: A Methodological Systematic

For peer review only

939 Review. Shamji M, editor. PLoS One [Internet]. 2015 Nov 3 [cited 2017 Feb 24];10(11):e0141104. Available from: http://dx.plos.org/10.1371/journal.pone.0141104

942 94. Adie S, Ma D, Harris IA, Naylor JM, Craig JC, S. A, et al. Quality of conduct and reporting of meta-analyses of surgical interventions. Ann Surg [Internet]. 2015 Apr [cited 2017 Jun 26];261(4):685–94. Available from: http://content.wkhealth.com/linkback/openurl?sid=WKPTLP:landingpage%7B&%7Dan=00000658-201504000-00012

947 95. Jull A, Aye PS. Endorsement of the CONSORT guidelines, trial registration, and the quality of reporting randomised controlled trials in leading nursing journals: A cross-sectional analysis. Int J Nurs Stud [Internet]. 2015 Jun [cited 2017 Feb 10];52(6):1071–9. Available from: http://linkinghub.elsevier.com/retrieve/pii/S0020748914003022

952 96. Bearn DR, Alharbi F. Reporting of clinical trials in the orthodontic literature from 2008 to 2012: observational study of published reports in four major journals. J Orthod [Internet]. 2015 Sep 15 [cited 2017 Feb 24];42(3):186–91. Available from: http://www.tandfonline.com/doi/full/10.1179/1465313315Y.0000000011

956 97. Agha RA, Fowler AJ, Limb C, Whitehurst K, Coe R, Sagoo H, et al. Impact of the mandatory implementation of reporting guidelines on reporting quality in a surgical journal: A before and after study. Int J Surg [Internet]. 2016 Jun [cited 2017 Feb 20];30:169–72. Available from: http://linkinghub.elsevier.com/retrieve/pii/S1743919116300620

961 98. Pouwels KB, Widyakusuma NN, Groenwold RHH, Hak E. Quality of reporting of confounding remained suboptimal after the STROBE guideline. J Clin Epidemiol [Internet]. 2016 Jan [cited 2017 Feb 24];69:217–24. Available from: http://linkinghub.elsevier.com/retrieve/pii/S0895435615003856

965 99. Rao A, Brück K, Methven S, Evans R, Stel VS, Jager KJ, et al. Quality of reporting and study design of CKD cohort studies assessing mortality in the elderly before

For peer review only

and after STROBE: A systematic review. Zhou X, editor. PLoS One [Internet]. 2016 May 11 [cited 2017 Jun 26];11(5):e0155078. Available from: http://dx.plos.org/10.1371/journal.pone.0155078

100. Grob ATM, van der Vaart LR, Withagen MIJ, van der Vaart CH. The quality of reporting of diagnostic accuracy studies in pelvic floor transperineal three-dimensional ultrasound: a systematic review. Ultrasound Obstet Gynecol [Internet]. 2016 Dec 21 [cited 2017 Mar 10]; Available from: http://doi.wiley.com/10.1002/uog.17390

101. Rikos D, Dardiotis E, Tsivgoulis G, Zintzaras E, Hadjigeorgiou GM, D. R, et al. Reporting quality of randomized-controlled trials in multiple sclerosis from 2000 to 2015, based on CONSORT statement. Mult Scler Relat Disord [Internet]. 2016 Sep [cited 2017 Feb 24];9:135–9. Available from: http://linkinghub.elsevier.com/retrieve/pii/S2211034816301225

102. Montori VM, Bhandari M, Devereaux PJ, Manns BJ, Ghali WA, Guyatt GH. In the dark: the reporting of blinding status in randomized controlled trials. J Clin Epidemiol [Internet]. 2002 Aug [cited 2017 Nov 9];55(8):787–90. Available from: http://www.ncbi.nlm.nih.gov/pubmed/12384193

103. Bigna JJR, Um LN, Nansseu JRN, J.J.R. B, L.N. U, J.R.N. N. A comparison of quality of abstracts of systematic reviews including meta-analysis of randomized controlled trials in high-impact general medicine journals before and after the publication of PRISMA extension for abstracts: A systematic review and m. Syst Rev [Internet]. 2016 Oct 13 [cited 2017 Jun 26];5(1):174. Available from: http://www.embase.com/search/results?subaction=viewrecord&from=export&id=L612833923

104. Sarkis-Onofre R, Poletto-Neto V, Cenci MS, Pereira-Cenci T, Moher D. Impact of the CONSORT Statement endorsement in the completeness of reporting of randomized clinical trials in restorative dentistry. J Dent [Internet]. 2017 Mar 31 [cited 2017 Feb 24];58:54–9. Available from: http://linkinghub.elsevier.com/retrieve/pii/S0300571217300283

For peer review only

105. Tharyan P, Premkumar TS, Mathew V, Barnabas JP, Manuelraj. Editorial policy and the reporting of randomized controlled trials: a survey of instructions for authors and assessment of trial reports in Indian medical journals (2004-05). Natl Med J India [Internet]. [cited 2017 Nov 9];21(2):62–8. Available from: http://www.ncbi.nlm.nih.gov/pubmed/18807310

106. Lai TYY, Wong VWY, Lam RF, Cheng ACO, Lam DSC, Leung GM. Quality of reporting of key methodological items of randomized controlled trials in clinical ophthalmic journals. Ophthalmic Epidemiol [Internet]. 14(6):390–8. Available from: http://www.ncbi.nlm.nih.gov/pubmed/18161613

107. Cobo E, Selva-O'Callagham A, Ribera JM, Cardellach F, Dominguez R, Vilardell M. Statistical reviewers improve reporting in biomedical articles: A randomized trial. PLoS One. 2007;2(3).

108. Implementing Reporting Guidelines: Why and How, for Journal Editors [Internet]. World Association of Medical Editors. Available from: https://wame.blog/2017/09/17/implementing-reporting-guidelines-why-and-how-for-journal-editors/

109. Rupinski M, Zagorowicz E, Regula J, Fijuth J, Kraszewska E, Polkowski M, et al. Randomized Comparison of Three Palliative Regimens Including Brachytherapy, Photodynamic Therapy and APC in Patients With Malignant Dysphagia (CONSORT 1a) (Revised II). Am J Gastroenterol [Internet]. 2011 Sep 14 [cited 2018 Mar 4];106(9):1612–20. Available from: http://www.nature.com/articles/ajg2011178

110. Jilka RL. The Road to Reproducibility in Animal Research. J Bone Miner Res [Internet]. 2016 Jul [cited 2017 Jul 21];31(7):1317–9. Available from: http://doi.wiley.com/10.1002/jbmr.2881

111. Moher D, Altman DG, Roberts I, Dirnagl U, Chalmers I, Berlin J. Four Proposals to Help Improve the Medical Research Literature. PLOS Med [Internet]. 2015 Sep 22 [cited 2017 Aug 29];12(9):e1001864. Available from:

For peer review only

http://dx.plos.org/10.1371/journal.pmed.1001864

112. Pandis N, Turpin DL, N. P, D.L. T, Pandis N, Turpin DL. Enhancing CONSORT compliance for improved reporting of randomized controlled trials. Am J Orthod Dentofac Orthop [Internet]. 2014 Jan [cited 2017 Jul 21];145(1):1. Available from: http://www.embase.com/search/results?subaction=viewrecord&from=export&id=L372038916

113. Hill CL, LaValley MP, Felson DT. Secular changes in the quality of published randomized clinical trials in rheumatology. Arthritis Rheum [Internet]. 2002 Mar [cited 2017 Nov 9];46(3):779–84. Available from: http://doi.wiley.com/10.1002/art.512

114. Hutton B, Salanti G, Caldwell DM, Chaimani A, Schmid CH, Cameron C, et al. The PRISMA extension statement for reporting of systematic reviews incorporating network meta-analyses of health care interventions: checklist and explanations. Ann Intern Med [Internet]. 2015 Jun 2 [cited 2017 Jun 26];162(11):777–84. Available from: http://annals.org/article.aspx?doi=10.7326/M14-2385

115. Hirst A, Altman DG. Are peer reviewers encouraged to use reporting guidelines? A survey of 116 health research journals. Cameron DW, editor. PLoS One [Internet]. 2012 Apr 27 [cited 2017 Jun 7];7(4):e35621. Available from: http://dx.plos.org/10.1371/journal.pone.0035621

116. ClinicalTrials.gov. National Library of Medicine (US). Available from: https://clinicaltrials.gov/

117. Pandis N, Shamseer L, Kokich VG, Fleming PS, Moher D, N. P, et al. Active implementation strategy of CONSORT adherence by a dental specialty journal improved randomized clinical trial reporting. J Clin Epidemiol [Internet]. 2014 Sep [cited 2017 Jan 15];67(9):1044–8. Available from: http://linkinghub.elsevier.com/retrieve/pii/S0895435614001140

118. Cobo E, Cortes J, Ribera JM, Cardellach F, Selva-O'Callaghan A, Kostov B, et al.

For peer review only

For peer review only - http://bmjopen.bmj.com/site/about/guidelines.xhtml

Effect of using reporting guidelines during peer review on quality of final manuscripts submitted to a biomedical journal: masked randomised trial. 2011 Nov;343(nov22 2). Available from: http://www.ncbi.nlm.nih.gov/pubmed/22108262

119. Hopewell S, Boutron I, Altman DG, Barbour G, Moher D, Montori V, et al. Impact of a web-based tool (WebCONSORT) to improve the reporting of randomised trials: results of a randomised controlled trial. BMC Med [Internet]. 2016 Nov 28 [cited 2017 Feb 24];14(1):199. Available from: http://www.ncbi.nlm.nih.gov/pubmed/27894295

120. Hopewell S, Collins GS, Boutron I, Yu L-M, Cook J, Shanyinde M, et al. Impact of peer review on reports of randomised trials published in open peer review journals: retrospective before and after study. BMJ [Internet]. 2014 [cited 2017 Jun 7];349. Available from: http://www.bmj.com/content/349/bmj.g4145

121. A peerless review? Automating methodological and statistical review. Available from: https://blogs.biomedcentral.com/bmcblog/2016/05/23/peerless-review-automating-methodological-statistical-review/

122. Mbuagbaw L, Thabane M, Vanniyasingam T, Borg Debono V, Kosa S, Zhang S, et al. Improvement in the quality of abstracts in major clinical journals since CONSORT extension for abstracts: a systematic review. Contemp Clin Trials [Internet]. 2014 Jul [cited 2017 Feb 24];38(2):245–50. Available from: http://linkinghub.elsevier.com/retrieve/pii/S1551714414000809

123. Schriger DL, Altman DG. Inadequate post-publication review of medical research. BMJ [Internet]. 2010 Aug 11 [cited 2018 Mar 4];341:c3803. Available from: http://www.ncbi.nlm.nih.gov/pubmed/20702543

124. Statreviewer. Available from: http://www.statreviewer.com/

125. Tools and templates for implementing reporting guidelines. Available from: http://www.equator-network.org/toolkits/using-guidelines-in-journals/tools-and-templates-for-implementing-reporting-guidelines/

For peer review only

126. PubPeer. Available from: https://pubpeer.com/

127. ScienceOpen. Available from: https://www.scienceopen.com/

128. Péron J, You B, Gan HK, Maillet D, Chen EX, Pond GR, et al. Influence of statistician involvement on reporting of randomized clinical trials in medical oncology. Anticancer Drugs [Internet]. 2013 Mar [cited 2017 Feb 24];24(3):306–9. Available from: http://content.wkhealth.com/linkback/openurl?sid=WKPTLP:landingpage&an=00001813-201303000-00010

129. Pandis N, Polychronopoulou A, Eliades T. An assessment of quality characteristics of randomised control trials published in dental journals. J Dent [Internet]. 2010 Sep [cited 2017 Feb 24];38(9):713–21. Available from: http://linkinghub.elsevier.com/retrieve/pii/S0300571210001235

130. Kloukos D, Papageorgiou SN, Doulis I, Petridis H, Pandis N. Reporting quality of randomised controlled trials published in prosthodontic and implantology journals. J Oral Rehabil [Internet]. 2015 Dec [cited 2017 Feb 24];42(12):914–25. Available from: http://doi.wiley.com/10.1111/joor.12325

131. Blanco D, Biggane AM, Cobo E. Are CONSORT checklists submitted by authors adequately reflecting what information is actually reported in published papers? Trials [Internet]. 2018 Dec 29 [cited 2018 Jan 31];19(1):80. Available from: https://trialsjournal.biomedcentral.com/articles/10.1186/s13063-018-2475-0

132. Koletsi D, Fleming PS, Behrents RG, Lynch CD, Pandis N. The use of tailored subheadings was successful in enhancing compliance with CONSORT in a dental journal. J Dent [Internet]. 2017 Dec [cited 2018 Mar 23];67:66–71. Available from: http://www.ncbi.nlm.nih.gov/pubmed/28941813

133. Cobo E, González JA. Taking advantage of unexpected WebCONSORT results. BMC Med [Internet]. 2016 Dec 5 [cited 2018 Feb 16];14(1):204. Available from: http://bmcmedicine.biomedcentral.com/articles/10.1186/s12916-016-0758-4

For peer review only

134. Tijdink JK, Schipper K, Bouter LM, Maclaine Pont P, de Jonge J, Smulders YM. How do scientists perceive the current publication culture? A qualitative focus group interview study among Dutch biomedical researchers. BMJ Open [Internet]. 2016 Feb 17 [cited 2017 Sep 28];6(2):e008681. Available from: http://bmjopen.bmj.com/lookup/doi/10.1136/bmjopen-2015-008681

Group	Intervention	Rationale
**Training on the practical use of RGs**	Introduction of RGs & journalology into graduate curricula (18-22)	To introduce good research reporting habits early in young researchers' scientific careers.
	Student's development of protocols for coursework and research using RGs (21)	
	Funder's support of author training on RGs (23)	Authors, editors, and peer reviewers have insufficient training in issues related to reporting.
	Training for peer reviewers and editors	

For peer review only

Figures, tables and supplementary files

Figure 1: PRISMA flow diagram.

Figure 2: Typology of interventions to improve adherence to RGs according to type of intervention and research stage. **Legend:** Evaluated interventions are shown in bold.

Figure 3: Gaps in research on the evaluation of interventions to improve adherence to reporting guidelines. **Legend:** Each circle represents one intervention. Variables displayed: 1) Circle size: Number of studies evaluating each intervention (bigger = more studies); 2) Circle colour: Study design of those studies (blue for RCTs and green for observational studies) and 3) Circle fill: Kind of RG implementation (plain for checklist and stripes for bullet points and examples). Research gaps are highlighted in red.

Supplementary file 1: Description of the acronyms and full names of all reporting guidelines considered.

Supplementary file 2: implementation details of the evaluated interventions.

Table 1: Rationale of the interventions identified.

	on RGs by journals (22,23)	
**Enhancing accessibility and understanding**	Dissemination of RGs by scientific associations (24)	A large number of researchers are not aware of the existence of RGs.
	Translation of RGs to further languages (25)	Language barriers may affect the proper use of RGs.
	Development of expanded database of examples for each RG (26)	Authors need more examples of good reporting to properly understand certain items.
**Encouraging adherence**	Author use of RGs as a template for grant application proposals (21)	Using RGs in early stages may facilitate completeness of reporting of published research.
	Required checklist for ethics approval application (11)	
	Funder's requirement of checklists in author's report (21,108)	
	Author use of the writing aid tool COBWEB (12)	A) Authors need help to successfully adhere to RGs at the writing stage and B) Dividing RG items into bullet points and providing examples might help.
	Author use of a structured approach for reporting research (47,112)	A) To help authors avoid omissions, B) to aid reviewers and editors in appraising articles and C) to allow more efficient data extraction during the systematic review process.
	Author markup of the manuscript to indicate where each RG item is addressed (109)	
	Editorial statement endorsing certain RGs (27–46,48–106,113)	Authors read editorial statements and follow “Instructions to authors”.
	Recommendation or requirement to follow RGs in the "Instructions to authors“ (27–46,48–106,113)	
	Requirement to submit a RG checklist together with the manuscript indicating page numbers corresponding to each item (27–46,48–106,113)	Authors may not consider editorial statements or recommendations in “Instructions to authors” to be important. Compulsory submission of checklists or text mark-up may encourage authors to be more compliant with RGs.
	Requirement to populate and submit a RG checklist with text from the manuscript (114)	

	Journal development of core versions of RGs containing key items (110)	Focusing on the most important items could be more effective than considering the whole checklist.
	Guidance to authors on manuscript preparation by publication officers (111)	Trained journal officers may enhance authors' compliance with RGs during manuscript preparation.
	Suggestion for peer reviewers to use RGs (107)	Peer reviewers often do not detect reporting flaws. Therefore, they may need to follow a more systematic approach and use RGs.
	Editor's questions to peer reviewers about whether the authors have followed RGs (115)	
**Checking adherence and providing feedback**	Completeness of reporting check by editors (117)	Requiring checklists at submission does not guarantee adherence. Editors and peer reviewers have to check whether submitted papers are compliant with RGs.
	Peer review against RGs (118)	
	Internal peer review against RGs by a trained editorial assistant (120)	It is extremely unlikely that the average clinical peer reviewer has the methodological expertise to check a paper against RGs.
	Implementation of the automatic tool Statreviewer (121)	
	Email to authors to revise the manuscript according to RGs (13)	It might be more effective to ask authors for adherence to RGs during the revision process because they will do anything to get their paper published.
	Implementation of the tool WebCONSORT (119)	
	Completeness of reporting check at copy-editing (122)	Copy-editing and post-publication offer alternate time points to improve adherence to RGs.
	Post- publication peer review (123)	
**Involvement of experts**	Statistician involvement (78,128-130)	Professionals with specific knowledge of RGs might help authors when designing, conducting or reporting their research.
	Medical writer involvement (108)	

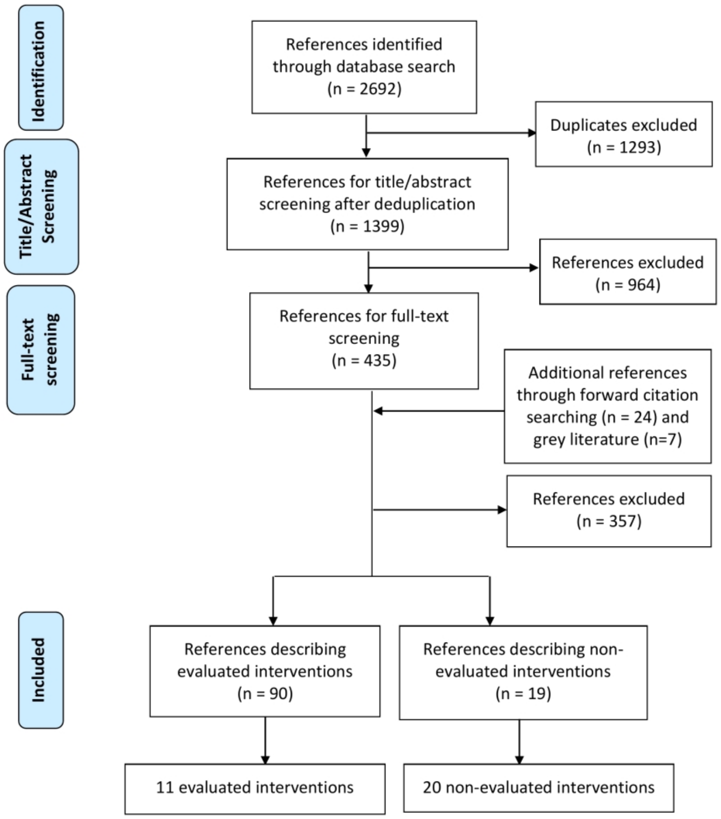

215x279mm (300 x 300 DPI)

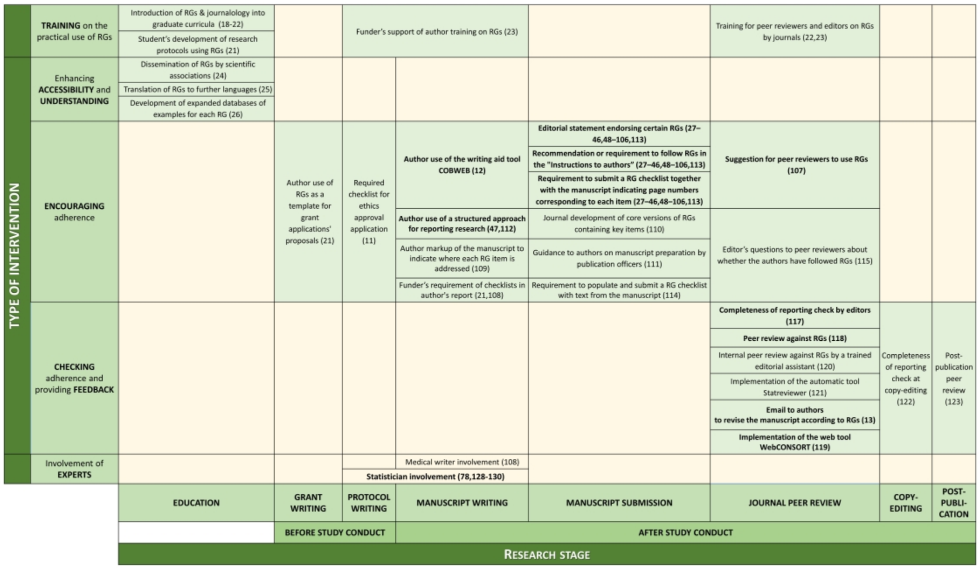

Typology of interventions to improve adherence to RGs according to type of intervention and research stage.
Legend: Evaluated interventions are shown in bold.

670x379mm (300 x 300 DPI)

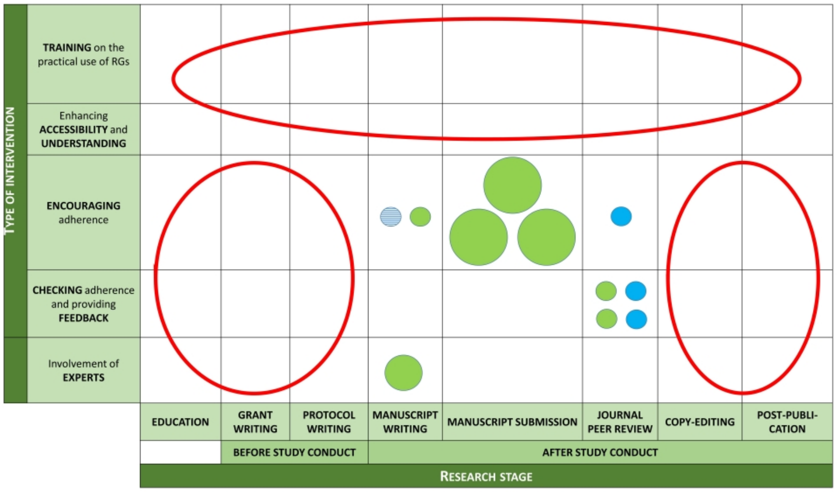

Gaps in research on the evaluation of interventions to improve adherence to reporting guidelines. Legend: Each circle represents one intervention. Variables displayed: 1) Circle size: Number of studies evaluating each intervention (bigger = more studies); 2) Circle colour: Study design of those studies (blue for RCTs and green for observational studies) and 3) Circle fill: Kind of RG implementation (plain for checklist and stripes for bullet points and examples). Research gaps are highlighted in red.

670x379mm (300 x 300 DPI)

Acronym	Full name
CONSORT	Consolidated Standards of Reporting Trials
STROBE	Strengthening the Reporting of Observational Studies in Epidemiology
PRISMA	Preferred Reporting Items for Systematic Reviews and Meta-Analyses
SRQR	Standards for Reporting Qualitative Research
COREQ	Consolidated criteria for Reporting Qualitative research
STARD	Standard Protocol Items: Recommendations for Interventional Trials
TRIPOD	Transparent Reporting of a multivariable prediction model for Individual Prognosis or Diagnosis
SQUIRE	Standards for Quality Improvement Reporting Excellence
CHEERS	Consolidated Health Economic Evaluation Reporting Standards
SPIRIT	Standard Protocol Items: Recommendations for Interventional Trials
PRISMA-P	Preferred Reporting Items for Systematic Review and Meta-Analysis Protocols
CARE	Case Report
AGREE	Appraisal of Guidelines, Research and Evaluation
ARRIVE	Animal Research: Reporting In Vivo Experiments
RIGHT	Reporting Tool for Practice Guidelines in Health Care
QUOROM	Quality of Reporting of Meta-analyses

For peer review only

Type of intervention	Intervention	Number of studies and study design	Details of the intervention	RGs implemented	Format of RG implementation	Measure of adherence to RGs	Effect on adherence to RGs*
Encouraging adherence	Implementation of the writing aid tool COBWEB (12)	1 RCT	Participants have to write the six domains of the methods section of the manuscript for the protocol they receive. They have access to COBWEB tool for a random three of the six domains.	CONSORT & CONSORT extension for non-pharmacological interventions	Bullet points and examples (6 items)	Mean score for completeness of reporting (scale 0–10, items weighted)	Difference of 2.1 (95% CI 1.5-2.7)
	Author use of a structured approach for reporting research (47)	1 Observational study (cross-sectional evaluation)	Results are posted in a standard tabular format without discussions or conclusions.	CONSORT	Checklist (4 items)	Percentage compliance of each RG item	Difference of 0.16, 0.10, 0.18 and 0.36 for each of the 4 items considered
	Journal endorsement (3 interventions, see “Details of the intervention”) (27–46,48–106,113)	80 observational studies (57 cross sectional evaluations of endorsing vs non-endorsing journals, 9 before and after evaluations of endorsing journals before and after endorsement, 14 both kind of evaluations)	A) Editorial statement endorsing certain RGs,<br>B) Recommendation or requirement to follow RGs in the "Instructions to authors", and<br>C) Requirement to submit a RG checklist together with the manuscript indicating page numbers corresponding to each item.	CONSORT (46 of 80)<br>CONSORT extensions (9 of 80)<br>QUOROM (3 of 80)<br>PRISMA (4 of 80)<br>PRISMA extensions (1 of 80)<br>STARD (11 of 80)<br>STROBE (4 of 80)<br>ARRIVE (1 of 80)<br>CONSORT, STROBE and PRISMA (11 of 80)	Checklist (all items)	For CONSORT: percentage of compliance for each item**<br><br>For other RGs: Mean summed score for completeness of reporting**	For CONSORT: 25 items improved (see details for each item on figure 2 on Turner et al. (6))<br><br>For CONSORT extension for harms: Difference of 0.04 (99% CI –1.50 to 1.58) (see Stevens et al. (3))

						For PRISMA: Difference of 0.53 (99% CI 0.02 to 1.03) (see Stevens et al. (3))<br><br>For STARD: Difference of 0.52 (99% CI –0.11 to 1.16) (see Stevens et al. (3))<br><br>For STRICTA: Difference of 1.42 (99% CI –0.04 to 2.88) (see Stevens et al. (3))<br><br>For STROBE: Difference of 1.55 (99% CI –3.19 to 6.29) (see Stevens et al. (3))
Suggestion for peer reviewers to use RGs (107)	1 RCT	Peer reviewers are sent a standard letter encouraging them to use different reporting guidelines. Reviewers are not asked to report whether they used the reporting guideline in reviewing the manuscript.	CONSORT, QUOROM, STARD	Checklist (all items)	Modified version of Manuscript Quality Assessment Instrument (scale 36-180)	Difference of 0.9 (95% CI -0.3 to +2.1)

Checking adherence and providing feedback	Completeness of reporting check by the editors (117)	1 Observational Study (Before and after evaluation)	Initial submissions are vetted by the editor-in-chief. If the submission is considered appropriate, manuscripts are assessed by the associate editor for CONSORT adherence. Authors are asked to make changes accordingly until associate editor deems appropriate that they move to the next step of the review process leading to an editorial decision.	CONSORT	Checklist (all items)	Percentage of compliance of each RG item	Before – compliance ranges from 0% to 100% (Median 40%)<br><br>After – perfect compliance in 33 out of 37 items
	Additional review against RGs (118)	1 RCT	A senior statistician does an additional review of all papers and provides authors suggestions on how to follow reporting guideline checklists.	STROBE, CONSORT, STARD	Checklist (all items)	Modified version of Manuscript Quality Assessment Instrument (scale 1 to 9)	Difference of 0.25 (95% CI<br>-0.05 to +0.54)
	Active implementation of RG by editors (2 interventions, see "Details of the intervention") (13)	1 Observational study (Interrupted time series evaluation)	A) Email is sent to authors to revise the abstract according to the guidelines at the revision stage and B) Changes are made by the assistant editors of these journals towards the end of the editorial process.	CONSORT extension for abstracts	Checklist (9 of 17 items)	Monthly mean number of items reported (scale 0 to 9)	Difference of 1.5 items

	Implementation of the web-based tool WebCONSORT (119)	1 RCT	Journal editor includes a link to WebCONSORT in the revision letter to authors. Authors are directed to an automatically generated list of items and a flow diagram customised to their specific trial design.	CONSORT & some CONSORT extensions	Checklist (10 of 25 items)	Percentage of items reported for each article	Difference of 0.04 (95% CI −0.02 to +0.10)
Involvement of experts	Statistician involvement (78,128-130)	4 Observational studies (cross sectional evaluations)	Statisticians (or epidemiologists or other quantitative methodologists) are involved in the design, conduct or reporting of the study	CONSORT	Checklist (all items)	Mean score for completeness of reporting (scale 0-10, items not weighted)	In Diaz-Ordaz (78): No global effect provided (see effects for individual items in Table 2 of the paper)<br><br>In Pandis et al. (128): Difference of 0.93<br><br>In Péron et al. (129): No difference in medians<br><br>In Kloukos et al. (130): 0.27

*Difference between adherence to RGs in intervention and non-intervention group. We did not report the CI of the effect size when authors did not report it in the original papers.

**As the 80 individual studies that belong to this category used different measures of adherence to reporting guidelines, we report here the measures used in the two systematic reviews that summarized the pooled results of most of these studies (3,6).

PRISMA-ScR Checklist*

Section/topic	#	Checklist item	Reported on page #
**TITLE**			
Title	1	Identify the report as a scoping review.	1
**ABSTRACT**			
Structured summary	2	Provide a structured summary that includes (as applicable) background, objectives, eligibility criteria, sources of evidence, charting methods, results, and conclusions that relate to the review questions and objectives.	1,2
**INTRODUCTION**			
Rationale	3	Describe the rationale for the review in the context of what is already known. Explain why the review questions/objectives lend themselves to a scoping review approach.	3,4 ("Background")
Objectives	4	Provide an explicit statement of the questions and objectives being addressed with reference to their key elements (e.g., population or participants, concepts, and context) or other relevant key elements used to conceptualize the review questions and/or objectives.	5 ("Objectives")
**METHODS**			
Protocol and registration	5	Indicate whether a review protocol exists; state if and where it can be accessed (e.g., a Web address); and if available, provide registration information, including the registration number.	5 ("Methods")
Eligibility criteria	6	Specify characteristics of the sources of evidence used as eligibility criteria (e.g., years considered, language, and publication status), and provide a rationale.	5,6 ("Eligibility Criteria", "Exclusion Criteria")
Information sources	7	Describe all information sources in the search (e.g., databases with dates of coverage and contact with authors to identify additional sources), as well as the date the most recent search was executed.	6 ("Search Strategy and Study Selection")
Search	8	Present the full electronic search strategy for at least 1 database, including any limits used, such that it could be repeated.	6 ("Search Strategy and Study Selection")
Study selection	9	State the process for selecting sources of evidence (i.e., screening and eligibility) included in the scoping review.	6,7 ("Search Strategy and Study

For peer review only

PRISMA-ScR Checklist*

			Selection")
Data collection process	10	Describe the methods of charting data from the included sources of evidence (e.g., calibrated forms or forms that have been tested by the team before their use, and whether data charting was done independently or in duplicate) and any processes for obtaining and confirming data from investigators.	8 ("Data Extraction")
Data items	11	List and define all variables for which data were sought and any assumptions and simplifications made.	8 ("Data Extraction")
Risk of bias in individual studies	12	If done, provide a rationale for conducting a critical appraisal of included sources of evidence; describe the methods used and how this information was used in any data synthesis (if appropriate).	N/A
Summary measures	13	Not applicable for scoping reviews.	N/A
Synthesis of results	14	Describe the methods of handling and summarizing the data that were charted.	9 ("Data Synthesis)

Page 1 of 2

Section/topic	#	Checklist item 10	Reported on page #
Risk of bias across studies	15	Not applicable for scoping reviews.	N/A
Additional analyses	16	Not applicable for scoping reviews.	N/A
**RESULTS**			
Study selection	17	Give numbers of sources of evidence screened, assessed for eligibility, and included in the review, with reasons for exclusions at each stage, ideally using a flow diagram.	Figure 1
Study characteristics	18	For each source of evidence, present characteristics for which data were charted and provide the citations.	10 ("Results)
Risk of bias within studies	19	If done, present data on critical appraisal of included sources of evidence (see item 12).	N/A
Results of individual studies	20	For each included source of evidence, present the relevant data that were charted that relate to the review questions and objectives.	10 ("Results")
Synthesis of results	21	Summarize and/or present the charting results as they relate to the review questions and objectives.	10-15 ("Results")

For peer review only - http://bmjopen.bmj.com/site/about/guidelines.xhtml

PRISMA-ScR Checklist*

Risk of bias across studies	22	Not applicable for scoping reviews.	
Additional analysis	23	Not applicable for scoping reviews.	
**DISCUSSION**			
Summary of evidence	24	Summarize the main results (including an overview of concepts, themes, and types of evidence available), link to the review questions and objectives, and consider the relevance to key groups.	15 (“Discussion)
Limitations	25	Discuss the limitations of the scoping review process.	17 (“Discussion”)
Conclusions	26	Provide a general interpretation of the results with respect to the review questions and objectives, as well as potential implications and/or next steps.	15-17 (“Discussion)
**FUNDING**			
Funding	27	Describe sources of funding for the included sources of evidence, as well as sources of funding for the scoping review. Describe the role of the funders of the scoping review.	18 (“Declarations – Funding”)

**Original source: http://annals.org/aim/fullarticle/2700389/prisma-extension-scoping-reviews-prisma-scr-checklist-explanation*

For peer review only

For peer review only - http://bmjopen.bmj.com/site/about/guidelines.xhtml
